# Silicon Combined with Activated Carbon Enhances Salt Tolerance in Strawberry (*Fragaria* × *ananassa*) by Reinforcing Ion–Redox Homeostasis and Reshaping the Rhizosphere Microbiome

**DOI:** 10.3390/plants15081154

**Published:** 2026-04-09

**Authors:** Chendong Sun, Zhaoxin Ge, Xiaofang Yang, Xiaobo Xie, Xinyi Liang, Lan Shen, Jianjie Ren, Yuchao Zhang

**Affiliations:** 1 The Institute of Horticulture, Zhejiang Academy of Agricultural Sciences, Hangzhou 310021, China; 17661362973@163.com (Z.G.); yangxiaofang1981@yeah.net (X.Y.); xiexb@zaas.ac.cn (X.X.); 15532472609@163.com (X.L.); 2 School of Ecological Technology and Engineering, Shanghai Institute of Technology, Shanghai 201418, China; 3 Institute of Biotechnology, Ningbo Academy of Agricultural Sciences, Ningbo 315040, China; shenlan5056@163.com; 4 Agricultural and Rural Affairs Bureau of Shangyu District, Shaoxing 312300, China; solomon_2001@163.com

**Keywords:** salinity stress, potassium silicate, soil amendment, Na^+^/K^+^ balance, reactive oxygen species (ROS), 16S rRNA amplicon sequencing, transcriptome reprogramming

## Abstract

Soil salinity severely constrains strawberry production by disrupting ion homeostasis and provoking oxidative injury. This study investigated whether soluble silicon (Si) and activated carbon (AC) act to enhance salt tolerance in strawberry (*Fragaria* × *ananassa*). Under NaCl stress, plants showed pronounced growth inhibition, increased Na^+^ accumulation and a deteriorated K^+^/Na^+^ balance, accompanied by elevated reactive oxygen species (ROS) and lipid peroxidation. In contrast, combined AC + Si treatment consistently provided the strongest protection, improving seedling vigor and survival. Relative to NaCl alone, AC + Si increased shoot and root fresh weight by 67.5% and 78.5%, reduced shoot Na^+^ by 59.1%, and lowered shoot H_2_O_2_ and MDA by 62.6% and 66.5%, respectively, indicating marked improvement in ion–redox homeostasis. Beyond plant responses, AC-containing treatments alleviated salt-induced increases in soil electrical conductivity, coinciding with a clear restructuring of the rhizosphere bacterial community and enrichment of putatively beneficial taxa. Transcriptome profiling further supported coordinated reprogramming of ion transport, redox control and stress-responsive signaling pathways under the AC + Si regime. Collectively, the results indicated that Si and AC co-application enhances strawberry salt tolerance through an integrated soil–plant–microbiome mechanism that stabilizes ion homeostasis and reinforces redox homeostasis.

## 1. Introduction

Soil salinization is expanding across irrigated and coastal agricultural regions, posing a constraint on sustainable horticultural production. Salt stress imposes a rapid osmotic phase that limits growth, followed by a slower ionic phase characterized by toxic Na^+^ and Cl^−^ accumulation and accelerated senescence [1]. Salinity disrupts cellular ion balance, impairs photosynthesis, and promotes excessive reactive oxygen species (ROS) accumulation and oxidative stress, ultimately constraining biomass accumulation and yield [1,2,3]. Because salinity tolerance integrates growth, water relations, ion homeostasis, and stress physiology, carefully designed phenotyping and rigorous phenotyping frameworks and appropriate tolerance indices are essential for interpreting treatment effects and identifying causal traits [4].

At the mechanistic level, maintaining low cytosolic Na^+^ and adequate K^+^ is central to salinity tolerance. Plants restrict Na^+^ entry, extrude Na^+^ from the cytosol, retrieve Na^+^ from the xylem, and compartmentalize Na^+^ in vacuoles to reduce cytotoxicity [5]. These ion-transport processes are embedded within broader stress signaling networks that coordinate ion and water transport, metabolic adjustment, and gene-expression reprogramming [5]. In parallel, ROS homeostasis and antioxidant defenses limit membrane damage and help maintain cellular stability during salt exposure, in concert with hormonal and transcriptional programs that shape whole-plant outcomes [6]. Practical salt-mitigation strategies that can consistently improve performance often converge on two axes: improved Na^+^/K^+^ homeostasis and water status together with reinforced redox balance and stress resilience.

Cultivated strawberry (*Fragaria* × *ananassa* Duch.) is generally regarded as salt-sensitive, and saline irrigation water or saline soils can markedly compromise plant vigor and productivity [7,8,9,10]. Although the molecular basis of strawberry salt responses is still being refined, transcriptome-level evidence indicates that osmotic stresses (including salinity) reshape stress-response pathways and antioxidant-related processes in strawberry tissues, highlighting the relevance of ion–redox coordination for performance under stress [11]. However, translating these molecular signatures to tractable, root-zone interventions remains challenging. Strawberry is grown in diverse substrates and soil-management regimes where rhizosphere physicochemistry and microbial communities can strongly modulate stress responses, making it important to evaluate salt-mitigation approaches through an integrated plant–soil–microbiome framework.

Among agronomically feasible interventions, silicon (Si) is widely studied as a beneficial element that can enhance tolerance to abiotic stresses, including salinity. Reviews synthesizing multi-species evidence indicate that Si improves plant water status and salinity performance through multiple, context-dependent mechanisms, including reduced Na^+^ uptake/translocation, improved K^+^ nutrition, enhanced antioxidant capacity, and altered stress-responsive gene expression [2,12]. Mechanistic models further propose that Si-mediated improvements in water relations can involve coordinated regulation of aquaporins and root hydraulic conductance under salinity, thereby supporting water uptake during osmotic stress [13]. Recent evidence indicates that Si can mitigate salt-induced declines in root hydraulic conductivity and modulate aquaporin-related water transport under salinity [2,14]. Collectively, these data support Si as a practical lever to enhance the water–ion axis of salt tolerance, while emphasizing the need to define effective Si forms and regimes for specific crops and production settings.

In parallel, carbon-based soil amendments—most prominently biochar—have attracted attention for improving soil physical–chemical properties and promoting plant performance under drought and salt stress. A comprehensive review indicates that biochar can alleviate salt stress by improving soil water-holding capacity and associated soil functions and by influencing plant ion relations (often reducing Na^+^ uptake while supporting K^+^ uptake), stomatal behavior, and phytohormonal regulation, although outcomes depend on biochar type and application rate [15,16]. Beyond abiotic stress mitigation, biochar can reshape soil microbial communities and has been reviewed as a contributor to pathogen suppression and induced plant defenses through changes in soil properties, root exudation, and microbial ecology [17]. These features also inform expectations for activated carbon–like materials because high surface area and sorptive properties can alter nutrient/ion availability and create microhabitats that influence microbial assembly in the rhizosphere.

The rhizosphere microbiome is recognized as a major determinant of plant performance, including stress tolerance. Recent reviews indicate that plants actively shape their rhizosphere microbiome and that root-associated communities contribute to plant health, productivity, and stress acclimation [18,19,20]. Across crop systems, root microbiome assembly follows a multistep process with strong compartmentalization among rhizosphere, rhizoplane, and endosphere communities and sensitivity to soil source and cultivation practices [20]. At the mechanistic interface, beneficial microbes can induce systemic resistance and prime plant defense pathways, and contribute to stress resilience under adverse conditions [21]. Thus, management practices that shift rhizosphere communities toward beneficial consortia may provide durable leverage for stress mitigation.

A particularly relevant class of beneficial microbes are plant growth-promoting bacteria (PGPB), which can enhance plant performance under salinity and drought through multiple mechanisms. Reviews emphasize that PGPB can modulate phytohormone balance, improve nutrient acquisition, produce osmoprotective compounds, reinforce antioxidant defenses, and suppress pathogens, thereby contributing to plant performance under salinity and other abiotic stresses [21,22]. Among these mechanisms, bacterial 1-aminocyclopropane-1-carboxylate (ACC) deaminase is widely discussed as a stress-modulating trait: by lowering plant ACC (the ethylene precursor), ACC deaminase-positive bacteria can reduce stress-induced ethylene accumulation and associated growth inhibition, contributing to improved tolerance under salinity and other stresses [21,22,23]. Accordingly, strategies that enrich or introduce beneficial bacteria with such traits may complement mineral and soil amendments targeting the water–ion axis.

Recent synthesis points to the potential complementarity of combining biochar with PGPB, reporting that co-application often yields greater improvements in crop performance than either input alone and proposing mechanistic bases for positive interactions between biochar-mediated habitat modification and microbial functions [24,25]. In addition, studies integrating biochar with specific functional microbes show that “microbe-loaded” biochar can enhance soil enzyme activity and reshape microbial community features, illustrating a plausible route by which carbonaceous amendments can act as microbial carriers or ecological filters [25,26]. These findings motivate an integrated hypothesis for strawberry that combining silicon-based interventions with carbonaceous amendments may yield additive or non-additive improvements under salinity. Despite the conceptual appeal, evidence remains limited for integrated “Si + carbonaceous amendment” strategies in strawberry under progressive salinity regimens, particularly with multi-layered evaluation linking plant phenotypes to rhizosphere microbiome shifts and ion–redox regulatory signatures. Salinity in production systems is often gradual and cumulative, and strawberry performance is especially sensitive to root-zone conditions and microbial interactions. The present study evaluated whether combining silicon supply with a carbon-based amendment improves strawberry performance under salt stress and whether these benefits align with enrichment of beneficial rhizosphere bacteria and activation of host ion–redox programs, providing a mechanistic framework for practical management in salt-affected cultivation systems. On this basis, the following hypotheses were tested: (H1) under progressive salinity, the combined application of potassium silicate and a carbon-based amendment would improve strawberry performance (seedling survival and biomass) compared with salinity alone and would show stronger benefits than either input applied separately; (H2) these benefits would be associated with improved ion and redox homeostasis, reflected by reduced shoot Na^+^ accumulation and Na^+^/K^+^ ratio, lower oxidative damage (ROS and MDA), and enhanced antioxidant activities; and (H3) AC-containing treatments would restructure the rhizosphere bacterial community and the combined AC + Si regime would be accompanied by an early transcriptomic shift in pathways related to ion transport and redox regulation, consistent with the observed physiological phenotypes.

## 2. Materials and Methods

### 2.1. Plant Materials and Growth Conditions

Tissue-cultured strawberry (*Fragaria* × *ananassa* Duch.) plantlets (cv. *Jiandebailu*) were rooted for ~30 d, and uniform plantlets with comparable vigor were selected. Plants were transplanted individually into 6.5 cm plastic pots containing a sterilized substrate mixture of peat:vermiculite:perlite (3:3:1, *v*/*v*/*v*) and cultivated in a plant factory located in Shangyu District, Shaoxing, Zhejiang Province, China. Pots were maintained in a controlled-environment growth chamber under a 12 h light/12 h dark photoperiod with an irradiance of 12,000 lux, and the air temperature was kept constant at 25 °C during both the light and dark periods (25/25 °C, day/night). During acclimation, plants were watered every three days with half-strength Murashige and Skoog solution (1/2 MS).

### 2.2. Preparation of Activated Carbon-Amended Substrate

Commercial activated carbon (AC; OriLeaf Bio-Technology Co., Ltd., Shanghai, China; product no. W14584) was washed thoroughly with deionized water and oven-dried prior to use. The physicochemical properties of the AC were further characterized by the Analytical and Testing Center (Anhui University of Science & Technology), SCI-GO (www.sci-go.com accessed on 25 October 2025). The key characterization results are summarized in Table 1. Briefly, the material showed a BET specific surface area of 950.0082 m^2^·g^−1^, a total pore volume of 0.414170 cm^3^·g^−1^, a micropore volume of 0.321489 cm^3^·g^−1^, and an average pore diameter of 1.7439 nm based on N_2_ adsorption analysis, together with a pH of 8.3 and an ash content of 3.82%. The full analytical report is provided as Supplementary File S1. AC was incorporated into the substrate at 2% (*w*/*w*, dry weight basis), and the amended substrate was mixed thoroughly and equilibrated for 5 d before microbial inoculation.

### 2.3. Preparation of Saline–Alkali Soil Microbial Inoculum and Substrate Inoculation

A microbial consortium was enriched from saline–alkali strawberry soil collected in the Dajiangdong area, Qiantang District, Hangzhou, Zhejiang Province, China. Soil suspensions were used to inoculate Luria–Bertani (LB) broth and incubated at 28 °C with shaking (180 rpm) for 48 h. Cells were harvested by centrifugation (8000× *g*, 10 min), washed twice with sterile water, and resuspended to an optical density at 600 nm (OD600) = 1.0. The standardized suspension was added to both AC-amended and AC-free substrates, mixed thoroughly to ensure homogeneous inoculation, sealed to maintain moisture, and incubated for 7 d prior to transplanting.

### 2.4. Experimental Design and Salt/Silicon Treatments

The experiment followed a completely randomized design with five treatments: Control, NaCl, NaCl + Si, NaCl + AC, and NaCl + AC + Si, with 70 pots per treatment (one plant per pot) to accommodate destructive sampling and phenotyping. After a 14-d acclimation period, silicon was supplied as potassium silicate (K_2_SiO_3_) at 1.5 mM; to control for potassium input, non-silicon treatments received KCl at an equimolar K^+^ dose (3 mM KCl). Salt stress was imposed stepwise to minimize osmotic shock by irrigating salt-stressed plants with 1/2 MS containing 60 mM NaCl (Day 15), 100 mM NaCl (Day 16), and 150 mM NaCl (Day 17), while control plants received 1/2 MS without NaCl throughout; excess drainage in trays was promptly removed to avoid unintended bottom soaking. In a preliminary dose–response screening, runner-propagated production seedlings from eight strawberry cultivars were exposed to six NaCl concentrations (0, 25, 50, 100, 150, and 200 mM). For cv. ‘*Jiandebailu*’, visible salt-injury symptoms (leaf-margin chlorosis/necrosis, dehydration, and leaf curling) became apparent after 14 d at 150 mM NaCl (Figure S4); therefore, 150 mM was selected as the target severe-salinity level for the present study. Because the current experiment used in vitro–rooted plantlets (smaller and potentially more salt-sensitive than production seedlings), NaCl was applied by stepwise acclimation (60 mM on day 1, 100 mM on day 2, and 150 mM on day 3) to minimize osmotic shock and improve treatment reproducibility.

### 2.5. Sampling Scheme

For soil physicochemical analyses, substrate was sampled at two time points: (1) after microbial incubation but prior to transplanting, where AC(+) and AC(−) substrates were each sampled in triplicate for EC, pH, and Na/K determinations; and (2) after 7 d at 150 mM NaCl, where substrate from each of the five treatments was collected from three pots per treatment for EC, pH, and Na/K measurements.

### 2.6. Growth Traits and Survival Assessment

At the endpoint of the salt treatment, shoots and roots were separated and fresh weight was determined on an individual-plant basis (*n* = 12 plants per treatment). Seedling survival was evaluated under 150 mM NaCl using independent plants (*n* = 40 plants per treatment) and expressed as survival rate (%). For survival scoring, a plant was considered alive when it retained ≥2 fully expanded green leaves with no visible marginal necrosis, chlorosis, or wilting; plants not meeting this criterion were scored as dead.

### 2.7. Soil Physicochemical Properties (EC, pH, and Soil Ions)

Soil/substrate samples were collected at two time points: (1) after microbial incubation but prior to transplanting, where AC(+) and AC(−) substrates were each sampled in triplicate; and (2) after 7 d at 150 mM NaCl, where substrate from each of the five treatments was collected (three pots per treatment) for physicochemical analyses. For EC and pH determination, 5 g of fresh substrate was extracted with 25 mL deionized water (1:5, *w*/*v*), and EC and pH were measured in the slurry using a portable multiparameter meter (HI98195, Hanna Instruments, Woonsocket, RI, USA). Aliquots of the same soil–water extract were clarified (by settling and/or filtration) and analyzed for Na and K using inductively coupled plasma optical emission spectrometry (ICP-OES) (iCAP Pro X, Thermo Fisher Scientific, Waltham, MA, USA).

### 2.8. Determination of Na^+^ and K^+^ in Plant Tissues

Shoot and root tissues were harvested separately, briefly rinsed with deionized water, and oven-dried at 80 °C to constant weight. Dried samples were ground to a fine powder, and 0.10 g of each sample was wet-digested with concentrated H_2_SO_4_ using H_2_O_2_ as an oxidant at 280 °C until the solution became clear. The digests were filtered and brought to a final volume of 50 mL with deionized water, and Na^+^ and K^+^ concentrations were quantified using ICP-OES (iCAP Pro X, Thermo Fisher Scientific, Waltham, MA, USA). The K^+^/Na^+^ ratio was calculated accordingly.

### 2.9. Oxidative Stress Markers (MDA, H_2_O_2_, and O_2_·^−^)

At the endpoint of the salt treatment, fresh leaves were collected, immediately frozen in liquid nitrogen, and stored at −80 °C; all assays were completed within 2 weeks. Malondialdehyde (MDA) content was determined using a commercial kit (Suzhou Comin Biotechnology Co., Ltd., Suzhou, China; MDA-1-Y) based on the thiobarbituric acid (TBA) reaction: ~0.1 g tissue was homogenized on ice in 1 mL extraction buffer, centrifuged (8000× *g*, 4 °C, 10 min), and the supernatant was processed following the manufacturer’s protocol; absorbance was recorded at 532 and 600 nm using a microplate reader (Spark^®^, Tecan Group Ltd., Männedorf, Switzerland). Superoxide anion (O_2_·^−^) level/production rate was quantified with a hydroxylamine-based kit (Suzhou Comin Biotechnology Co., Ltd., Suzhou, China; SA-1); ~0.1 g tissue was extracted on ice in 1 mL extraction buffer, centrifuged (10,000× *g*, 4 °C, 20 min), and absorbance was measured at 530 nm. Hydrogen peroxide (H_2_O_2_) content was determined using a titanium sulfate microplate kit (Beijing Leagene Biotechnology Co., Ltd., Beijing, China; TO1075) and read at 412 nm (Spark^®^, Tecan Group Ltd., Männedorf, Switzerland), all according to the manufacturers’ instructions.

### 2.10. Rhizosphere Microbiome Sampling, 16S rRNA Sequencing, and Bioinformatics

Rhizosphere samples were collected after 7 d of 150 mM NaCl treatment by gently removing loosely attached substrate, followed by resuspension of tightly adhering rhizosphere soil in sterile water and centrifugation to obtain a rhizosphere pellet for downstream DNA-based analyses. Genomic DNA extraction, PCR amplification, library construction, Illumina sequencing, and primary bioinformatics were performed by Shanghai Majorbio Bio-pharm Technology Co., Ltd. (Shanghai, China). Briefly, bacterial 16S rRNA gene amplicons were generated using a two-step PCR strategy with two primer sets (first round: 799F/1392R; second round: 799F/1193R); primer sequences were 799F (AACMGGATTAGATACCCKG) and 1193R (ACGTCATCCCCACCTTCC), and PCR was conducted with an annealing temperature of 55 °C and a unified cycle number across samples for the formal amplification (13 cycles in the second round). Libraries were sequenced on an Illumina NextSeq 2000 platform (Illumina, San Diego, CA, USA) in paired-end mode (paired-end 300 bp (PE300)). Amplicon reads were processed to generate amplicon sequence variants (ASVs) using Quantitative Insights Into Microbial Ecology 2 (QIIME 2) with DADA2 denoising (including quality control and chimera removal), and taxonomic assignment was performed against the SILVA database [28,29,30]. The resulting ASV table and taxonomy annotations were subsequently analyzed in R, including alpha diversity estimation, Bray–Curtis dissimilarity-based ordination and permutational multivariate analysis of variance (PERMANOVA), and differential abundance testing using ANCOM-BC with false discovery rate control [31,32]. Raw sequencing reads have been deposited in the NCBI Sequence Read Archive under BioProject accession PRJNA1424400.

### 2.11. RNA Extraction, Library Construction, RNA-Seq Data Processing, and Heatmap Visualization

For transcriptome profiling, total RNA was extracted from treated strawberry tissues using TRIzol^®^ reagent (Thermo Fisher Scientific, Waltham, MA, USA) following the manufacturer’s instructions, quantified spectrophotometrically, and assessed for integrity using an Agilent Bioanalyzer; only high-quality RNA was used for subsequent library construction. RNA purification, strand-specific mRNA library preparation and Illumina sequencing were performed by Shanghai Majorbio Bio-pharm Technology Co., Ltd. (Shanghai, China), including poly(A)^+^ mRNA enrichment, fragmentation, cDNA synthesis, end repair, adapter ligation, size selection, and PCR enrichment. After adapter trimming and quality filtering with fastp [33], clean reads were aligned to the octoploid cultivated strawberry (*Fragaria* × *ananassa*) ‘Benihoppe’ reference genome [34] using HISAT2 [35] with reverse–forward (RF) strand-specific orientation, and the resulting alignments were processed with SAMtools [36]. Reference-guided transcript assembly and quantification were carried out using StringTie [37], and gene-level count matrices were generated for downstream differential expression analysis in DESeq2 with Benjamini–Hochberg false discovery rate control [38]. DEGs were defined as |log2(fold change)| ≥ 1 (fold change ≥ 2) with an adjusted *p* value (FDR) < 0.01 for downstream enrichment and gene-level visualization. The Gene Ontology (GO) and Kyoto Encyclopedia of Genes and Genomes (KEGG) annotations for strawberry genes were performed using the online software EggNOG-Mapper v2.1.13 (https://eggnog6.embl.de (accessed on 25 October 2025)), while GO and KEGG enrichment analyses were performed using TBtools (Toolkit for Biologists) software (v2.460) [39]. For visualization of selected genes, heatmaps were generated in R using the pheatmap package based on log_2_ (fragments per kilobase of transcript per million mapped reads, FPKM + 1)–transformed expression values; to facilitate cross-treatment comparison, expression values were centered per gene by subtracting the corresponding Control mean (thereby displaying relative changes from Control), with hierarchical clustering applied to genes and sample columns arranged according to the experimental design. Raw sequencing reads have been deposited in the NCBI Sequence Read Archive under BioProject accession PRJNA1424399.

### 2.12. Statistical Analysis

All statistical analyses were conducted in R (v4.5.1; R Foundation for Statistical Computing, Vienna, Austria). Survival rate (binary outcome) was analyzed using pairwise Fisher’s exact tests with Holm adjustment for multiple comparisons, and 95% binomial confidence intervals were calculated; significance groupings were displayed as compact letter displays. For continuous traits (fresh weight, H_2_O_2_, MDA, O_2_·^−^, plant Na^+^/K^+^ and Na^+^/K^+^ ratio, soil EC/pH, and soil Na^+^/K^+^), treatment effects were evaluated by one-way ANOVA followed by Tukey’s HSD for all-pair comparisons, and Dunnett-type comparisons versus the Control were additionally reported where applicable [40]. To statistically evaluate non-additive (interaction) effects of Si and AC under salinity, a factorial two-way ANOVA (Si × AC) was additionally performed using only the NaCl-containing treatments (NaCl, NaCl + Si, NaCl + AC, and NaCl + AC + Si), with *p* values reported for the main effects of Si and AC and their interaction term (Si × AC) (Table S4). Rhizosphere microbiome community analyses were performed in R from the processed ASV table, including alpha diversity estimation, Bray–Curtis dissimilarity-based ordination, and PERMANOVA (vegan), while differential abundance testing was performed using ANCOM-BC2 with false discovery rate control [32]. In all analyses, differences were considered significant at *p* < 0.05.

## 3. Results

### 3.1. Seedling Survival and Growth Responses to Silicon and Activated Carbon Under Salinity

**Figure 1:**
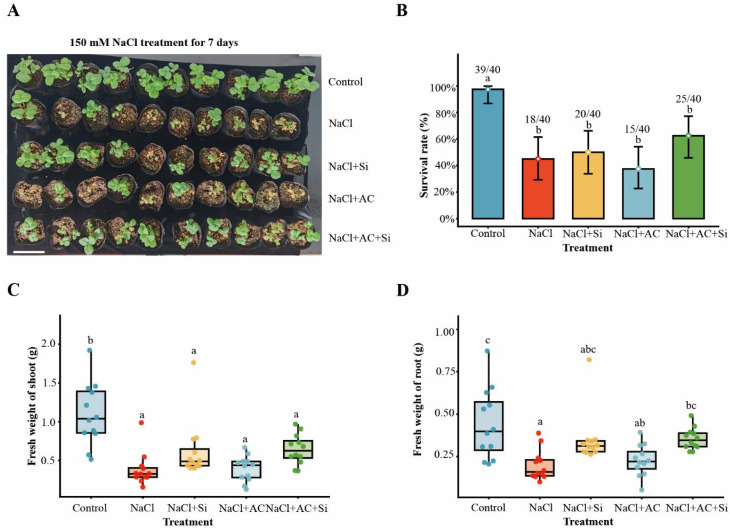
Effects of activated carbon (AC) and silicon (Si; supplied as potassium silicate, K_2_SiO_3_) on strawberry plant performance under salt stress. (**A**) Representative phenotypes after 150 mM NaCl treatment (10 d) under the indicated treatments. The white line segment indicates a scale bar of 6 cm. (**B**) Survival rate after 150 mM NaCl (10 d). Numbers above bars indicate surviving plants/total plants. Error bars represent 95% binomial confidence intervals. Survival (binary outcome) was analyzed using pairwise Fisher’s exact tests with Holm adjustment for multiple comparisons; significance groupings are displayed as compact letter displays. Different letters indicate significant differences at *p* < 0.05. (**C**) Shoot fresh weight. (**D**) Root fresh weight. Continuous traits were evaluated by one-way analysis of variance (ANOVA) followed by Tukey’s honestly significant difference (HSD) test for all-pair comparisons; Dunnett-type comparisons versus the Control were additionally reported where applicable. Different letters indicate significant differences at *p* < 0.05.

The experimental design, treatment regime, and sampling workflow are summarized in Figure S1. Under severe salinity (150 mM sodium chloride, NaCl), strawberry seedlings exhibited pronounced injury symptoms compared with the non-saline control (Figure 1A). Compared with NaCl alone, the combined AC + Si treatment showed the strongest improvement in survival and fresh biomass, whereas Si or AC alone produced smaller and/or more variable effects (Figure 1B–D). NaCl markedly reduced survival, whereas the combined treatment with activated carbon (AC) and silicon (Si; supplied as potassium silicate, K_2_SiO_3_) improved survival from 32.5% under NaCl to 67.5% under AC + Si (Figure 1B). Consistently, salt stress substantially decreased shoot and root fresh weights, and AC + Si partially restored biomass among salt-stressed plants (Figure 1C,D). Replicate-level raw data, summary statistics, percent changes relative to NaCl, and factorial interaction statistics are provided in Table S1, Table S2, Table S3, and Table S4, respectively. Relative to NaCl alone, AC + Si increased survival, shoot fresh weight, and root fresh weight, with shoot and root fresh weight increasing by 67.5% and 78.5%, respectively.

### 3.2. Modulation of Soil Physicochemical Properties and the Rhizosphere Bacterial Community by Single and Combined Applications of Silicon and Activated Carbon Under Salinity

**Figure 2:**
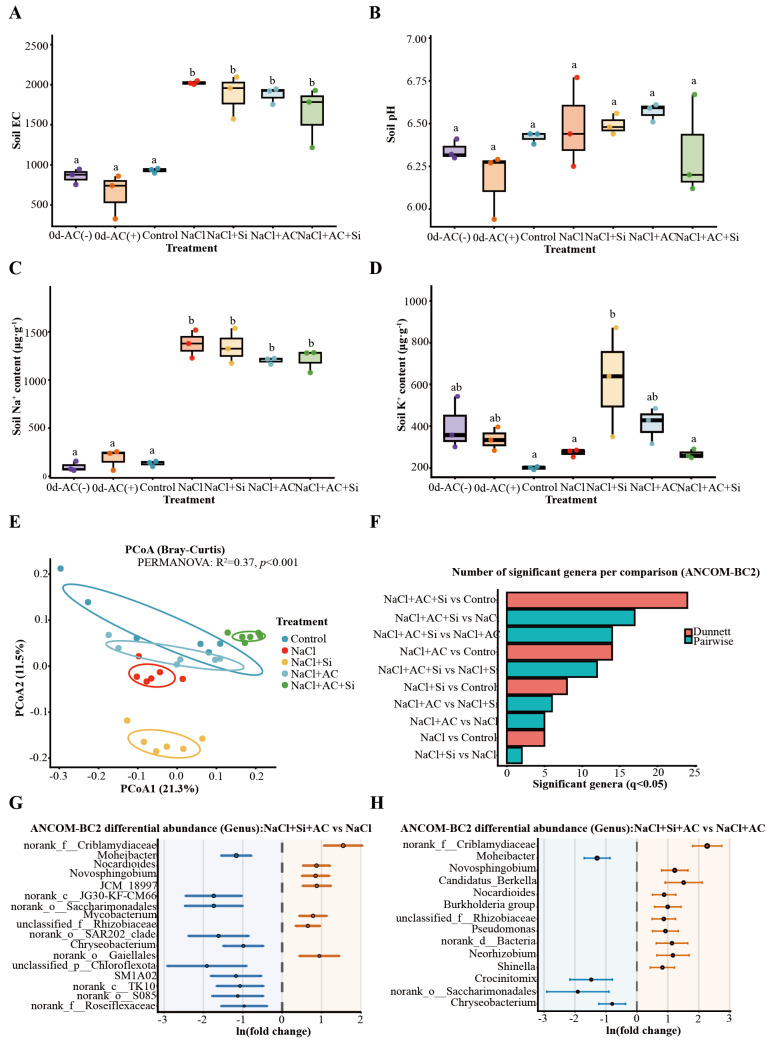
Effects of AC and Si on soil physicochemical properties and rhizosphere bacterial community composition. (**A**) Soil electrical conductivity (EC). (**B**) Soil pH. (**C**) Soil Na^+^ content. (**D**) Soil K^+^ content. “0 d-AC(−)” and “0 d-AC(+)” indicate baseline soils before planting under AC-free and AC-amended conditions, respectively. Soil traits were analyzed by one-way ANOVA followed by Tukey’s HSD; Dunnett-type comparisons versus the Control were additionally reported where applicable. Different letters indicate significant differences at *p* < 0.05. (**E**) Principal coordinates analysis (PCoA) based on Bray–Curtis dissimilarity of rhizosphere bacterial communities; permutational multivariate analysis of variance (PERMANOVA; vegan) statistics are shown in the panel. (**F**) Number of significantly differential genera per comparison identified by ANCOM-BC2 with false discovery rate control; significance threshold *q* < 0.05. (**G**,**H**) Genus-level differential abundance (ANCOM-BC2) for NaCl + AC + Si vs. NaCl (**G**) and NaCl + AC + Si vs. NaCl + AC (**H**), presented as ln(fold change) with confidence intervals; full outputs are provided in Table S6. In (**H**), the sixth taxon label “Burkholderia–Caballeronia–Paraburkholderia” is a condensed/combined genus annotation (Burkholderia sensu lato complex) commonly used in 16S genus-level reporting to represent closely related genera that may not be reliably separated at this marker resolution.

Soil electrical conductivity (EC) increased strongly under NaCl, confirming successful establishment of salinity stress; among salt-treated groups, AC + Si showed the most consistent improvement in soil physicochemical status and rhizosphere community structure relative to NaCl, whereas single amendments (Si or AC) produced weaker or more variable shifts; correspondingly, the AC + Si group exhibited comparatively lower EC than the other NaCl-containing treatments (Figure 2A). Soil pH varied within a narrow range across treatments (Figure 2B). Soil Na^+^ content increased sharply under salinity and was broadly comparable among the salt-treated groups (Figure 2C), whereas soil K^+^ differed among treatments and tended to be higher in the Si-containing treatments (Figure 2D). For rhizosphere bacterial profiling, sequencing depth and basic quality metrics indicated adequate coverage across samples (Table S5). Community structure differed clearly among treatments, as shown by Bray–Curtis principal coordinates analysis (PCoA) (Figure 2E). Differential abundance analysis revealed that comparisons involving AC + Si yielded the greatest number of significantly shifted genera (Figure 2F). In particular, AC + Si differed from NaCl and from NaCl + AC with multiple genera showing significant shifts, including signals involving *Pseudomonas* and *Neorhizobium* (Figure 2G,H; Table S6). Alpha-diversity indices and genus-level composition profiles further supported treatment-associated restructuring of rhizosphere bacterial assemblages (Figure S2A–C). Relative to NaCl alone, soil EC was reduced by 18.8% under AC + Si.

### 3.3. Changes in Ion Homeostasis in Response to Silicon and Activated Carbon Under Salinity

**Figure 3:**
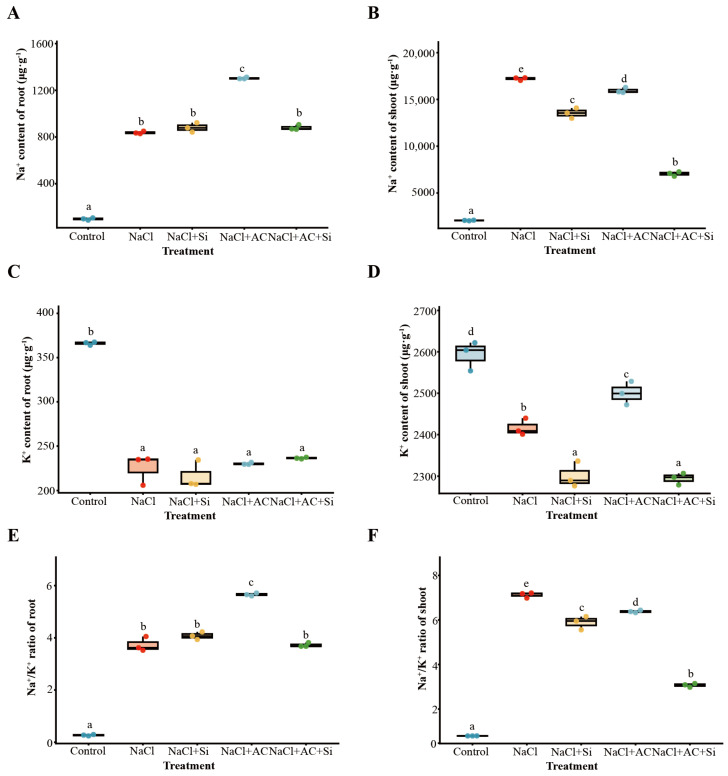
Effects of AC and Si on ion homeostasis in strawberry plants under salt stress. (**A**) Root Na^+^ content. (**B**) Shoot Na^+^ content. (**C**) Root K^+^ content. (**D**) Shoot K^+^ content. (**E**) Root Na^+^/K^+^ ratio. (**F**) Shoot Na^+^/K^+^ ratio. Continuous traits were analyzed by one-way ANOVA followed by Tukey’s HSD; Dunnett-type comparisons versus the Control were additionally reported where applicable. Different letters indicate significant differences at *p* < 0.05.

Salt stress induced strong Na^+^ accumulation in both roots and shoots (Figure 3A,B) and altered K^+^ status relative to the control (Figure 3C,D). Notably, the combined AC + Si treatment reduced shoot Na^+^ relative to NaCl (Figure 3B). Na^+^/K^+^ ratios increased substantially under salinity in both roots and shoots (Figure 3E,F), whereas AC + Si produced the most consistent reduction of the shoot Na^+^/K^+^ ratio among salt-treated plants (Figure 3F), indicating improved ionic balance in aboveground tissues. Relative to NaCl alone, shoot Na^+^ was reduced by 59.1% under AC + Si. Consistent with a non-additive combined effect, factorial two-way ANOVA under salinity detected significant Si×AC interactions for Na^+^ accumulation in roots and shoots (Table S4).

### 3.4. Oxidative Stress Marker Responses to Silicon and Activated Carbon Under Salinity

**Figure 4:**
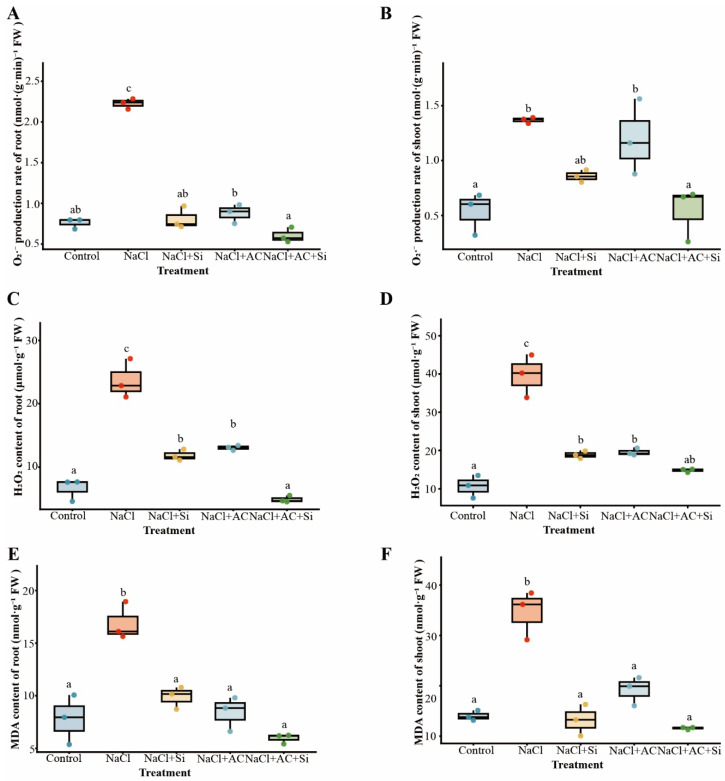
Effects of AC and Si on oxidative stress markers in strawberry plants under salt stress. (**A**) Root O_2_·^−^ production rate. (**B**) Shoot O_2_·^−^ production rate. (**C**) Root H_2_O_2_ content. (**D**) Shoot H_2_O_2_ content. (**E**) Root MDA content. (**F**) Shoot MDA content. Continuous traits were analyzed by one-way ANOVA followed by Tukey’s HSD; Dunnett-type comparisons versus the Control were additionally reported where applicable. Different letters indicate significant differences at *p* < 0.05.

Salt stress markedly increased oxidative stress indicators. O_2_·^−^ production rates rose in both roots and shoots under NaCl, whereas AC + Si reduced O_2_·^−^ among salt-treated plants (Figure 4A,B). H_2_O_2_ content similarly increased under NaCl and was reduced under AC + Si in both tissues, with a clearer attenuation in roots (Figure 4C,D). Lipid peroxidation, reflected by malondialdehyde (MDA) accumulation, was enhanced by salinity and was comparatively lower under AC + Si among the salt treatments (Figure 4E,F), supporting reduced oxidative injury under the combined amendment. Relative to NaCl alone, AC + Si reduced root O_2_·^−^ by 72.9%, shoot H_2_O_2_ by 62.6%, and MDA by 64.8% in roots and 66.5% in shoots. Two-way ANOVA further indicated significant Si×AC interactions for several oxidative stress readouts, including shoot H_2_O_2_, root O_2_·^−^, and MDA in both tissues (Table S4).

### 3.5. Transcriptomic Signatures Associated with Silicon and Activated Carbon Treatments Under Salinity

**Figure 5:**
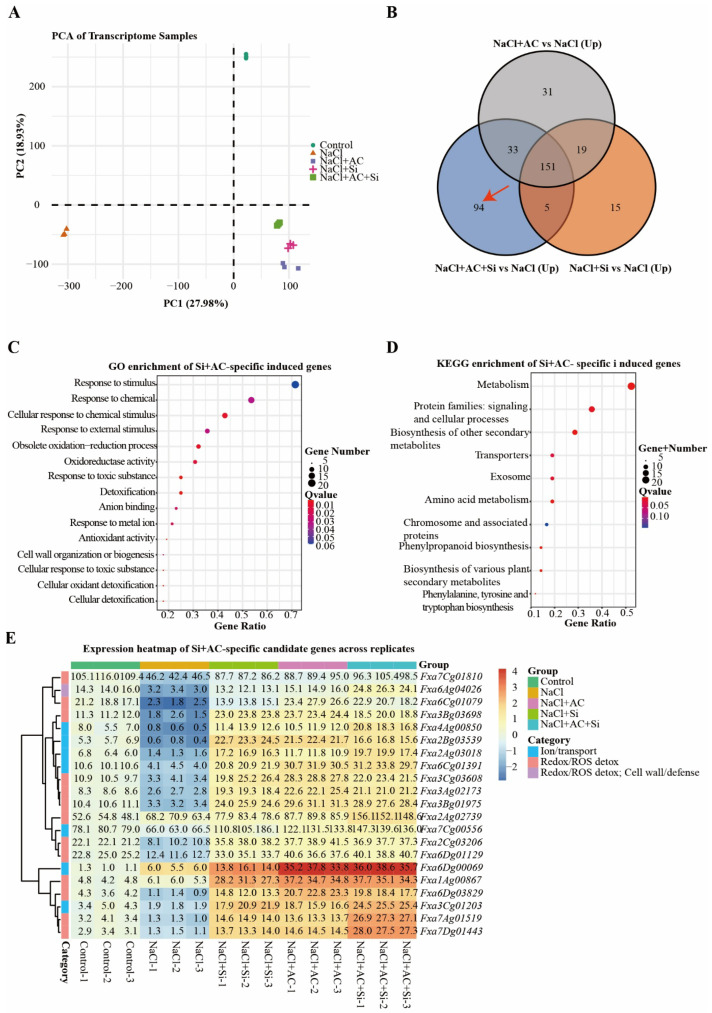
Transcriptome analysis of strawberry plants under different treatments. (**A**) Principal component analysis (PCA) of transcriptome samples. (**B**) Venn diagram of upregulated genes in NaCl + Si vs. NaCl, NaCl + AC vs. NaCl, and NaCl + AC + Si vs. NaCl. The red arrow indicates the overlapping upregulated gene set used for subsequent gene screening. (**C**) Gene Ontology (GO) enrichment of AC + Si-specific induced genes. (**D**) KEGG enrichment of AC + Si-specific induced genes. (**E**) Heatmap showing expression of AC + Si-specific candidate genes across biological replicates. Expression values are shown as log_2_(FPKM + 1) and row-centered by subtracting the mean of the Control group; functional categories are indicated. The curated candidate gene list is provided in Table S7.

RNA sequencing (RNA-seq) data showed clear treatment separation and strong replicate concordance (Figure 5A; Figure S3). Relative to NaCl, AC + Si induced a distinct subset of upregulated genes while sharing a substantial fraction of induced genes with the single-amendment treatments (Figure 5B). Gene Ontology (GO) enrichment of AC + Si-specific induced genes highlighted stimulus-response and redox-related functions (Figure 5C), while Kyoto Encyclopedia of Genes and Genomes (KEGG) enrichment indicated involvement of transport- and metabolism-associated categories (Figure 5D). Importantly, the heatmap of curated AC + Si-responsive candidate genes showed a coherent expression pattern across biological replicates: compared with NaCl, many candidates annotated to ion transport and redox/reactive oxygen species (ROS) detoxification displayed higher expression under AC + Si, accompanied by coordinated changes in genes linked to cell wall/defense-related processes (Figure 5E; Table S7). Collectively, these transcriptomic signatures are consistent with the physiological improvements in ion balance and oxidative stress mitigation observed under the combined treatment.

## 4. Discussion

**Figure 6:**
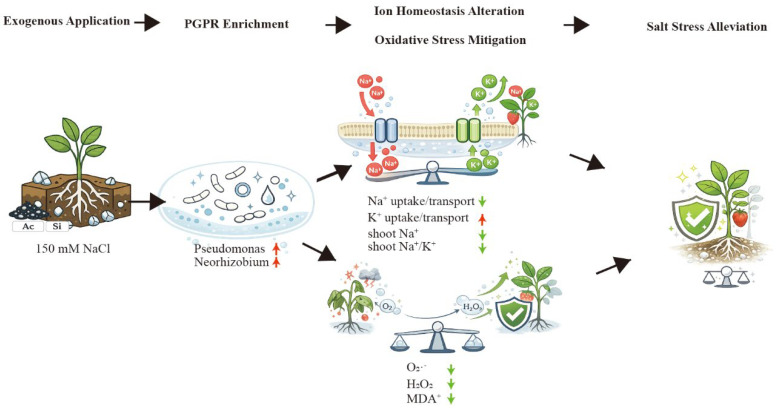
Proposed working model for the combined alleviation of salt stress by AC and Si in strawberry. Conceptual model summarizing that AC + Si reshapes rhizosphere bacterial communities and improves host ion homeostasis and oxidative stress mitigation, collectively alleviating salt stress symptoms.

Salt stress limits strawberry growth through a rapid osmotic phase and a slower ionic phase, the latter marked by Na^+^ over-accumulation, impaired K^+^ nutrition, and secondary oxidative injury [1,5,41]. Across our assays, potassium silicate (Si source) and activated carbon (AC) did not contribute equally: Si alone showed partial protection, AC alone provided little benefit, whereas their co-application produced the most consistent improvements in plant performance, ion balance, redox status, rhizosphere community structure, and stress-responsive transcription (Figure 1, Figure 2, Figure 3, Figure 4 and Figure 5; Figures S1–S3; Tables S1–S7). Collectively, the convergence across independent readouts supports a functionally complementary response that is not readily explained by a simple “single-factor” effect (Figure 6). Importantly, the significance of this study lies in linking phenotype-level performance with coordinated ion/redox physiology, rhizosphere community restructuring, and early transcriptomic signatures under a practical amendment regime, which is especially relevant for salt-sensitive horticultural crops such as strawberry.

### 4.1. Functional Complementarity of Si and AC Under Salinity

Salt stress constrains strawberry performance through both osmotic and ionic components, the latter being closely associated with Na^+^ over-accumulation, K^+^ disequilibrium, and secondary oxidative injury [5,42]. In the present study, Si and AC did not contribute equally to stress mitigation. Si alone provided measurable protection, whereas AC alone exerted comparatively limited effects, while their co-application generated the most consistent improvements across biomass, ion balance, oxidative status, rhizosphere community structure, and stress-responsive transcription (Figure 1, Figure 2, Figure 3, Figure 4 and Figure 5; Figures S1–S3; Table 1 and Tables S1–S7). This overall pattern is more consistent with functional complementarity than with a simple single-factor explanation. Recent literature indicates that silicon-mediated protection under salinity is frequently associated with improved ion regulation, water relations, and oxidative buffering [2,10]. By contrast, carbonaceous amendments are generally context-dependent, with their performance strongly shaped by sorption behavior, physicochemical properties, and plant–soil conditions [15,16]. This interpretation is also compatible with the present study, where AC likely acted primarily by modifying the root-zone context rather than directly conferring stress tolerance. Accordingly, physicochemical characterization of the AC used here is now provided (Table 1; Supplementary File S1), improving material transparency for the present study, although broader comparison across contrasting AC materials remains necessary in future work.

### 4.2. Ion–Redox Coordination as a Physiological Basis of Improved Performance

Maintaining low cytosolic Na^+^ and adequate K^+^ is central to plant performance under salinity because K^+^ is required for enzyme activity, protein synthesis, and membrane potential maintenance [5]. In the present dataset, the combined AC + Si treatment produced the most favorable ion phenotype under NaCl, particularly through reduced shoot Na^+^ accumulation and a lower shoot Na^+^/K^+^ ratio (Figure 3; Tables S2–S4). This pattern is consistent with stronger restriction of Na^+^ toxicity and improved ionic balance, processes that recent silicon-focused studies and reviews have linked to changes in transport regulation, water status, and stress adaptation [2,10]. Because ionic imbalance commonly amplifies ROS generation and lipid peroxidation, the redox response observed here is best interpreted as mechanistically connected to the improved ion status rather than as an isolated outcome [6]. The simultaneous reduction in Na^+^ burden, ROS-related injury, and MDA accumulation (Figure 4; Tables S2–S4) therefore provides a coherent physiological explanation for the better biomass and survival observed under the combined treatment (Figure 1; Tables S1–S4). At the same time, the present data do not exclude additional contributions from changes in rhizosphere ionic strength, nutrient availability, or root exudation patterns, which remain plausible components of the overall response.

### 4.3. Rhizosphere Restructuring as a Plausible Amplifier of Host Stress Buffering

Plants actively shape their rhizosphere microbiome, and treatment-associated shifts in root-associated communities can feed back on nutrient availability, hormone balance, and stress acclimation [18,43]. In the present study, the rhizosphere data showed clear restructuring under AC + Si, especially in comparisons against NaCl and NaCl + AC (Figure 2E–H; Figure S2; Tables S5 and S6). Although 16S rRNA profiles could not resolve function at strain level, the observed compositional shifts were compatible with a microbiome-assisted tolerance model in which a more favorable rhizosphere context supports host ion and redox homeostasis (Figure 2, Figure 3 and Figure 4). This interpretation is consistent with recent syntheses showing that beneficial rhizobacteria can improve plant performance under salinity through nutrient mobilization, hormonal modulation, osmoprotection, ACC deaminase-related stress attenuation, and reinforcement of antioxidant capacity [22,44]. However, these results remain associative rather than causal. Thus, the microbiome findings should be viewed as supportive evidence that complements the physiological data, rather than as direct proof that specific taxa drove the observed stress alleviation. In this context, the weak effect of AC alone is also informative: carbonaceous amendments may alter the rhizosphere environment, but such changes do not necessarily translate into improved host performance unless the plant is simultaneously maintained in a physiologically competent state.

### 4.4. Transcriptome-Supported Mechanistic Context and Agronomic Outlook

The transcriptome data provide an additional layer of support for interpreting the AC + Si response. Rather than standing alone, the enrichment of ion-transport- and redox-related functions becomes most informative when considered together with the physiological evidence for improved Na^+^ handling and reduced oxidative injury (Figure 3 and Figure 4). In this sense, the transcriptome results strengthen the interpretation that the combined treatment influenced both stress perception and downstream acclimation programs in a direction consistent with improved performance under salinity (Figure 5; Figure S3; Table S7) [7]. From an agronomic perspective, amendment-based strategies are attractive because they can be implemented without immediate genetic change, but their practical value will depend on both plant physiology and root-zone context. Key limitations remain: the present study was conducted under controlled conditions; 16S profiles provide compositional rather than functional evidence; and carbonaceous amendment performance is expected to vary with material properties and dosage [16,25]. Future work should therefore combine isolate-based validation or synthetic communities with optimized material characterization and broader testing across soil types and salinity chemistries to establish causality and improve translational relevance [18,22,25,43].

## 5. Conclusions

NaCl stress markedly impaired seedling performance in octoploid cultivated strawberry (*Fragaria* × *ananassa*), coinciding with soil salinization, disrupted Na^+^/K^+^ homeostasis, oxidative injury, and rhizosphere community shifts. Against this stress background, the co-application of potassium silicate and activated carbon provided the most consistent protection, improving growth and survival, enhancing Na^+^/K^+^ balance, and alleviating oxidative damage. This combined treatment was also associated with altered soil physicochemical properties and rhizosphere bacterial assemblages, while transcriptome profiling supported coordinated activation of ion transport- and redox-related pathways. Together, these convergent phenotypic, physiological, microbiome, and transcriptomic readouts indicate that combining silicate and activated carbon offers a practical and mechanistically informed approach to improve strawberry establishment under salinity, although the microbiome evidence is currently associative rather than causal. Future work will validate this strategy under production-like conditions, including salt-affected coastal farmlands impacted by seawater intrusion, and will quantify agronomic outcomes such as seedling establishment, flowering/fruiting performance, yield, and fruit quality. In addition, future studies should address three priorities: (1) characterize activated carbon properties and optimize application rates to improve reproducibility across cultivation substrates and soils, including particle size distribution, ash content, and pore structure; (2) evaluate robustness across different salinity chemistries and management regimes; and (3) establish functional links between rhizosphere changes and host responses through isolate-based assays, synthetic communities, and complementary meta-omics approaches.

## Figures and Tables

**Table 1:** Physicochemical properties of the activated carbon used in this study.

Parameter	Unit	Value	Method/Source
BET specific surface area	m^2^·g^−1^	950.0082	N_2_ adsorption (BET)
Total pore volume	cm^3^·g^−1^	0.414170	Single-point total pore volume
Micropore volume	cm^3^·g^−1^	0.321489	t-plot method
Average pore diameter	nm	1.7439	4 V/A by BET
pH	–	8.3	100 g·L^−1^ aqueous suspension, 25 °C
Ash content	%	3.82	GB/T 12496.3-1999 [27]

Note: BET, Brunauer–Emmett–Teller. Pore structure parameters were obtained from the N_2_ adsorption report; pH and ash content were determined by standard tests. The detailed analytical report is provided as Supplementary File S1.

## Data Availability

The RNA-seq raw sequencing data generated in this study have been deposited in the NCBI Sequence Read Archive (SRA) under BioProject accession PRJNA1424399. The rhizosphere 16S rRNA amplicon sequencing raw reads have been deposited in the NCBI SRA under BioProject accession PRJNA1424400.

## Supplementary Materials

The following supporting information can be downloaded at: https://www.mdpi.com/article/10.3390/plants15081154/s1, Figure S1. Experimental design and treatment scheme. Overview of treatment groups, salinity regime (60 → 100 → 150 mM NaCl), sampling points (including 6 h transcriptome sampling), and downstream measurements performed in this study; Figure S2. Rhizosphere bacterial alpha diversity and genus-level composition under different treatments. (A) Chao1 richness index. (B) Shannon diversity index. (C) Genus-level composition (top 20 genera; mean relative abundance across biological replicates). Different letters indicate significant differences among treatments (one-way ANOVA followed by Tukey’s HSD test, *p* < 0.05; *n* = 3); Figure S3. RNA-seq quality control and sample correlation analysis. Sample-to-sample distance heatmap based on variance-stabilizing transformation (VST), showing clustering among biological replicates across treatments (*n* = 3); Figure S4. Preliminary NaCl dose–response phenotyping used to justify the target salinity level. Runner-propagated production seedlings (cv. ‘*Jiandebailu*’) were exposed to a NaCl gradient (0, 25, 50, 100, 150, and 200 mM). Representative plants were photographed after 14 d of treatment. Visible salt-injury symptoms became apparent at 150 mM NaCl, including leaf-margin chlorosis/necrosis, dehydration, and leaf curling, supporting the use of 150 mM NaCl as a severe-salinity level in the main experiment; Table S1. Raw data used for plotting physiological traits (replicate-level) and soil measurements; Table S2. Summary statistics (mean, SD, SE, n) for physiological traits and soil measurements (based on replicate-level data); Table S3. Percent change relative to NaCl treatment (based on group means). Positive indicates increase; negative indicates reduction; Table S4. Two-way ANOVA (Si × AC) under salinity (NaCl background); Table S5. Sequencing statistics of 16S rRNA amplicon libraries; Table S6. ANCOM-BC2-identified significantly differential genera across treatments; Table S7. Si + AC-specific induced functional candidate genes with annotations and functional categories; Supplementary File S1. Full analytical report for physicochemical characterization of the activated carbon used in this study.
