# Supplementary material for: Silicon Combined with Activated Carbon Enhances Salt Tolerance in Strawberry (Fragaria × ananassa) by Reinforcing Ion–Redox Homeostasis and Reshaping the Rhizosphere Microbiome
Source: Plants (Basel). 2026 Apr 9;15(8):1154. doi: 10.3390/plants15081154 (PMC13119609; doi:10.3390/plants15081154)
Supplement: Supplementary file 1 [file plants-15-01154-s001.zip › Supplementary File S1.pdf]

Shanghai Yingxinke Testing Technology Co., Ltd.

Test Report

Report:	260305152688,260305143685		
Test purpose /application	conventional testing	Sample Batch	/
Client	Zhejiang Academy of Agricultural Sciences	Address of the Entrusting Party	/
Sample Status	完好 Good Condition	Sample Source	Commissioned sample submission
Test Project	BET Analyzer, Standard test		
Test Standard	Please refer to next page(s).		
Test Results	This report only provides the measured values. See the summary page of test results in this report for details.		
Remarks	/		
Reviewer	郭浩宇	Signer	何君涵
Issued Date	2026.03.18		

Test Result

Sample State： Good Condition Sample Source： Commissioned sample submission

No.	Test item	Test Result	number of samples
1	BET Analyzer	Please refer to next page(s).	1
2	Standard test	Please refer to next page(s).	1

未经允许严禁编辑

Test Result：

TEST ONE ： BET Analyzer

Sample:	2026030902-QUAN
Operator:	
Submitter:	
File:	D:\xyl\52688\1.SMP

Started:	2026/3/9 21:50:32	Analysis adsorptive:	N2
Completed:	2026/3/10 10:24:23	Analysis bath temp.:	77.350 K
Report time:	2026/3/10 12:31:23	Thermal correction:	Yes
Sample mass:	0.2826 g	Ambient free space:	27.7256 cm^3 Measured
Analysis free space:	82.8911 cm^3	Equilibration interval:	10 s
Low pressure dose:	15.0000 cm^3/g STP	Sample density:	1.000 g/cm^3
Automatic degas:	No		

Summary Report

Surface Area

Single point surface area at p/p° = 0.050445739:	937.9172 m^2/g
BET Surface Area:	950.0082 m^2/g
Langmuir Surface Area:	1,005.1862 m^2/g
t-Plot Micropore Area:	824.2482 m^2/g
t-Plot external surface area:	125.7601 m^2/g
BJH Adsorption cumulative surface area of pores between 1.0000 nm and 300.0000 nm diameter:	178.0040 m^2/g
BJH Desorption cumulative surface area of pores between 1.0000 nm and 300.0000 nm diameter:	141.1607 m^2/g
D-H Adsorption cumulative surface area of pores between 1.7000 nm and 300.0000 nm diameter:	82.3556 m^2/g
D-H Desorption cumulative surface area of pores between 1.7000 nm and 300.0000 nm diameter:	83.9744 m^2/g

未经允许严禁编辑

Sample: 2026030902-QUAN
Operator:
Submitter:
File: D:\xyl\52688\1.SMP

Started: 2026/3/9 21:50:32
Completed: 2026/3/10 10:24:23
Report time: 2026/3/10 12:31:23
Sample mass: 0.2826 g
Analysis free space: 82.8911 cm³
Low pressure dose: 15.0000 cm³/g STP
Automatic degas: No

Analysis adsorptive: N2
Analysis bath temp.: 77.350 K
Thermal correction: Yes
Ambient free space: 27.7256 cm³ Measured
Equilibration interval: 10 s
Sample density: 1.000 g/cm³

Pore Volume

Single point adsorption total pore volume of pores less than 315.6352 nm diameter at p/p° = 0.993904348: 0.414170 cm³/g

Single point desorption total pore volume of pores less than 40.3122 nm diameter at p/p° = 0.950000000: 0.410735 cm³/g

t-Plot micropore volume: 0.321489 cm³/g

BJH Adsorption cumulative volume of pores between 1.0000 nm and 300.0000 nm diameter: 0.091704 cm³/g

BJH Desorption cumulative volume of pores between 1.0000 nm and 300.0000 nm diameter: 0.091793 cm³/g

D-H Adsorption cumulative volume of pores between 1.7000 nm and 300.0000 nm diameter: 0.068060 cm³/g

D-H Desorption cumulative volume of pores between 1.7000 nm and 300.0000 nm diameter: 0.068696 cm³/g

Pore Size

Adsorption average pore diameter (4V/A by BET): 1.7439 nm

Desorption average pore diameter (4V/A by BET): 1.7294 nm

未经允许严禁编辑

Sample: 2026030902-QUAN
Operator:
Submitter:
File: D:\xyl\52688\1.SMP

Started:	2026/3/9 21:50:32	Analysis adsorptive:	N2
Completed:	2026/3/10 10:24:23	Analysis bath temp.:	77.350 K
Report time:	2026/3/10 12:31:23	Thermal correction:	Yes
Sample mass:	0.2826 g	Ambient free space:	27.7256 cm^3 Measured
Analysis free space:	82.8911 cm^3	Equilibration interval:	10 s
Low pressure dose:	15.0000 cm^3/g STP	Sample density:	1.000 g/cm^3
Automatic degas:	No		

Pore Size

BJH Adsorption average pore diameter (4V/A): 2.0607 nm

BJH Desorption average pore diameter (4V/A): 2.6011 nm

D-H Adsorption average pore diameter (4V/A): 3.3057 nm

D-H Desorption average pore diameter (4V/A): 3.2722 nm

Freundlich

Qm·C: 128.3214 ± 2.5526 cm^3/g STP

m: 8.1874 ± 0.5732

Temkin

q·alpha/Qm: 0.040734 ± 0.000909 kJ/mol·(cm^3/g STP)

A: 27,781.0333 ± 7,160.8486 mmHg

DFT Pore Size

Volume in Pores	<	0.465 nm	:	0.01069 cm^3/g
Total Volume in Pores	<=	317.978 nm	:	0.34276 cm^3/g

未经允许严禁编辑

Sample: 2026030902-QUAN
Operator:
Submitter:
File: D:\xyl\52688\1.SMP

Started:	2026/3/9 21:50:32	Analysis adsorptive:	N_2
Completed:	2026/3/10 10:24:23	Analysis bath temp.:	77.350 K
Report time:	2026/3/10 12:31:23	Thermal correction:	Yes
Sample mass:	0.2826 g	Ambient free space:	27.7256 cm³ Measured
Analysis free space:	82.8911 cm³	Equilibration interval:	10 s
Low pressure dose:	15.0000 cm³/g STP	Sample density:	1.000 g/cm³
Automatic degas:	No		

DFT Pore Size

Area in Pores	>	317.978 nm	:	0.000 m²/g
Total Area in Pores	>=	0.465 nm	:	797.227 m²/g

Nanoparticle Size:

Average Particle Size 6.3157 nm

Horvath-Kawazoe

Maximum pore volume at p/p° = 0.983792634: 0.410962 cm³/g

Median pore width: 0.7003 nm

Dubinin-Astakhov

Micropore surface area: 1,026.5364 m²/g

Limiting micropore volume: 0.392914 cm³/g

MP-Method

Cumulative surface area of pores between
0.22219 nm and 1.98000 nm hydraulic radius: 1,232.8513 m²/g

Cumulative pore volume of pores between
0.22219 nm and 1.98000 nm hydraulic radius: 0.393427 cm³/g

未经允许严禁编辑

Sample: 2026030902-QUAN
Operator:
Submitter:
File: D:\xyl\52688\1.SMP

Started:	2026/3/9 21:50:32
Completed:	2026/3/10 10:24:23
Report time:	2026/3/10 12:31:23
Sample mass:	0.2826 g
Analysis free space:	82.8911 cm³
Low pressure dose:	15.0000 cm³/g STP
Automatic degas:	No
Analysis adsorptive:	N2
Analysis bath temp.:	77.350 K
Thermal correction:	Yes
Ambient free space:	27.7256 cm³ Measured
Equilibration interval:	10 s
Sample density:	1.000 g/cm³

MP-Method

Average pore hydraulic radius (V/A): 0.31912 nm

未经允许严禁编辑

Sample: 2026030902-QUAN
Operator:
Submitter:
File: D:\xyl\52688\1.SMP

Started: 2026/3/9 21:50:32
Completed: 2026/3/10 10:24:23
Report time: 2026/3/10 12:31:24
Sample mass: 0.2826 g
Analysis free space: 82.8911 cm³
Low pressure dose: 15.0000 cm³/g STP
Automatic degas: No

Analysis adsorptive: N2
Analysis bath temp.: 77.350 K
Thermal correction: Yes
Ambient free space: 27.7256 cm³ Measured
Equilibration interval: 10 s
Sample density: 1.000 g/cm³

Isotherm Tabular Report

Relative Pressure (p/p°)	Absolute Pressure (mmHg)	Quantity Adsorbed (cm³/g STP)	Elapsed Time (h:min)	Saturation Pressure (mmHg)
			01:10	774.525391
0.000002777	0.002153	15.0328	01:55	775.391418
0.000001787	0.001385	30.0658	02:14	775.139099
0.000002093	0.001622	45.0998	02:45	774.671814
0.000002803	0.002169	60.1303	03:32	773.824524
0.000004744	0.003666	75.1618	04:20	772.937744
0.000008853	0.006835	90.1868	05:10	771.985596
0.000017465	0.013464	105.2102	05:58	770.940979
0.000036153	0.027861	120.2178	06:35	770.625366
0.000078263	0.060274	135.2026	07:07	770.144592
0.000188931	0.145452	150.1400	07:30	769.869446
0.000511942	0.393994	164.8441	07:51	769.605652
0.001585620	1.219495	179.5246	08:13	769.096863
0.004650953	3.575720	193.3150	08:43	768.814453
0.006384796	4.907729	197.5585	09:02	768.658691
0.006512187	5.005723	197.8594	09:17	768.670105
0.010185869	7.829489	203.9266	09:26	768.661804
0.031064930	23.875200	219.8988	09:34	768.557983
0.050445739	38.776340	226.9333	09:41	768.674255
0.078782220	60.555248	232.9880	09:47	768.641052
0.101770897	78.223389	236.1770	09:52	768.622375

未经允许严禁编辑

Sample: 2026030902-QUAN
Operator:
Submitter:
File: D:\xyl\52688\1.SMP

Started: 2026/3/9 21:50:32
Completed: 2026/3/10 10:24:23
Report time: 2026/3/10 12:31:24
Sample mass: 0.2826 g
Analysis free space: 82.8911 cm³
Low pressure dose: 15.0000 cm³/g STP
Automatic degas: No

Analysis adsorptive: N2
Analysis bath temp.: 77.350 K
Thermal correction: Yes
Ambient free space: 27.7256 cm³ Measured
Equilibration interval: 10 s
Sample density: 1.000 g/cm³

Isotherm Tabular Report

Relative Pressure (p/p°)	Absolute Pressure (mmHg)	Quantity Adsorbed (cm³/g STP)	Elapsed Time (h:min)	Saturation Pressure (mmHg)
0.123484965	94.921379	238.4248	09:57	768.687744
0.144788774	111.289093	240.1441	10:00	768.630676
0.163255926	125.477066	241.3785	10:04	768.591187
0.182123956	139.993240	242.4594	10:07	768.670105
0.201141288	154.618820	243.3993	10:10	768.707520
0.248388886	190.927307	245.3077	10:13	768.662842
0.277760462	213.490601	246.2809	10:16	768.614075
0.303305882	233.136185	247.0434	10:18	768.650391
0.340229974	261.522491	248.0219	10:20	768.663879
0.379203191	291.497131	248.9767	10:22	768.709595
0.419540457	322.470795	249.8537	10:25	768.628601
0.459385012	353.087402	250.6763	10:27	768.608887
0.499390751	383.825775	251.4650	10:28	768.588074
0.539555006	414.684906	252.2197	10:30	768.568359
0.559577521	430.051514	252.6156	10:32	768.528931
0.579864203	445.654449	252.9894	10:33	768.549683
0.599582912	460.849731	253.3718	10:34	768.617188
0.619874885	476.441345	253.7461	10:35	768.608887
0.639759457	491.690247	254.1342	10:36	768.554871
0.659794049	507.124939	254.5375	10:38	768.610962
0.679798666	522.495728	254.9317	10:39	768.603638

未经允许严禁编辑

Sample: 2026030902-QUAN
Operator:
Submitter:
File: D:\xyl\52688\1.SMP

Started: 2026/3/9 21:50:32
Completed: 2026/3/10 10:24:23
Report time: 2026/3/10 12:31:24
Sample mass: 0.2826 g
Analysis free space: 82.8911 cm³
Low pressure dose: 15.0000 cm³/g STP
Automatic degas: No

Analysis adsorptive: N2
Analysis bath temp.: 77.350 K
Thermal correction: Yes
Ambient free space: 27.7256 cm³ Measured
Equilibration interval: 10 s
Sample density: 1.000 g/cm³

Isotherm Tabular Report

Relative Pressure (p/p°)	Absolute Pressure (mmHg)	Quantity Adsorbed (cm³/g STP)	Elapsed Time (h:min)	Saturation Pressure (mmHg)
0.699667894	537.762207	255.3442	10:40	768.596375
0.719776216	553.241333	255.7708	10:42	768.629639
0.739674395	568.532593	256.2003	10:43	768.625488
0.779442301	599.015015	257.0555	10:44	768.517456
0.819116895	629.563477	258.0509	10:46	768.588074
0.849592455	652.939026	258.9290	10:47	768.532043
0.879406537	675.867615	259.9228	10:49	768.549683
0.909262906	698.801453	261.1171	10:51	768.536194
0.939200952	721.833313	262.5562	10:53	768.561096
0.959469194	737.330994	263.7573	10:55	768.478027
0.974587193	748.988281	264.8504	10:56	768.518494
0.983792634	756.031189	265.6851	10:58	768.486328
0.996082424	765.464355	268.2062	11:05	768.474915
0.969258371	744.811462	266.5020	11:07	768.434387
0.944275748	725.500244	265.3172	11:08	768.313965
0.922863744	709.118103	264.6078	11:10	768.388733
0.892905607	686.080078	263.8243	11:11	768.367981
0.862656463	662.830444	263.1257	11:12	768.359680
0.850691254	653.643921	262.7837	11:13	768.367981
0.819866904	629.894775	262.2959	11:14	768.289063
0.762491506	585.828125	261.3696	11:15	768.307739

未经允许严禁编辑

Sample: 2026030902-QUAN
Operator:
Submitter:
File: D:\xyl\52688\1.SMP

Started: 2026/3/9 21:50:32
Completed: 2026/3/10 10:24:23
Report time: 2026/3/10 12:31:24
Sample mass: 0.2826 g
Analysis free space: 82.8911 cm³
Low pressure dose: 15.0000 cm³/g STP
Automatic degas: No

Analysis adsorptive: N2
Analysis bath temp.: 77.350 K
Thermal correction: Yes
Ambient free space: 27.7256 cm³ Measured
Equilibration interval: 10 s
Sample density: 1.000 g/cm³

Isotherm Tabular Report

Relative Pressure (p/p°)	Absolute Pressure (mmHg)	Quantity Adsorbed (cm³/g STP)	Elapsed Time (h:min)	Saturation Pressure (mmHg)
0.740914476	569.239563	260.8040	11:17	768.293213
0.700168103	537.951111	260.0992	11:18	768.317078
0.642633414	493.738892	259.1482	11:20	768.305664
0.620356471	476.654968	258.7575	11:21	768.356567
0.580306465	445.894928	258.1180	11:23	768.378357
0.540749894	415.484772	257.4667	11:24	768.349243
0.500685799	384.689606	256.7303	11:26	768.325378
0.459864023	353.253571	252.1761	11:32	768.169617
0.438395757	336.776428	251.2498	11:34	768.201843
0.403344780	309.817535	250.2127	11:36	768.120850
0.381248750	292.852234	249.6412	11:38	768.139526
0.360605754	276.972687	249.1332	11:40	768.076172
0.340506464	261.558594	248.6289	11:42	768.145752
0.330305371	253.704483	248.3503	11:43	768.090698
0.320241154	245.936020	248.0807	11:44	767.971313
0.300628167	230.893478	247.5399	11:46	768.036743
0.252404211	193.830551	245.9935	11:49	767.937073
0.201701273	154.899536	243.9396	11:53	767.965088
0.161562624	124.060028	241.7535	11:57	767.875793
0.131254771	100.801125	239.5468	12:01	767.980652
0.101954692	78.287376	236.6401	12:05	767.864380

未经允许严禁编辑

Sample: 2026030902-QUAN
Operator:
Submitter:
File: D:\xyl\52688\1.SMP

Started: 2026/3/9 21:50:32
Completed: 2026/3/10 10:24:23
Report time: 2026/3/10 12:31:24
Sample mass: 0.2826 g
Analysis free space: 82.8911 cm³
Low pressure dose: 15.0000 cm³/g STP
Automatic degas: No

Analysis adsorptive: N2
Analysis bath temp.: 77.350 K
Thermal correction: Yes
Ambient free space: 27.7256 cm³ Measured
Equilibration interval: 10 s
Sample density: 1.000 g/cm³

Isotherm Tabular Report

Relative Pressure (p/p°)	Absolute Pressure (mmHg)	Quantity Adsorbed (cm³/g STP)	Elapsed Time (h:min)	Saturation Pressure (mmHg)
0.081431491	62.519291	233.8420	12:10	767.753235

未经允许严禁编辑

Sample: 2026030902-QUAN
Operator:
Submitter:
File: D:\xyl\52688\1.SMP

Started:	2026/3/9 21:50:32	Analysis adsorptive:	N2
Completed:	2026/3/10 10:24:23	Analysis bath temp.:	77.350 K
Report time:	2026/3/10 12:31:24	Thermal correction:	Yes
Sample mass:	0.2826 g	Ambient free space:	27.7256 cm³ Measured
Analysis free space:	82.8911 cm³	Equilibration interval:	10 s
Low pressure dose:	15.0000 cm³/g STP	Sample density:	1.000 g/cm³
Automatic degas:	No		

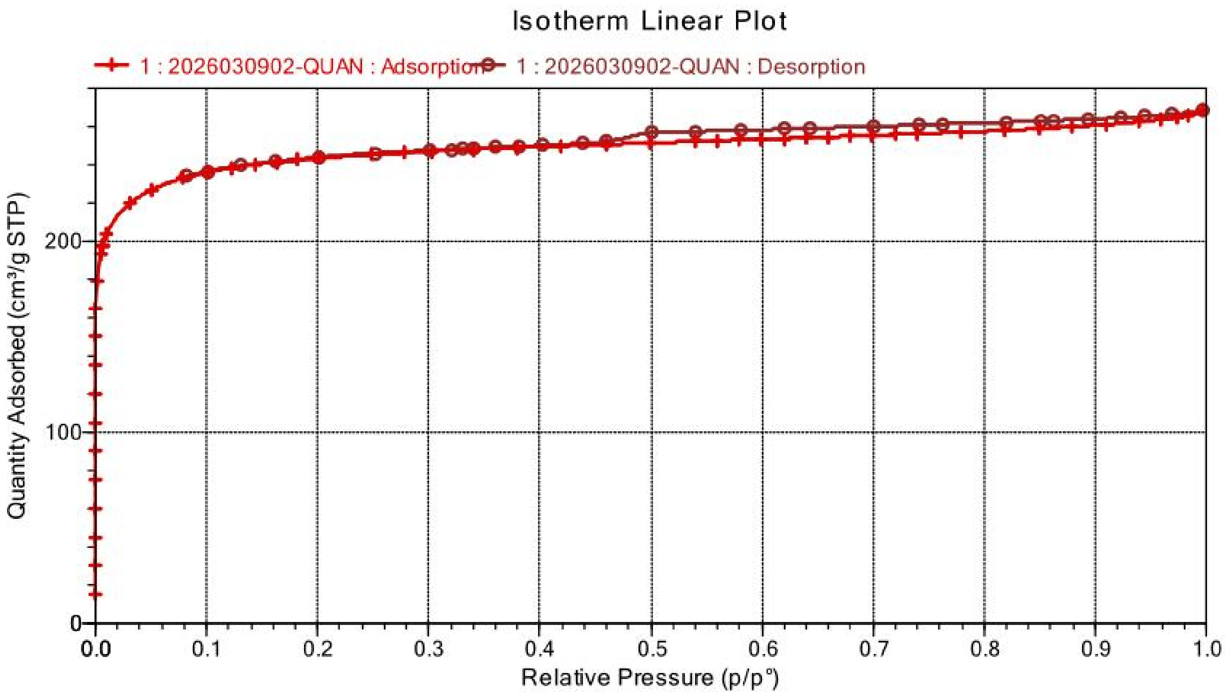

未经允许严禁编辑

Sample: 2026030902-QUAN
Operator:
Submitter:
File: D:\xyl\52688\1.SMP

Started:	2026/3/9 21:50:32	Analysis adsorptive:	N2
Completed:	2026/3/10 10:24:23	Analysis bath temp.:	77.350 K
Report time:	2026/3/10 12:31:24	Thermal correction:	Yes
Sample mass:	0.2826 g	Ambient free space:	27.7256 cm³ Measured
Analysis free space:	82.8911 cm³	Equilibration interval:	10 s
Low pressure dose:	15.0000 cm³/g STP	Sample density:	1.000 g/cm³
Automatic degas:	No		

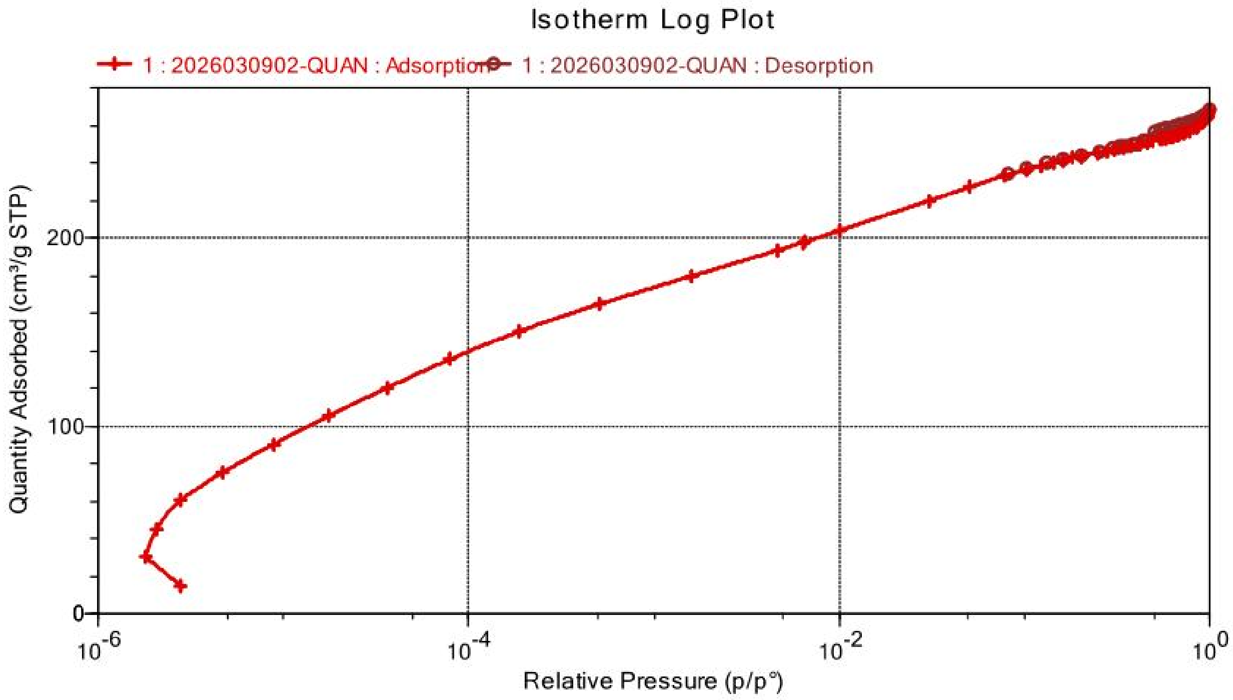

未经允许严禁编辑

Sample: 2026030902-QUAN
Operator:
Submitter:
File: D:\xyl\52688\1.SMP

Started:	2026/3/9 21:50:32	Analysis adsorptive:	N2
Completed:	2026/3/10 10:24:23	Analysis bath temp.:	77.350 K
Report time:	2026/3/10 12:31:24	Thermal correction:	Yes
Sample mass:	0.2826 g	Ambient free space:	27.7256 cm³ Measured
Analysis free space:	82.8911 cm³	Equilibration interval:	10 s
Low pressure dose:	15.0000 cm³/g STP	Sample density:	1.000 g/cm³
Automatic degas:	No		

Isotherm Linear Absolute Plot

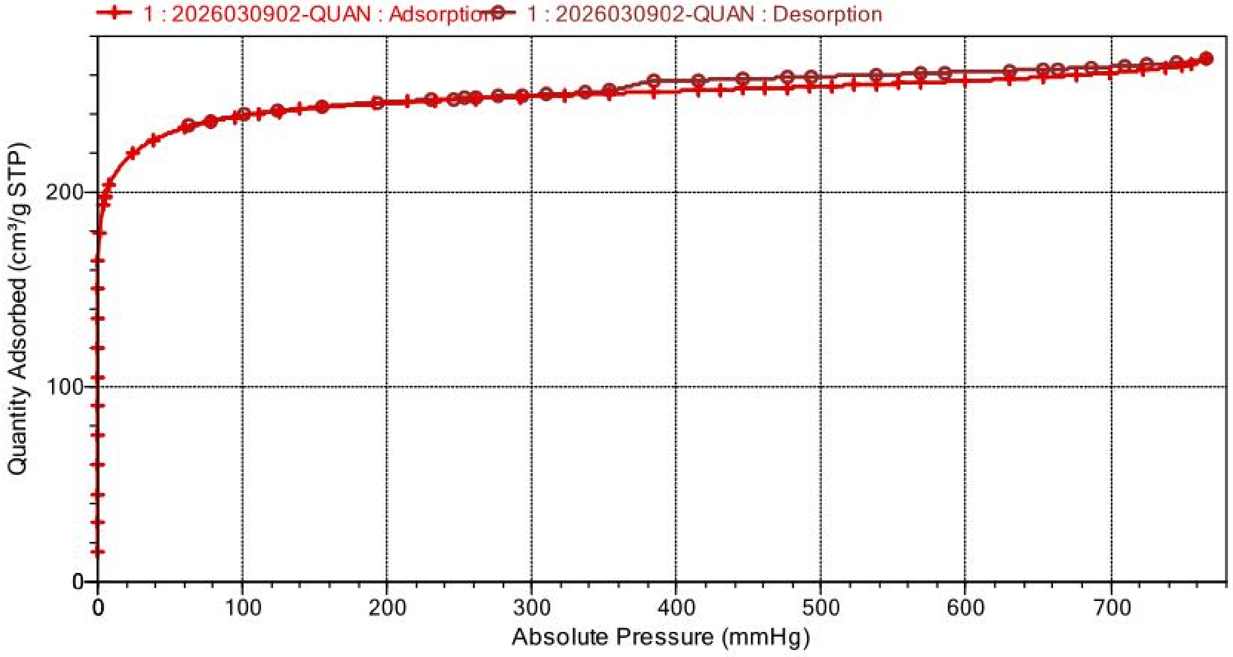

未经允许严禁编辑

Sample: 2026030902-QUAN
Operator:
Submitter:
File: D:\xyl\52688\1.SMP

Started:	2026/3/9 21:50:32	Analysis adsorptive:	N2
Completed:	2026/3/10 10:24:23	Analysis bath temp.:	77.350 K
Report time:	2026/3/10 12:31:24	Thermal correction:	Yes
Sample mass:	0.2826 g	Ambient free space:	27.7256 cm³ Measured
Analysis free space:	82.8911 cm³	Equilibration interval:	10 s
Low pressure dose:	15.0000 cm³/g STP	Sample density:	1.000 g/cm³
Automatic degas:	No		

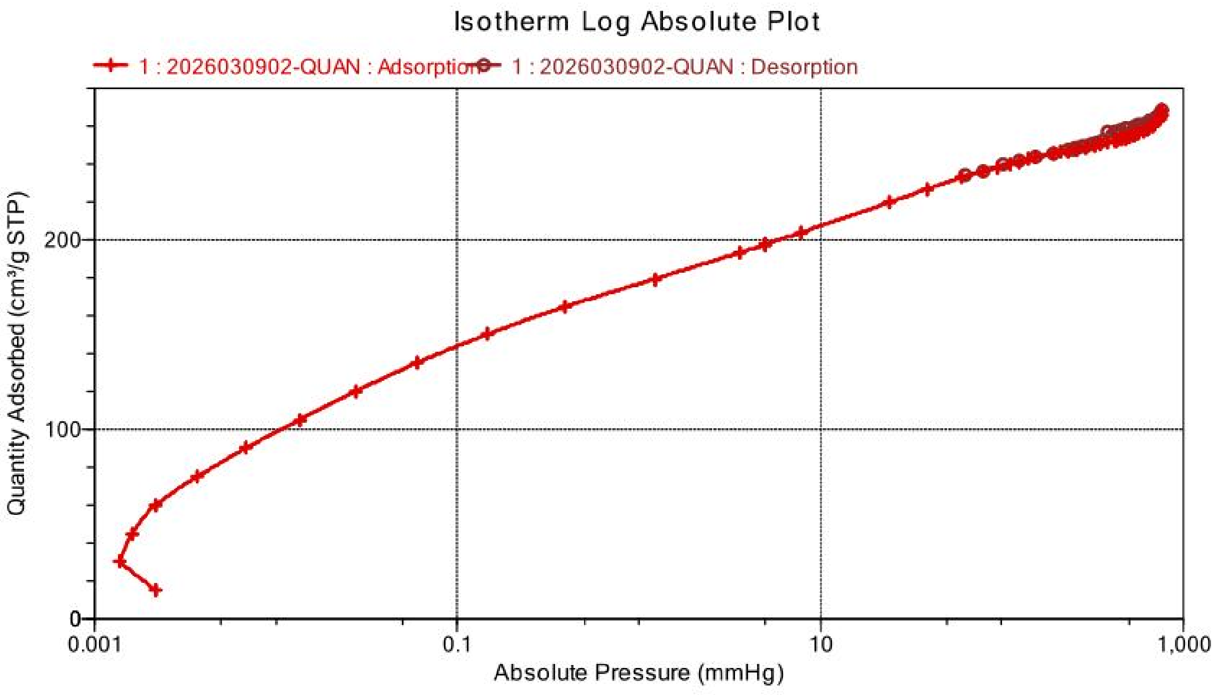

未经允许严禁编辑

Sample: 2026030902-QUAN
Operator:
Submitter:
File: D:\xyl\52688\1.SMP

Started:	2026/3/9 21:50:32	Analysis adsorptive:	N2
Completed:	2026/3/10 10:24:23	Analysis bath temp.:	77.350 K
Report time:	2026/3/10 12:31:24	Thermal correction:	Yes
Sample mass:	0.2826 g	Ambient free space:	27.7256 cm³ Measured
Analysis free space:	82.8911 cm³	Equilibration interval:	10 s
Low pressure dose:	15.0000 cm³/g STP	Sample density:	1.000 g/cm³
Automatic degas:	No		

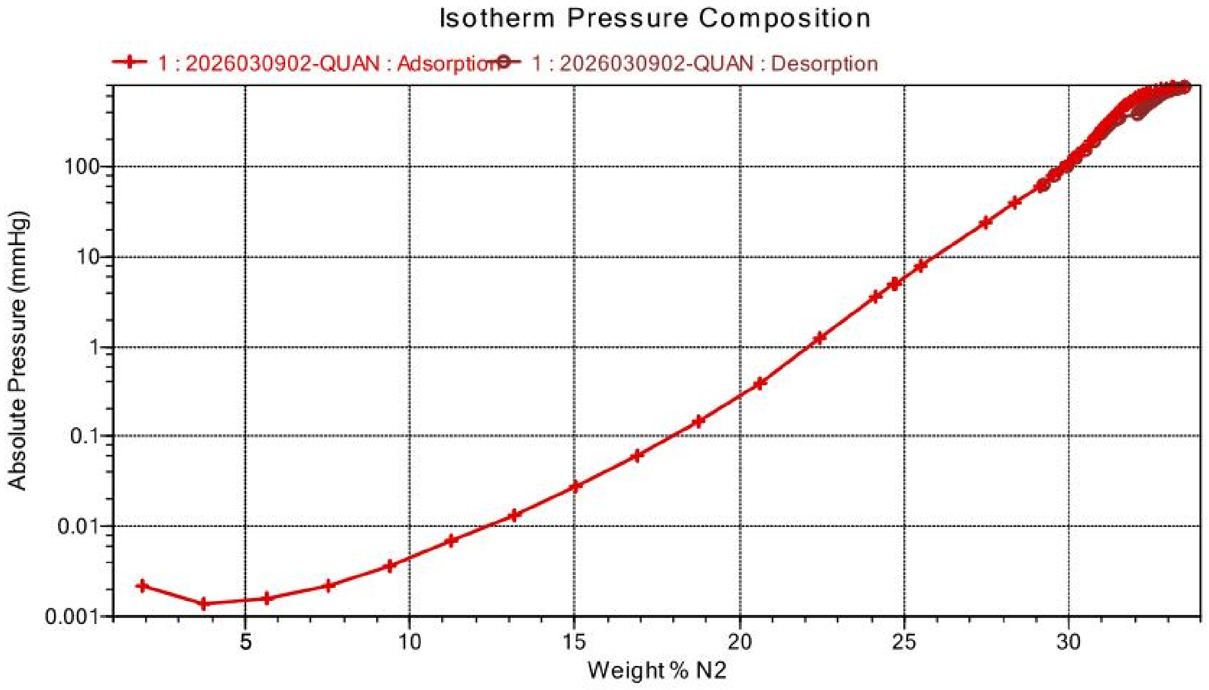

未经允许严禁编辑

Sample: 2026030902-QUAN
Operator:
Submitter:
File: D:\xyl\52688\1.SMP

Started: 2026/3/9 21:50:32
Completed: 2026/3/10 10:24:23
Report time: 2026/3/10 12:31:24
Sample mass: 0.2826 g
Analysis free space: 82.8911 cm³
Low pressure dose: 15.0000 cm³/g STP
Automatic degas: No

Analysis adsorptive: N2
Analysis bath temp.: 77.350 K
Thermal correction: Yes
Ambient free space: 27.7256 cm³ Measured
Equilibration interval: 10 s
Sample density: 1.000 g/cm³

BET Report

BET surface area: 950.0082 ± 1.8446 m²/g
Slope: 0.004578 ± 0.000009 g/cm³ STP
Y-intercept: 0.000003 ± 0.000000 g/cm³ STP
C: 1,371.849635
Qm: 218.2633 cm³/g STP
Correlation coefficient: 0.9999925
Molecular cross-sectional area: 0.1620 nm²

Relative Pressure (p/p°)	Quantity Adsorbed (cm³/g STP)	1/[Q(p°/p - 1)]
0.004650953	193.3150	0.000024
0.006384796	197.5585	0.000033
0.006512187	197.8594	0.000033
0.010185869	203.9266	0.000050
0.031064930	219.8988	0.000146
0.050445739	226.9333	0.000234

未经允许严禁编辑

Sample: 2026030902-QUAN
Operator:
Submitter:
File: D:\xyl\52688\1.SMP

Started: 2026/3/9 21:50:32
Completed: 2026/3/10 10:24:23
Report time: 2026/3/10 12:31:24
Sample mass: 0.2826 g
Analysis free space: 82.8911 cm³
Low pressure dose: 15.0000 cm³/g STP
Automatic degas: No

Analysis adsorptive: N2
Analysis bath temp.: 77.350 K
Thermal correction: Yes
Ambient free space: 27.7256 cm³ Measured
Equilibration interval: 10 s
Sample density: 1.000 g/cm³

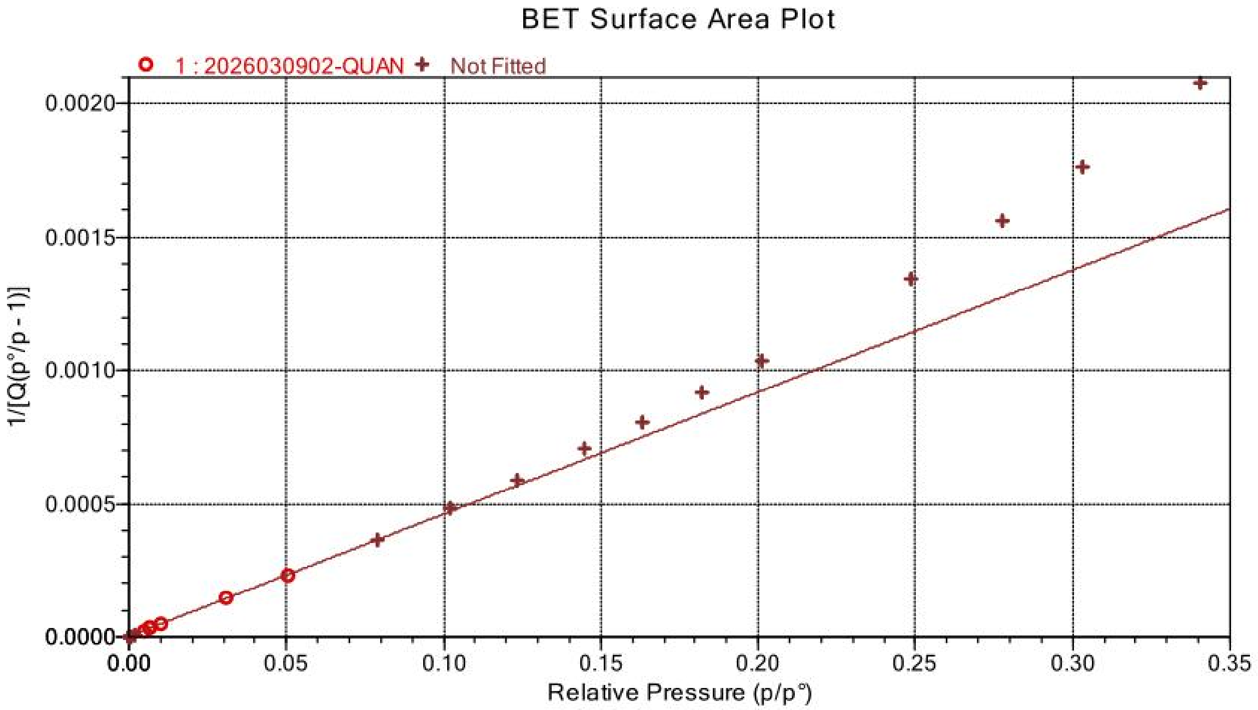

未经允许严禁编辑

Sample: 2026030902-QUAN
Operator:
Submitter:
File: D:\xyl\52688\1.SMP

Started: 2026/3/9 21:50:32
Completed: 2026/3/10 10:24:23
Report time: 2026/3/10 12:31:24
Sample mass: 0.2826 g
Analysis free space: 82.8911 cm³
Low pressure dose: 15.0000 cm³/g STP
Automatic degas: No

Analysis adsorptive: N2
Analysis bath temp.: 77.350 K
Thermal correction: Yes
Ambient free space: 27.7256 cm³ Measured
Equilibration interval: 10 s
Sample density: 1.000 g/cm³

Langmuir Report

Langmuir surface area: 1,005.1862 ± 6.8198 m²/g
Slope: 0.004330 ± 0.000029 g/cm³ STP
Y-intercept: 0.001 ± 0.001 mmHg·g/cm³ STP
b: 3.459638 1/mmHg
Qm: 230.9404 cm³/g STP
Correlation coefficient: 0.999609
Molecular cross-sectional area: 0.1620 nm²

Pressure (mmHg)	Quantity Adsorbed (cm³/g STP)	p/Q (mmHg·g/cm³ STP)
0.002153	15.0328	0.000
0.001385	30.0658	0.000
0.001622	45.0998	0.000
0.002169	60.1303	0.000
0.003666	75.1618	0.000
0.006835	90.1868	0.000
0.013464	105.2102	0.000
0.027861	120.2178	0.000
0.060274	135.2026	0.000
0.145452	150.1400	0.001
0.393994	164.8441	0.002
1.219495	179.5246	0.007
3.575720	193.3150	0.018

未经允许严禁编辑

Sample: 2026030902-QUAN
Operator:
Submitter:
File: D:\xyl\52688\1.SMP

Started: 2026/3/9 21:50:32
Completed: 2026/3/10 10:24:23
Report time: 2026/3/10 12:31:24
Sample mass: 0.2826 g
Analysis free space: 82.8911 cm³
Low pressure dose: 15.0000 cm³/g STP
Automatic degas: No

Analysis adsorptive: N2
Analysis bath temp.: 77.350 K
Thermal correction: Yes
Ambient free space: 27.7256 cm³ Measured
Equilibration interval: 10 s
Sample density: 1.000 g/cm³

Pressure (mmHg)	Quantity Adsorbed (cm³/g STP)	p/Q (mmHg·g/cm³ STP)
4.907729	197.5585	0.025
5.005723	197.8594	0.025
7.829489	203.9266	0.038
23.875200	219.8988	0.109
38.776340	226.9333	0.171
60.555248	232.9880	0.260

未经允许严禁编辑

Sample: 2026030902-QUAN
Operator:
Submitter:
File: D:\xyl\52688\1.SMP

Started:	2026/3/9 21:50:32	Analysis adsorptive:	N2
Completed:	2026/3/10 10:24:23	Analysis bath temp.:	77.350 K
Report time:	2026/3/10 12:31:24	Thermal correction:	Yes
Sample mass:	0.2826 g	Ambient free space:	27.7256 cm³ Measured
Analysis free space:	82.8911 cm³	Equilibration interval:	10 s
Low pressure dose:	15.0000 cm³/g STP	Sample density:	1.000 g/cm³
Automatic degas:	No		

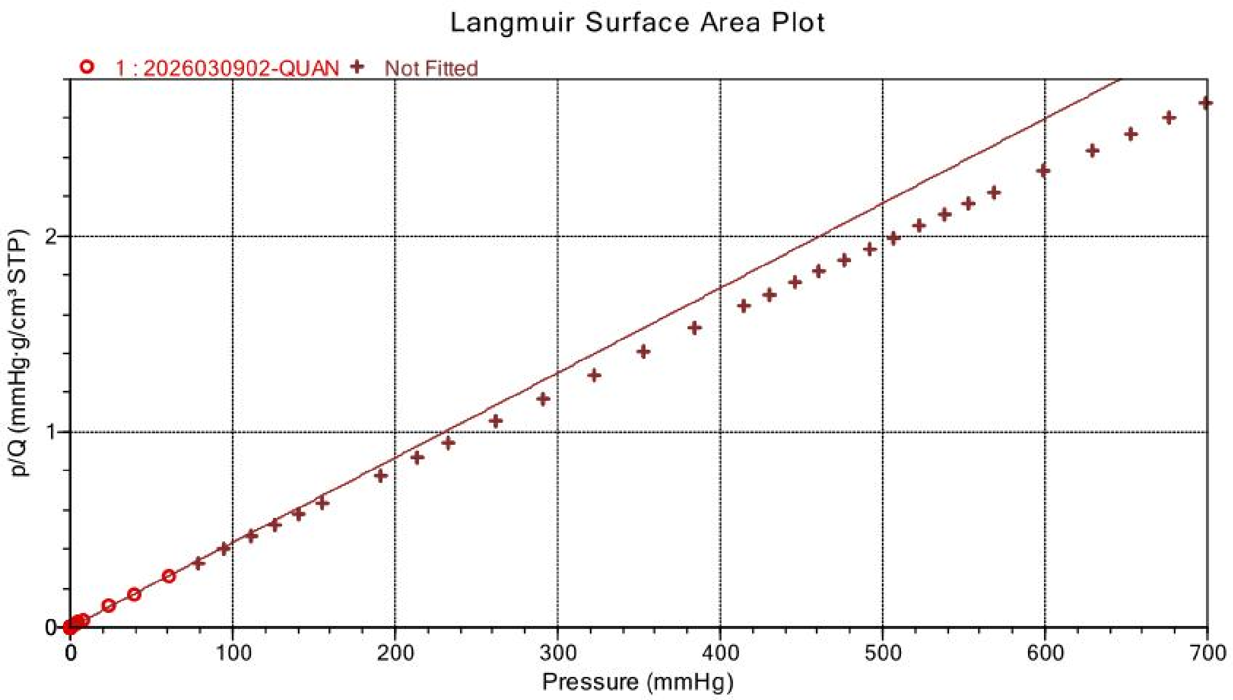

未经允许严禁编辑

Sample: 2026030902-QUAN
Operator:
Submitter:
File: D:\xyl\52688\1.SMP

Started: 2026/3/9 21:50:32
Completed: 2026/3/10 10:24:23
Report time: 2026/3/10 12:31:24
Sample mass: 0.2826 g
Analysis free space: 82.8911 cm³
Low pressure dose: 15.0000 cm³/g STP
Automatic degas: No

Analysis adsorptive: N2
Analysis bath temp.: 77.350 K
Thermal correction: Yes
Ambient free space: 27.7256 cm³ Measured
Equilibration interval: 10 s
Sample density: 1.000 g/cm³

t-Plot Report

Micropore volume: 0.321489 cm³/g
Micropore area: 824.2482 m²/g
External surface area: 125.7601 m²/g
Slope: 81.303374 ± 3.073356 cm³/g·nm STP
Y-intercept: 207.841290 ± 1.307198 cm³/g STP
Correlation coefficient: 0.999286
Surface area correction factor: 1.000
Density conversion factor: 0.0015468
Total surface area (BET): 950.0082 m²/g
Thickness range: 0.40210 to 1.01312 nm
Thickness equation: Harkins and Jura

Thickness Curve

t = [13.99 / (0.034 - log(p/p°))] ^ 0.5

t-Plot Report - Data

Relative Pressure (p/p°)	Statistical Thickness (nm)	Quantity Adsorbed (cm³/g STP)	Fitted
0.000002777	0.15819	15.0328	
0.000001787	0.15555	30.0658	
0.000002093	0.15648	45.0998	
0.000002803	0.15825	60.1303	
0.000004744	0.16159	75.1618	

未经允许严禁编辑

Sample: 2026030902-QUAN
Operator:
Submitter:
File: D:\xyl\52688\1.SMP

Started: 2026/3/9 21:50:32
Completed: 2026/3/10 10:24:23
Report time: 2026/3/10 12:31:24
Sample mass: 0.2826 g
Analysis free space: 82.8911 cm³
Low pressure dose: 15.0000 cm³/g STP
Automatic degas: No

Analysis adsorptive: N2
Analysis bath temp.: 77.350 K
Thermal correction: Yes
Ambient free space: 27.7256 cm³ Measured
Equilibration interval: 10 s
Sample density: 1.000 g/cm³

t-Plot Report - Data

Relative Pressure (p/p°)	Statistical Thickness (nm)	Quantity Adsorbed (cm³/g STP)	Fitted
0.000008853	0.16584	90.1868	
0.000017465	0.17087	105.2102	
0.000036153	0.17680	120.2178	
0.000078263	0.18382	135.2026	
0.000188931	0.19295	150.1400	
0.000511942	0.20513	164.8441	
0.001585620	0.22219	179.5246	
0.004650953	0.24314	193.3150	
0.006384796	0.25053	197.5585	
0.006512187	0.25102	197.8594	
0.010185869	0.26278	203.9266	
0.031064930	0.30123	219.8988	
0.050445739	0.32418	226.9333	
0.078782220	0.35069	232.9880	
0.101770897	0.36919	236.1770	
0.123484965	0.38530	238.4248	
0.144788774	0.40025	240.1441	
0.163255926	0.41276	241.3785	*
0.182123956	0.42525	242.4594	*
0.201141288	0.43762	243.3993	*

未经允许严禁编辑

Sample: 2026030902-QUAN
Operator:
Submitter:
File: D:\xyl\52688\1.SMP

Started: 2026/3/9 21:50:32
Completed: 2026/3/10 10:24:23
Report time: 2026/3/10 12:31:24
Sample mass: 0.2826 g
Analysis free space: 82.8911 cm³
Low pressure dose: 15.0000 cm³/g STP
Automatic degas: No

Analysis adsorptive: N2
Analysis bath temp.: 77.350 K
Thermal correction: Yes
Ambient free space: 27.7256 cm³ Measured
Equilibration interval: 10 s
Sample density: 1.000 g/cm³

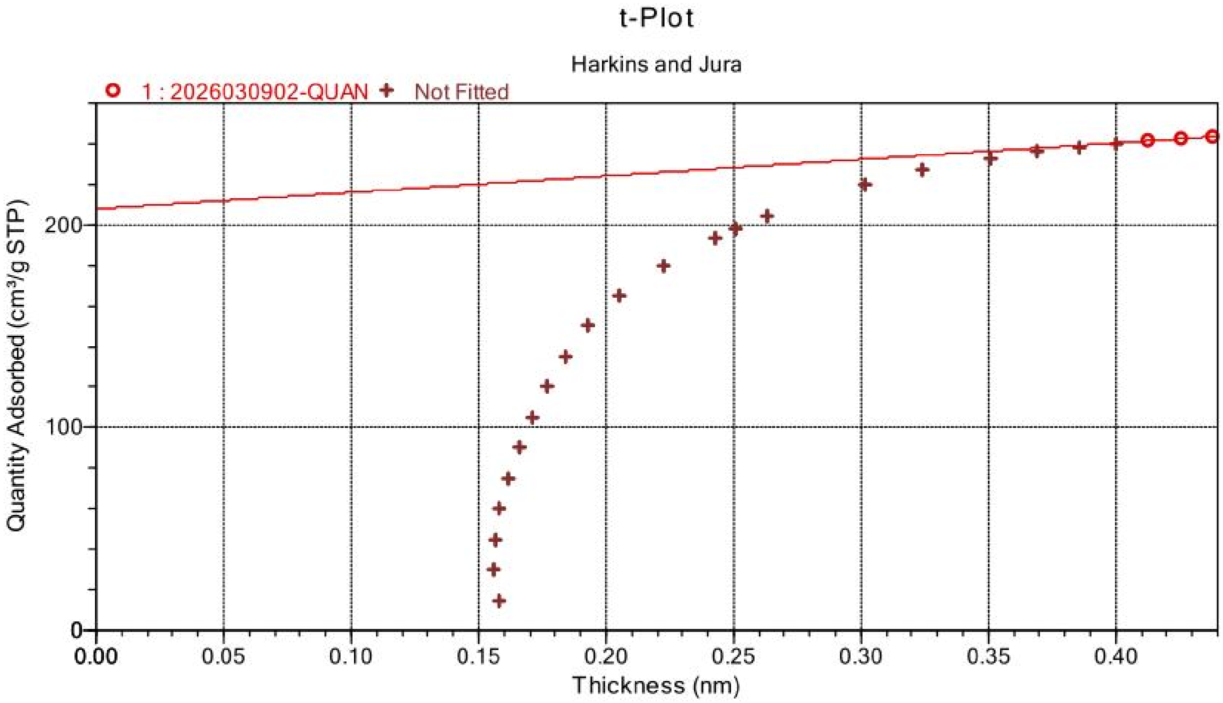

未经允许严禁编辑

Sample: 2026030902-QUAN
Operator:
Submitter:
File: D:\xyl\52688\1.SMP

Started: 2026/3/9 21:50:32
Completed: 2026/3/10 10:24:23
Report time: 2026/3/10 12:31:24
Sample mass: 0.2826 g
Analysis free space: 82.8911 cm³
Low pressure dose: 15.0000 cm³/g STP
Automatic degas: No

Analysis adsorptive: N2
Analysis bath temp.: 77.350 K
Thermal correction: Yes
Ambient free space: 27.7256 cm³ Measured
Equilibration interval: 10 s
Sample density: 1.000 g/cm³

BJH Adsorption Pore Distribution Report

Faas Correction
Harkins and Jura
t = [13.99 / (0.034 - log(p/p°))] ^ 0.5

Diameter range: 1.0000 to 300.0000 nm
Adsorbate property factor: 0.95300 nm
Density conversion factor: 0.0015468
Fraction of pores open at both ends: 0.00

Pore Diameter Range (nm)	Average Diameter (nm)	Incremental Pore Volume (cm³/g)	Cumulative Pore Volume (cm³/g)	Incremental Pore Area (m²/g)	Cumulative Pore Area (m²/g)
489.2 - 120.0	140.2	0.004096	0.004096	0.117	0.117
120.0 - 77.3	89.6	0.001379	0.005475	0.062	0.178
77.3 - 49.1	57.0	0.001860	0.007335	0.131	0.309
49.1 - 33.1	38.0	0.002113	0.009448	0.223	0.532
33.1 - 22.5	25.7	0.002625	0.012073	0.408	0.940
22.5 - 17.0	19.0	0.002236	0.014309	0.471	1.411
17.0 - 13.7	15.0	0.001892	0.016201	0.505	1.916
13.7 - 11.4	12.3	0.001693	0.017894	0.549	2.465
11.4 - 9.3	10.2	0.001944	0.019838	0.766	3.231
9.3 - 7.9	8.5	0.001675	0.021513	0.791	4.021
7.9 - 7.3	7.6	0.000857	0.022370	0.454	4.475
7.3 - 6.8	7.0	0.000858	0.023228	0.490	4.965

未经允许严禁编辑

Sample: 2026030902-QUAN
Operator:
Submitter:
File: D:\xyl\52688\1.SMP

Started: 2026/3/9 21:50:32
Completed: 2026/3/10 10:24:23
Report time: 2026/3/10 12:31:24
Sample mass: 0.2826 g
Analysis free space: 82.8911 cm³
Low pressure dose: 15.0000 cm³/g STP
Automatic degas: No

Analysis adsorptive: N_2
Analysis bath temp.: 77.350 K
Thermal correction: Yes
Ambient free space: 27.7256 cm³ Measured
Equilibration interval: 10 s
Sample density: 1.000 g/cm³

Pore Diameter Range (nm)	Average Diameter (nm)	Incremental Pore Volume (cm³/g)	Cumulative Pore Volume (cm³/g)	Incremental Pore Area (m²/g)	Cumulative Pore Area (m²/g)
6.8 - 6.3	6.5	0.000835	0.024063	0.512	5.477
6.3 - 5.9	6.1	0.000795	0.024858	0.522	5.999
5.9 - 5.5	5.7	0.000826	0.025684	0.578	6.577
5.5 - 5.2	5.4	0.000794	0.026479	0.592	7.168
5.2 - 4.9	5.1	0.000759	0.027238	0.600	7.769
4.9 - 4.6	4.8	0.000793	0.028030	0.664	8.433
4.6 - 4.4	4.5	0.000766	0.028796	0.679	9.112
4.4 - 4.2	4.3	0.000834	0.029630	0.781	9.894
4.2 - 3.7	3.9	0.001571	0.031201	1.598	11.492
3.7 - 3.4	3.5	0.001672	0.032873	1.886	13.378
3.4 - 3.1	3.2	0.001766	0.034639	2.198	15.576
3.1 - 2.8	2.9	0.001899	0.036538	2.608	18.184
2.8 - 2.5	2.6	0.002130	0.038668	3.219	21.403
2.5 - 2.3	2.4	0.002194	0.040862	3.636	25.039
2.3 - 2.2	2.2	0.001746	0.042608	3.120	28.159
2.2 - 2.0	2.1	0.002256	0.044864	4.324	32.483
2.0 - 1.8	1.9	0.004552	0.049417	9.694	42.176
1.8 - 1.7	1.7	0.002285	0.051702	5.301	47.477
1.7 - 1.6	1.6	0.002674	0.054375	6.556	54.033
1.6 - 1.5	1.5	0.003092	0.057467	8.031	62.064
1.5 - 1.4	1.4	0.004363	0.061830	12.112	74.176
1.4 - 1.3	1.3	0.005802	0.067632	17.408	91.584

未经允许严禁编辑

Sample: 2026030902-QUAN
Operator:
Submitter:
File: D:\xyl\52688\1.SMP

Started: 2026/3/9 21:50:32
Completed: 2026/3/10 10:24:23
Report time: 2026/3/10 12:31:24
Sample mass: 0.2826 g
Analysis free space: 82.8911 cm^3
Low pressure dose: 15.0000 cm^3/g STP
Automatic degas: No

Analysis adsorptive: N2
Analysis bath temp.: 77.350 K
Thermal correction: Yes
Ambient free space: 27.7256 cm^3 Measured
Equilibration interval: 10 s
Sample density: 1.000 g/cm^3

Pore Diameter Range (nm)	Average Diameter (nm)	Incremental Pore Volume (cm^3/g)	Cumulative Pore Volume (cm^3/g)	Incremental Pore Area (m^2/g)	Cumulative Pore Area (m^2/g)
1.3 - 1.2	1.2	0.008296	0.075929	27.281	118.865
1.2 - 1.0	1.1	0.015775	0.091704	59.139	178.004

未经允许严禁编辑

Sample: 2026030902-QUAN
Operator:
Submitter:
File: D:\xyl\52688\1.SMP

Started:	2026/3/9 21:50:32
Completed:	2026/3/10 10:24:23
Report time:	2026/3/10 12:31:24
Sample mass:	0.2826 g
Analysis free space:	82.8911 cm³
Low pressure dose:	15.0000 cm³/g STP
Automatic degas:	No
Analysis adsorptive:	N2
Analysis bath temp.:	77.350 K
Thermal correction:	Yes
Ambient free space:	27.7256 cm³ Measured
Equilibration interval:	10 s
Sample density:	1.000 g/cm³

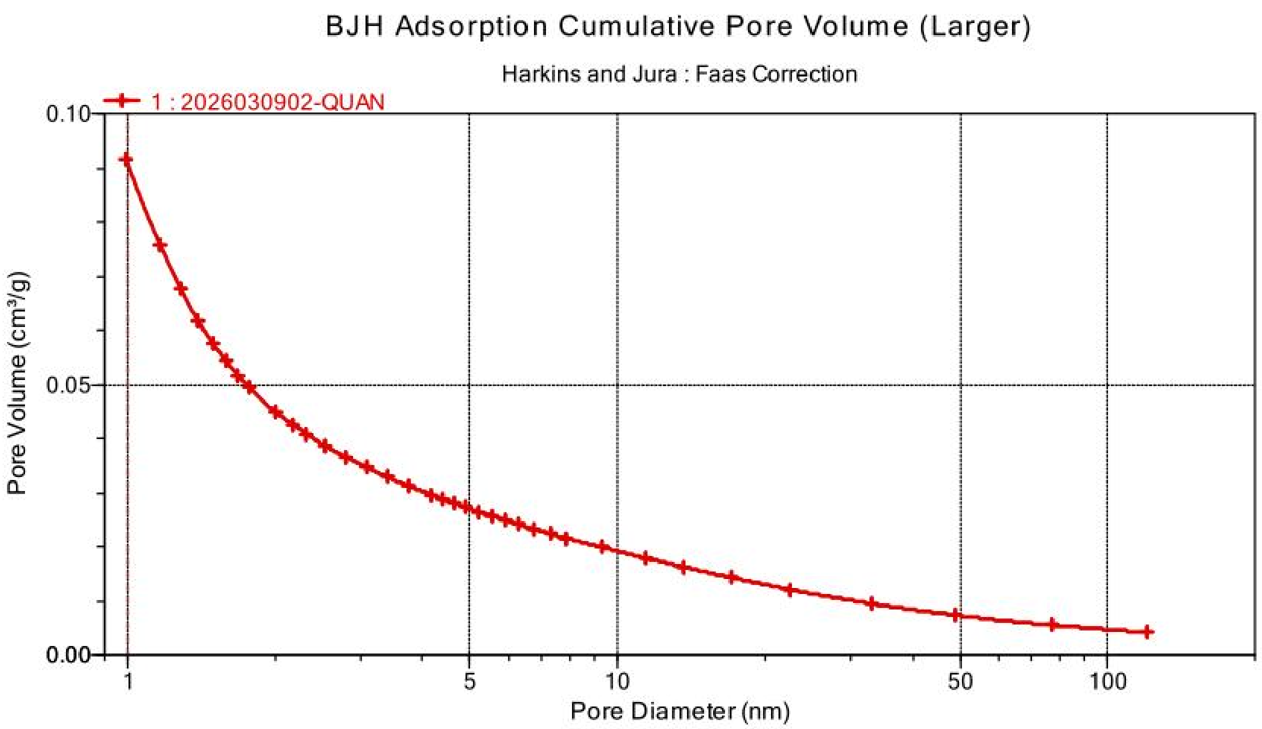

未经允许严禁编辑

Sample: 2026030902-QUAN
Operator:
Submitter:
File: D:\xyl\52688\1.SMP

Started:	2026/3/9 21:50:32	Analysis adsorptive:	N2
Completed:	2026/3/10 10:24:23	Analysis bath temp.:	77.350 K
Report time:	2026/3/10 12:31:24	Thermal correction:	Yes
Sample mass:	0.2826 g	Ambient free space:	27.7256 cm³ Measured
Analysis free space:	82.8911 cm³	Equilibration interval:	10 s
Low pressure dose:	15.0000 cm³/g STP	Sample density:	1.000 g/cm³
Automatic degas:	No		

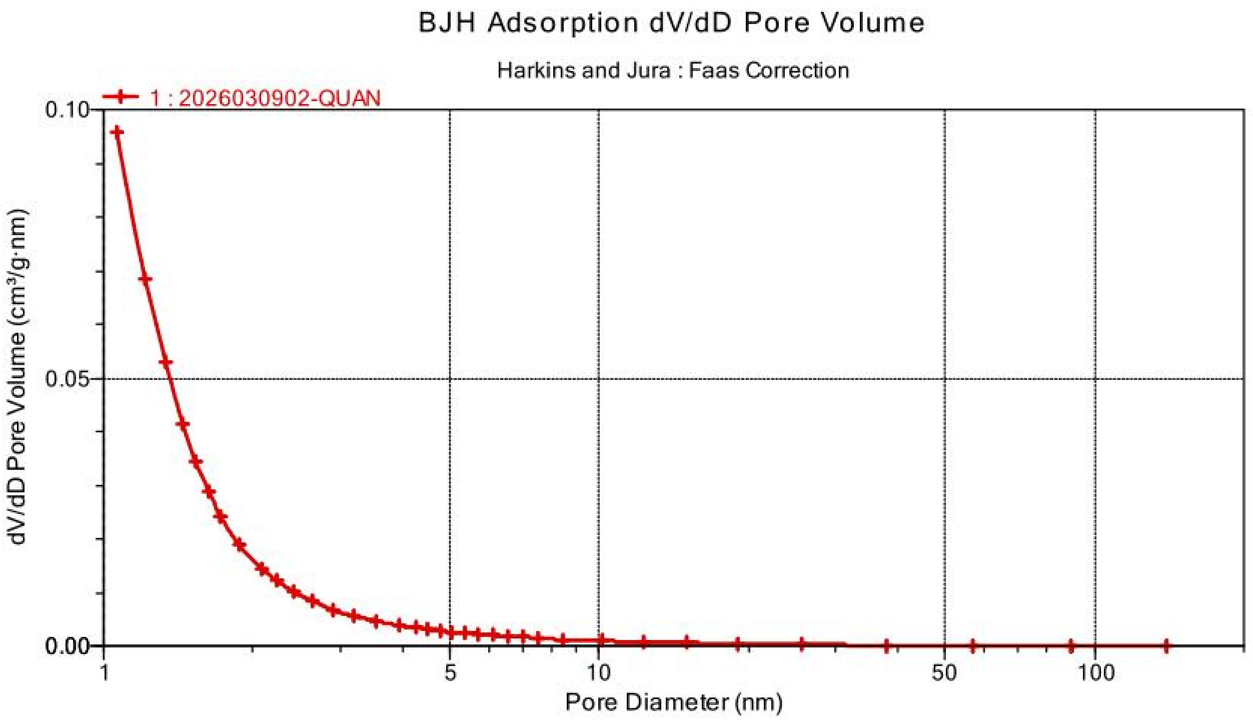

未经允许严禁编辑

Sample: 2026030902-QUAN
Operator:
Submitter:
File: D:\xyl\52688\1.SMP

Started:	2026/3/9 21:50:32	Analysis adsorptive:	N2
Completed:	2026/3/10 10:24:23	Analysis bath temp.:	77.350 K
Report time:	2026/3/10 12:31:24	Thermal correction:	Yes
Sample mass:	0.2826 g	Ambient free space:	27.7256 cm³ Measured
Analysis free space:	82.8911 cm³	Equilibration interval:	10 s
Low pressure dose:	15.0000 cm³/g STP	Sample density:	1.000 g/cm³
Automatic degas:	No		

BJH Adsorption dV/dlog(D) Pore Volume

Harkins and Jura : Faas Correction

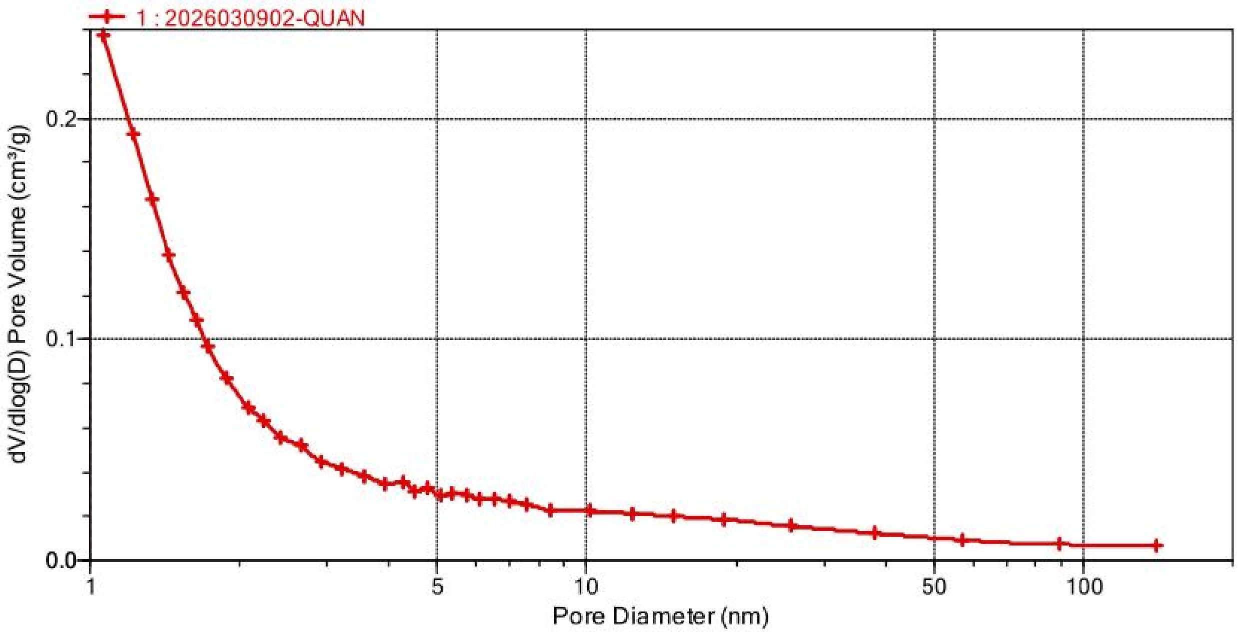

未经允许严禁编辑

Sample: 2026030902-QUAN
Operator:
Submitter:
File: D:\xyl\52688\1.SMP

Started:	2026/3/9 21:50:32	Analysis adsorptive:	N2
Completed:	2026/3/10 10:24:23	Analysis bath temp.:	77.350 K
Report time:	2026/3/10 12:31:24	Thermal correction:	Yes
Sample mass:	0.2826 g	Ambient free space:	27.7256 cm³ Measured
Analysis free space:	82.8911 cm³	Equilibration interval:	10 s
Low pressure dose:	15.0000 cm³/g STP	Sample density:	1.000 g/cm³
Automatic degas:	No		

BJH Adsorption Cumulative Pore Area (Larger)

Harkins and Jura : Faas Correction

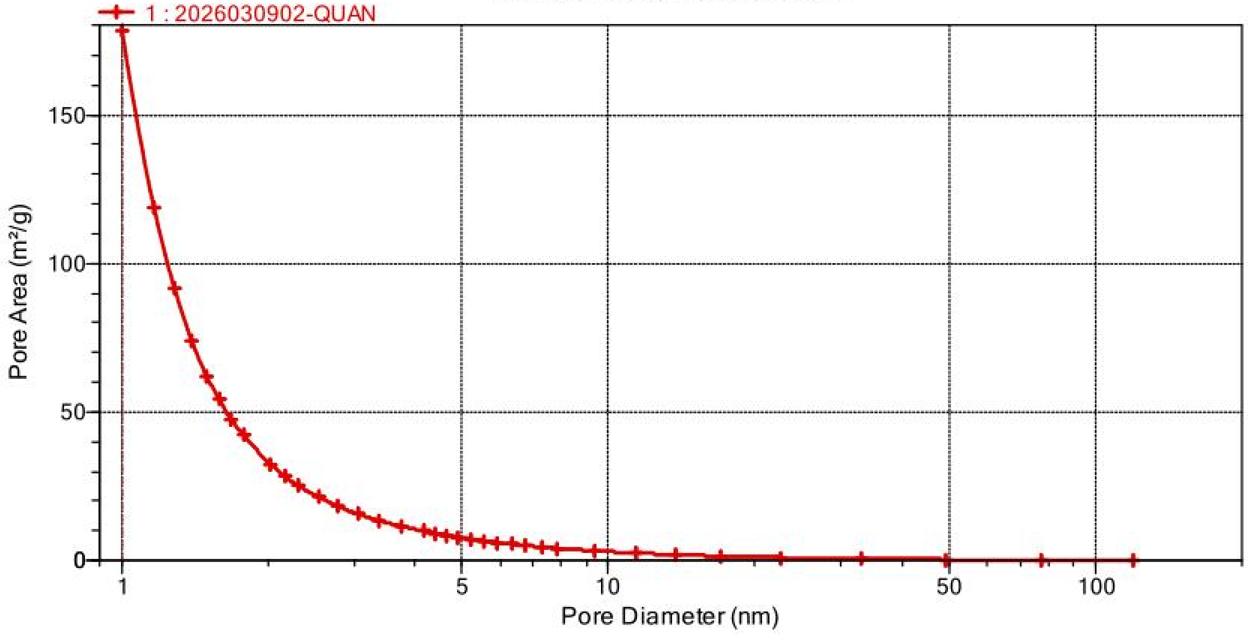

未经允许严禁编辑

Sample: 2026030902-QUAN
Operator:
Submitter:
File: D:\xyl\52688\1.SMP

Started:	2026/3/9 21:50:32	Analysis adsorptive:	N2
Completed:	2026/3/10 10:24:23	Analysis bath temp.:	77.350 K
Report time:	2026/3/10 12:31:24	Thermal correction:	Yes
Sample mass:	0.2826 g	Ambient free space:	27.7256 cm^3 Measured
Analysis free space:	82.8911 cm^3	Equilibration interval:	10 s
Low pressure dose:	15.0000 cm^3/g STP	Sample density:	1.000 g/cm^3
Automatic degas:	No		

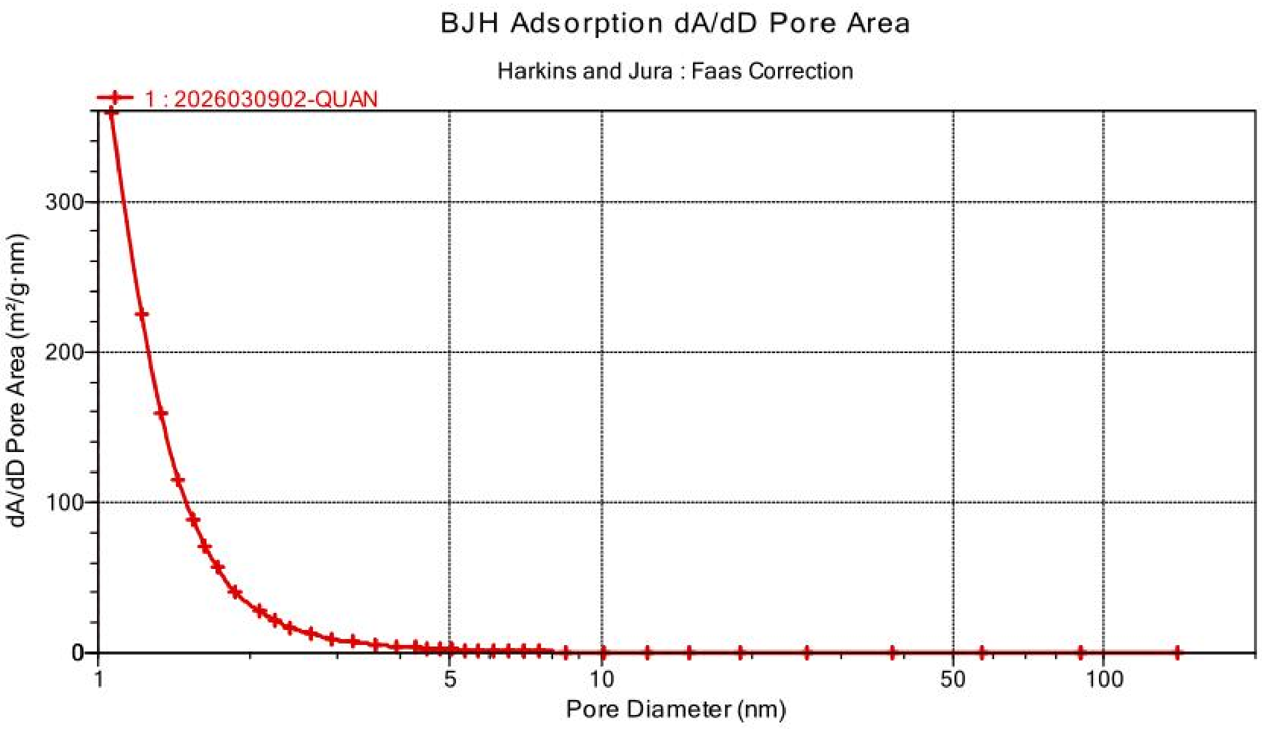

未经允许严禁编辑

Sample: 2026030902-QUAN
Operator:
Submitter:
File: D:\xyl\52688\1.SMP

Started:	2026/3/9 21:50:32	Analysis adsorptive:	N2
Completed:	2026/3/10 10:24:23	Analysis bath temp.:	77.350 K
Report time:	2026/3/10 12:31:24	Thermal correction:	Yes
Sample mass:	0.2826 g	Ambient free space:	27.7256 cm³ Measured
Analysis free space:	82.8911 cm³	Equilibration interval:	10 s
Low pressure dose:	15.0000 cm³/g STP	Sample density:	1.000 g/cm³
Automatic degas:	No		

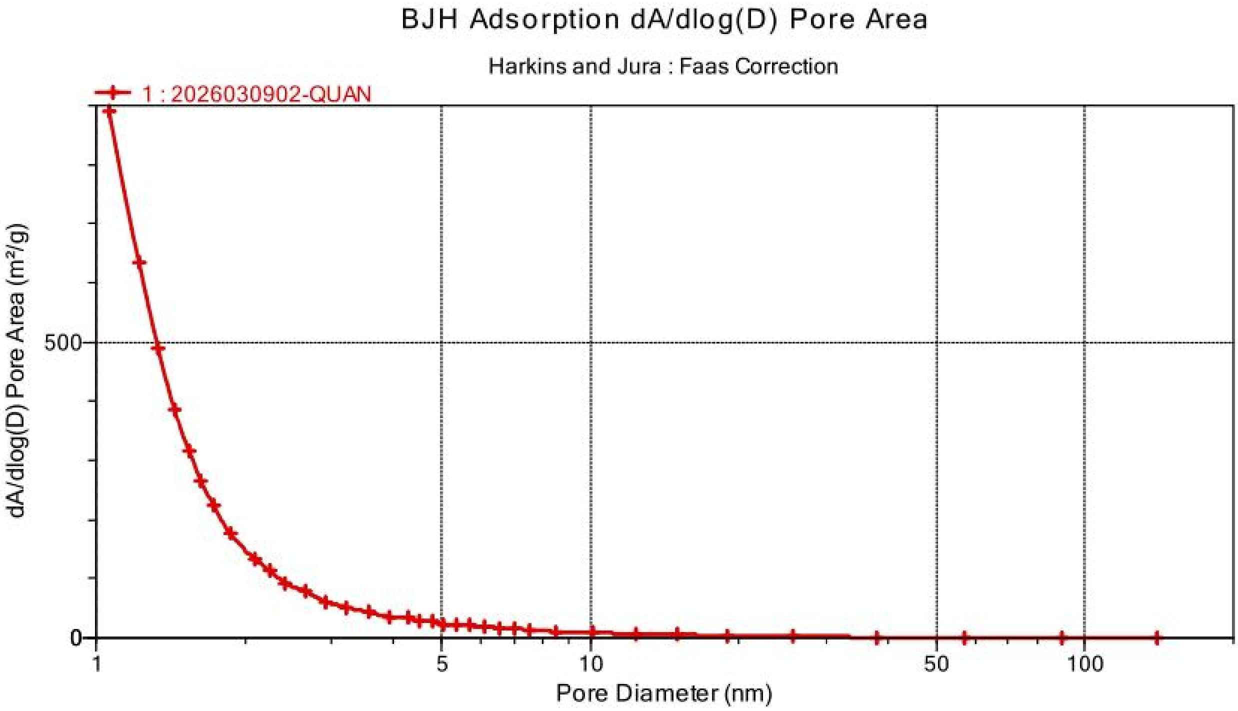

未经允许严禁编辑

Sample: 2026030902-QUAN
Operator:
Submitter:
File: D:\xyl\52688\1.SMP

Started: 2026/3/9 21:50:32
Completed: 2026/3/10 10:24:23
Report time: 2026/3/10 12:31:24
Sample mass: 0.2826 g
Analysis free space: 82.8911 cm³
Low pressure dose: 15.0000 cm³/g STP
Automatic degas: No

Analysis adsorptive: N2
Analysis bath temp.: 77.350 K
Thermal correction: Yes
Ambient free space: 27.7256 cm³ Measured
Equilibration interval: 10 s
Sample density: 1.000 g/cm³

BJH Desorption Pore Distribution Report

Faas Correction
Harkins and Jura
$t = [13.99 / (0.034 - \log(p/p^{\circ}))] ^{0.5}$

Diameter range: 1.0000 to 300.0000 nm
Adsorbate property factor: 0.95300 nm
Density conversion factor: 0.0015468
Fraction of pores open at both ends: 0.00

Pore Diameter Range (nm)	Average Diameter (nm)	Incremental Pore Volume (cm³/g)	Cumulative Pore Volume (cm³/g)	Incremental Pore Area (m²/g)	Cumulative Pore Area (m²/g)
64.5 - 36.3	42.8	0.002128	0.002128	0.199	0.199
36.3 - 26.6	29.9	0.001315	0.003443	0.176	0.375
26.6 - 19.4	21.8	0.001504	0.004946	0.276	0.650
19.4 - 15.3	16.8	0.001385	0.006331	0.329	0.980
15.3 - 14.1	14.6	0.000703	0.007034	0.192	1.171
14.1 - 11.8	12.7	0.000974	0.008007	0.307	1.478
11.8 - 8.9	9.9	0.001962	0.009969	0.790	2.268
8.9 - 8.2	8.5	0.001305	0.011274	0.611	2.879
8.2 - 7.1	7.5	0.001573	0.012848	0.834	3.714
7.1 - 5.9	6.4	0.002187	0.015034	1.377	5.091
5.9 - 5.5	5.7	0.000927	0.015961	0.652	5.743
5.5 - 4.9	5.2	0.001512	0.017473	1.165	6.908

未经允许严禁编辑

Sample: 2026030902-QUAN
Operator:
Submitter:
File: D:\xyl\52688\1.SMP

Started: 2026/3/9 21:50:32
Completed: 2026/3/10 10:24:23
Report time: 2026/3/10 12:31:24
Sample mass: 0.2826 g
Analysis free space: 82.8911 cm^3
Low pressure dose: 15.0000 cm^3/g STP
Automatic degas: No

Analysis adsorptive: N2
Analysis bath temp.: 77.350 K
Thermal correction: Yes
Ambient free space: 27.7256 cm^3 Measured
Equilibration interval: 10 s
Sample density: 1.000 g/cm^3

Pore Diameter Range (nm)	Average Diameter (nm)	Incremental Pore Volume (cm^3/g)	Cumulative Pore Volume (cm^3/g)	Incremental Pore Area (m^2/g)	Cumulative Pore Area (m^2/g)
4.9 - 4.5	4.7	0.001584	0.019057	1.356	8.264
4.5 - 4.0	4.2	0.001878	0.020935	1.774	10.038
4.0 - 3.7	3.8	0.014633	0.035568	15.225	25.263
3.7 - 3.5	3.6	0.002534	0.038102	2.825	28.088
3.5 - 3.2	3.4	0.002459	0.040562	2.926	31.015
3.2 - 3.1	3.2	0.001251	0.041813	1.583	32.598
3.1 - 3.0	3.0	0.001075	0.042888	1.426	34.024
3.0 - 2.8	2.9	0.001086	0.043974	1.505	35.529
2.8 - 2.8	2.8	0.000639	0.044613	0.915	36.444
2.8 - 2.7	2.7	0.000609	0.045221	0.891	37.335
2.7 - 2.6	2.6	0.001244	0.046465	1.883	39.218
2.6 - 2.3	2.4	0.003914	0.050379	6.416	45.634
2.3 - 2.1	2.2	0.005789	0.056168	10.635	56.269
2.1 - 1.9	2.0	0.006839	0.063007	13.991	70.259
1.9 - 1.7	1.8	0.007473	0.070481	16.729	86.988
1.7 - 1.6	1.6	0.010555	0.081036	25.765	112.753
1.6 - 1.5	1.5	0.010758	0.091793	28.407	141.161

未经允许严禁编辑

Sample: 2026030902-QUAN
Operator:
Submitter:
File: D:\xyl\52688\1.SMP

Started:	2026/3/9 21:50:32	Analysis adsorptive:	N2
Completed:	2026/3/10 10:24:23	Analysis bath temp.:	77.350 K
Report time:	2026/3/10 12:31:24	Thermal correction:	Yes
Sample mass:	0.2826 g	Ambient free space:	27.7256 cm³ Measured
Analysis free space:	82.8911 cm³	Equilibration interval:	10 s
Low pressure dose:	15.0000 cm³/g STP	Sample density:	1.000 g/cm³
Automatic degas:	No		

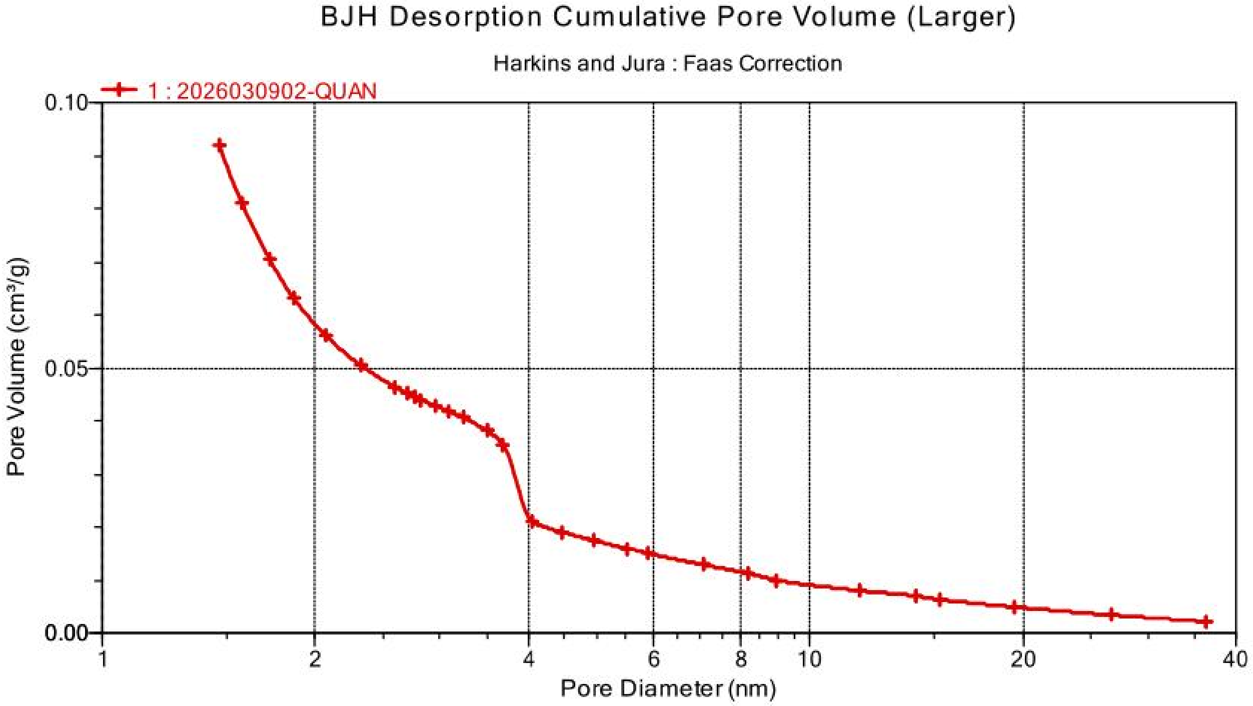

未经允许严禁编辑

Sample: 2026030902-QUAN
Operator:
Submitter:
File: D:\xyl\52688\1.SMP

Started:	2026/3/9 21:50:32	Analysis adsorptive:	N2
Completed:	2026/3/10 10:24:23	Analysis bath temp.:	77.350 K
Report time:	2026/3/10 12:31:24	Thermal correction:	Yes
Sample mass:	0.2826 g	Ambient free space:	27.7256 cm³ Measured
Analysis free space:	82.8911 cm³	Equilibration interval:	10 s
Low pressure dose:	15.0000 cm³/g STP	Sample density:	1.000 g/cm³
Automatic degas:	No		

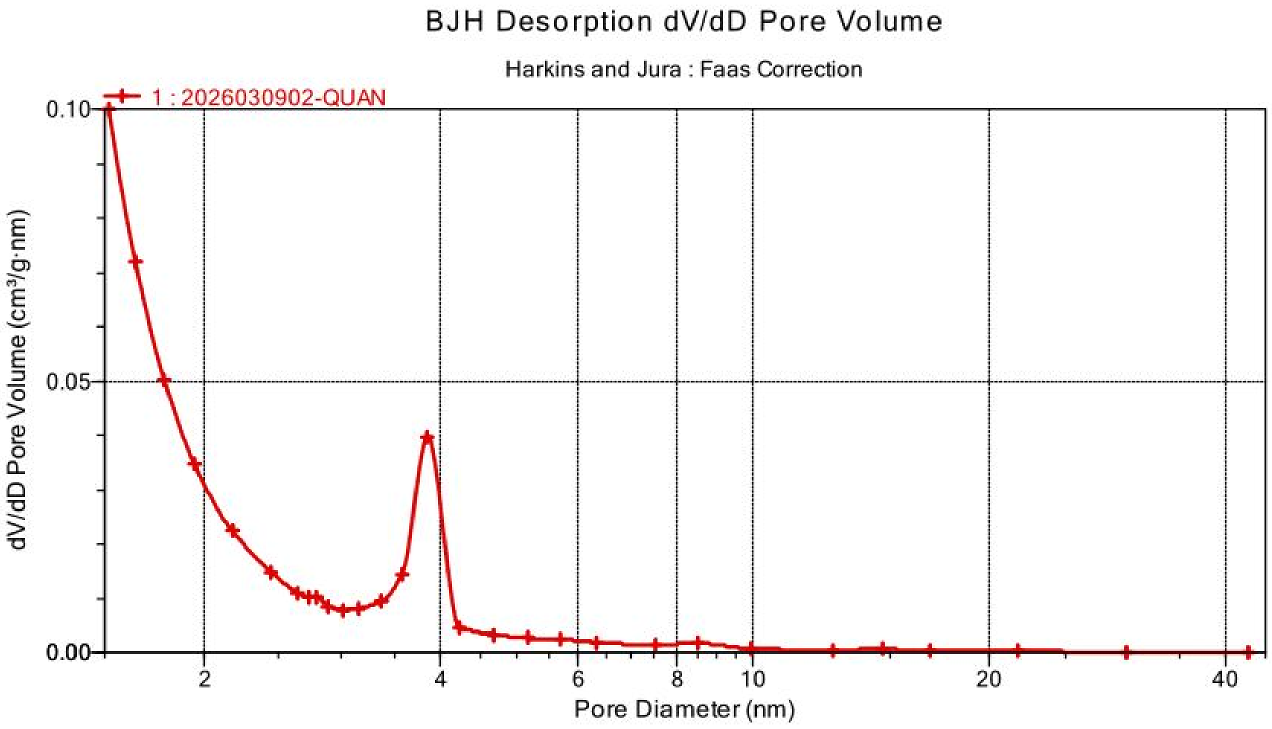

未经允许严禁编辑

Sample: 2026030902-QUAN
Operator:
Submitter:
File: D:\xyl\52688\1.SMP

Started: 2026/3/9 21:50:32
Completed: 2026/3/10 10:24:23
Report time: 2026/3/10 12:31:24
Sample mass: 0.2826 g
Analysis free space: 82.8911 cm³
Low pressure dose: 15.0000 cm³/g STP
Automatic degas: No

Analysis adsorptive: N2
Analysis bath temp.: 77.350 K
Thermal correction: Yes
Ambient free space: 27.7256 cm³ Measured
Equilibration interval: 10 s
Sample density: 1.000 g/cm³

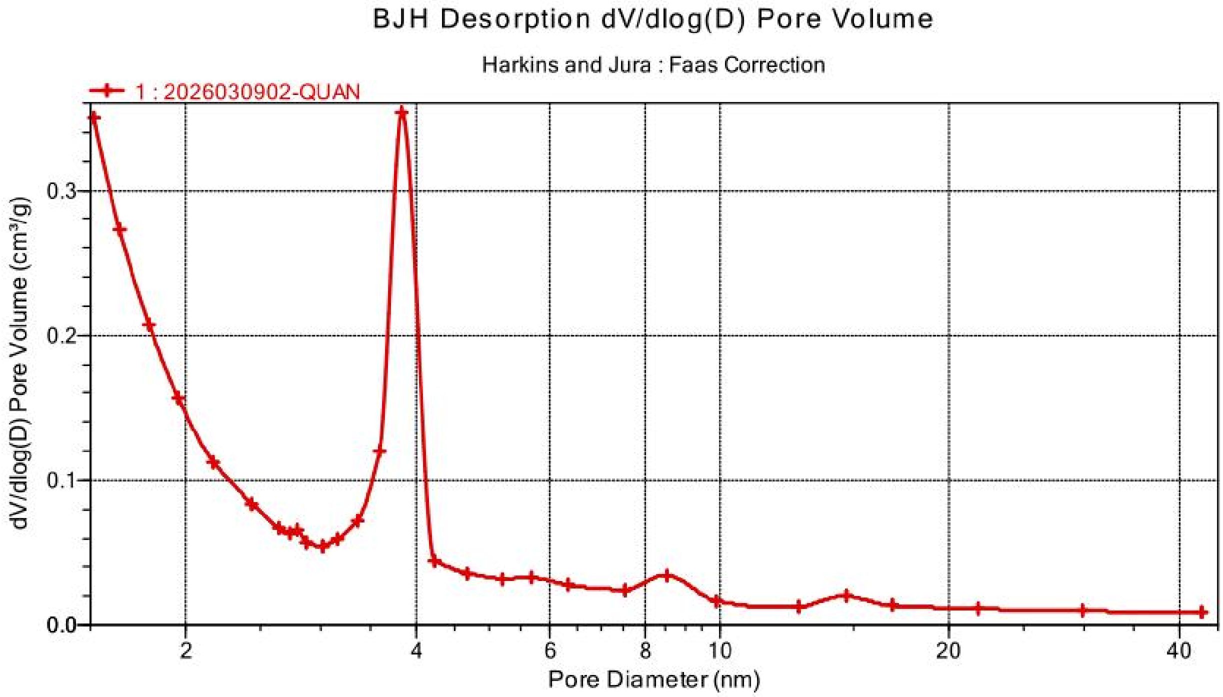

未经允许严禁编辑

Sample: 2026030902-QUAN
Operator:
Submitter:
File: D:\xyl\52688\1.SMP

Started:	2026/3/9 21:50:32	Analysis adsorptive:	N2
Completed:	2026/3/10 10:24:23	Analysis bath temp.:	77.350 K
Report time:	2026/3/10 12:31:24	Thermal correction:	Yes
Sample mass:	0.2826 g	Ambient free space:	27.7256 cm³ Measured
Analysis free space:	82.8911 cm³	Equilibration interval:	10 s
Low pressure dose:	15.0000 cm³/g STP	Sample density:	1.000 g/cm³
Automatic degas:	No		

BJH Desorption Cumulative Pore Area (Larger)

Harkins and Jura : Faas Correction

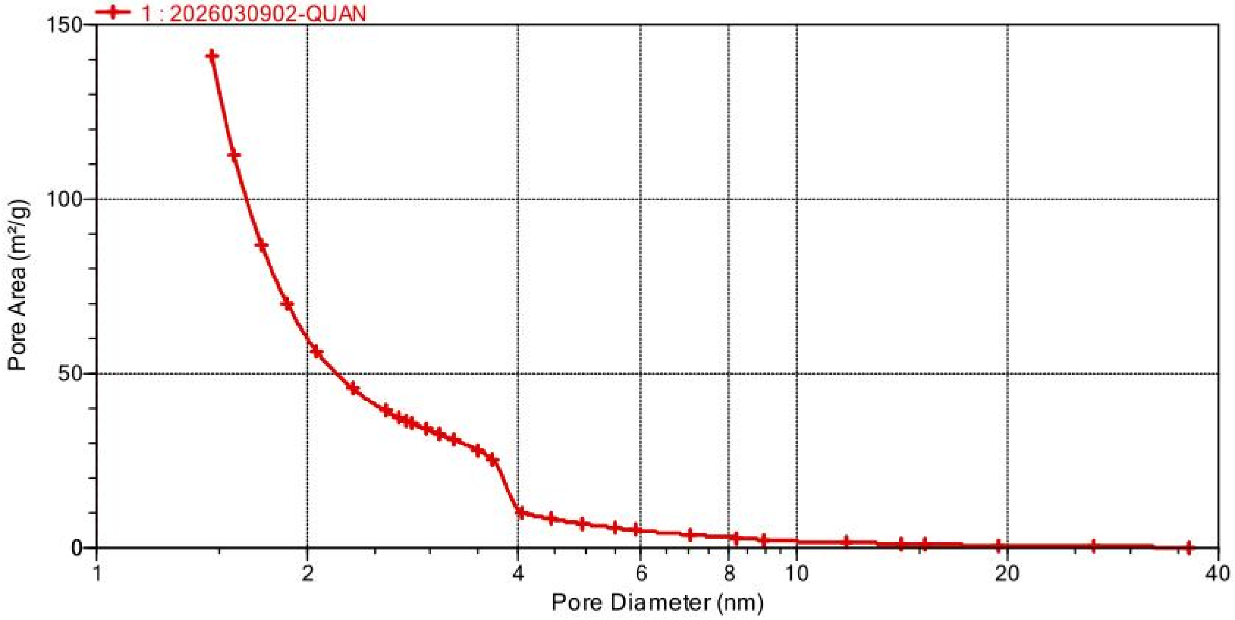

未经允许严禁编辑

Sample: 2026030902-QUAN
Operator:
Submitter:
File: D:\xyl\52688\1.SMP

Started: 2026/3/9 21:50:32
Completed: 2026/3/10 10:24:23
Report time: 2026/3/10 12:31:24
Sample mass: 0.2826 g
Analysis free space: 82.8911 cm³
Low pressure dose: 15.0000 cm³/g STP
Automatic degas: No

Analysis adsorptive: N2
Analysis bath temp.: 77.350 K
Thermal correction: Yes
Ambient free space: 27.7256 cm³ Measured
Equilibration interval: 10 s
Sample density: 1.000 g/cm³

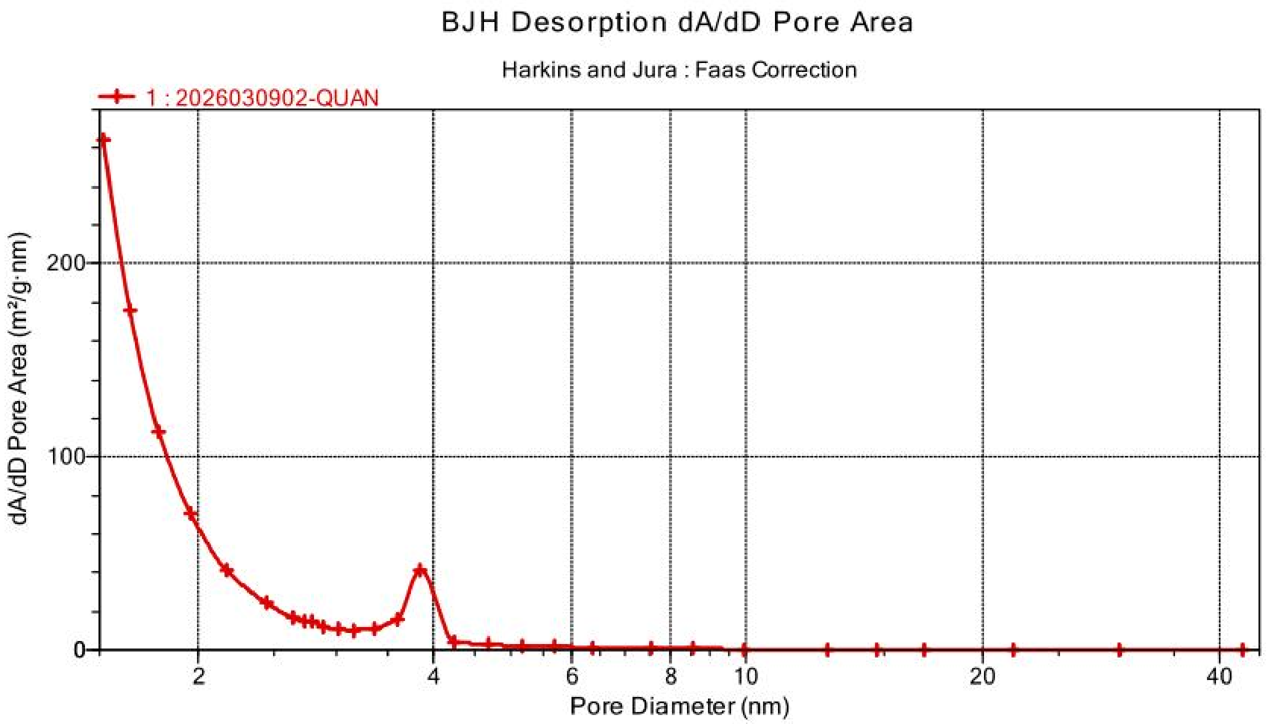

未经允许严禁编辑

Sample: 2026030902-QUAN
Operator:
Submitter:
File: D:\xyl\52688\1.SMP

Started: 2026/3/9 21:50:32
Completed: 2026/3/10 10:24:23
Report time: 2026/3/10 12:31:24
Sample mass: 0.2826 g
Analysis free space: 82.8911 cm³
Low pressure dose: 15.0000 cm³/g STP
Automatic degas: No

Analysis adsorptive: N2
Analysis bath temp.: 77.350 K
Thermal correction: Yes
Ambient free space: 27.7256 cm³ Measured
Equilibration interval: 10 s
Sample density: 1.000 g/cm³

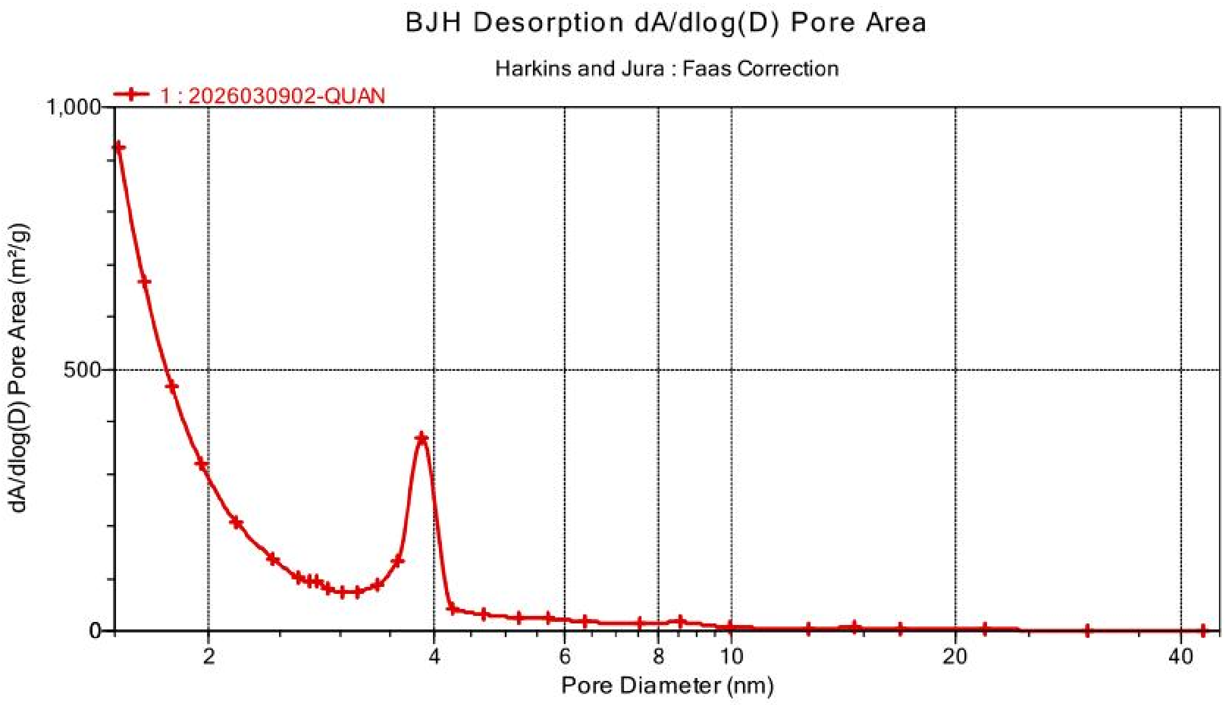

未经允许严禁编辑

Sample: 2026030902-QUAN
Operator:
Submitter:
File: D:\xyl\52688\1.SMP

Started: 2026/3/9 21:50:32
Completed: 2026/3/10 10:24:23
Report time: 2026/3/10 12:31:24
Sample mass: 0.2826 g
Analysis free space: 82.8911 cm³
Low pressure dose: 15.0000 cm³/g STP
Automatic degas: No

Analysis adsorptive: N2
Analysis bath temp.: 77.350 K
Thermal correction: Yes
Ambient free space: 27.7256 cm³ Measured
Equilibration interval: 10 s
Sample density: 1.000 g/cm³

Horvath-Kawazoe Report

Cylinder Pore Geometry (Saito-Foley)

Maximum pore volume: 0.410962 cm³/g
at Relative Pressure: 0.983792634
Median pore width: 0.7003 nm
Relative pressure range: 1e-09 to 0.994845

Diameter of adsorptive molecule: 0.3000 nm
Adsorptive density: 6.710e+14 molecules/cm²
Adsorptive dispersion constant: 7.777e-59
Diameter of sample atom: 0.2760 nm
Sample Density: 1.310e+15 molecules/cm²
Sample dispersion constant: 8.957e-59

Density conversion factor: 0.0015468

Absolute Pressure (mmHg)	Relative Pressure (p/p°)	Quantity Adsorbed (cm³/g STP)	Pore Width (nm)	Cumulative Pore Volume (cm³/g)	Differential Pore Volume (cm³/g·nm)
0.00215	0.000002777	15.03282	0.574	0.0233	0.0405
0.00139	0.000001787	30.06577	0.561	0.0465	-1.7644
0.00162	0.000002093	45.09984	0.566	0.0698	4.9608
0.00217	0.000002803	60.13029	0.575	0.0930	2.6557
0.00367	0.000004744	75.16181	0.591	0.1163	1.3877

未经允许严禁编辑

Sample: 2026030902-QUAN
Operator:
Submitter:
File: D:\xyl\52688\1.SMP

Started: 2026/3/9 21:50:32
Completed: 2026/3/10 10:24:23
Report time: 2026/3/10 12:31:24
Sample mass: 0.2826 g
Analysis free space: 82.8911 cm³
Low pressure dose: 15.0000 cm³/g STP
Automatic degas: No

Analysis adsorptive: N2
Analysis bath temp.: 77.350 K
Thermal correction: Yes
Ambient free space: 27.7256 cm³ Measured
Equilibration interval: 10 s
Sample density: 1.000 g/cm³

Absolute Pressure (mmHg)	Relative Pressure (p/p°)	Quantity Adsorbed (cm³/g STP)	Pore Width (nm)	Cumulative Pore Volume (cm³/g)	Differential Pore Volume (cm³/g·nm)
0.00683	0.000008853	90.18677	0.613	0.1395	1.0849
0.01346	0.000017465	105.21018	0.638	0.1627	0.9079
0.02786	0.000036153	120.21785	0.669	0.1860	0.7579
0.06027	0.000078263	135.20263	0.706	0.2091	0.6258
0.14545	0.000188931	150.14002	0.755	0.2322	0.4687
0.39399	0.000511942	164.84413	0.823	0.2550	0.3342
1.21950	0.001585620	179.52457	0.923	0.2777	0.2270
3.57572	0.004650953	193.31497	1.055	0.2990	0.1626
4.90773	0.006384796	197.55854	1.103	0.3056	0.1352
5.00572	0.006512187	197.85943	1.106	0.3060	0.1467
7.82949	0.010185869	203.92656	1.186	0.3154	0.1175
23.87520	0.031064930	219.89875	1.470	0.3401	0.0871
38.77634	0.050445739	226.93325	1.657	0.3510	0.0582
60.55525	0.078782220	232.98800	1.889	0.3604	0.0404
78.22339	0.101770897	236.17702	2.063	0.3653	0.0282
94.92138	0.123484965	238.42483	2.222	0.3688	0.0219
111.28909	0.144788774	240.14409	2.375	0.3715	0.0175
125.47707	0.163255926	241.37850	2.510	0.3734	0.0141
139.99324	0.182123956	242.45942	2.646	0.3750	0.0122
154.61882	0.201141288	243.39928	2.789	0.3765	0.0102
190.92731	0.248388886	245.30769	3.157	0.3794	0.0080
213.49060	0.277760462	246.28086	3.401	0.3809	0.0062

未经允许严禁编辑

Sample: 2026030902-QUAN
Operator:
Submitter:
File: D:\xyl\52688\1.SMP

Started: 2026/3/9 21:50:32
Completed: 2026/3/10 10:24:23
Report time: 2026/3/10 12:31:24
Sample mass: 0.2826 g
Analysis free space: 82.8911 cm³
Low pressure dose: 15.0000 cm³/g STP
Automatic degas: No

Analysis adsorptive: N2
Analysis bath temp.: 77.350 K
Thermal correction: Yes
Ambient free space: 27.7256 cm³ Measured
Equilibration interval: 10 s
Sample density: 1.000 g/cm³

Absolute Pressure (mmHg)	Relative Pressure (p/p°)	Quantity Adsorbed (cm³/g STP)	Pore Width (nm)	Cumulative Pore Volume (cm³/g)	Differential Pore Volume (cm³/g·nm)
233.13618	0.303305882	247.04339	3.625	0.3821	0.0053
261.52249	0.340229974	248.02189	3.984	0.3836	0.0042
291.49713	0.379203191	248.97672	4.410	0.3851	0.0035
322.47079	0.419540457	249.85366	4.926	0.3865	0.0026
353.08740	0.459385012	250.67625	5.513	0.3877	0.0022
383.82578	0.499390751	251.46504	6.226	0.3890	0.0017
414.68491	0.539555006	252.21969	7.071	0.3901	0.0014
430.05151	0.559577521	252.61559	7.512	0.3907	0.0014
445.65445	0.579864203	252.98940	7.987	0.3913	0.0012
460.84973	0.599582912	253.37182	8.482	0.3919	0.0012
476.44135	0.619874885	253.74611	9.008	0.3925	0.0011
491.69025	0.639759457	254.13419	9.537	0.3931	0.0011
507.12494	0.659794049	254.53754	10.126	0.3937	0.0011
522.49573	0.679798666	254.93166	10.688	0.3943	0.0011
537.76221	0.699667894	255.34415	11.302	0.3950	0.0010
553.24133	0.719776216	255.77078	11.996	0.3956	0.0009
568.53259	0.739674395	256.20030	12.659	0.3963	0.0010
599.01501	0.779442301	257.05549	14.211	0.3976	0.0009
629.56348	0.819116895	258.05093	16.094	0.3992	0.0008
652.93903	0.849592455	258.92897	17.748	0.4005	0.0008
675.86761	0.879406537	259.92281	19.911	0.4020	0.0007
698.80145	0.909262906	261.11706	22.737	0.4039	0.0007

未经允许严禁编辑

Sample: 2026030902-QUAN
Operator:
Submitter:
File: D:\xyl\52688\1.SMP

Started: 2026/3/9 21:50:32
Completed: 2026/3/10 10:24:23
Report time: 2026/3/10 12:31:24
Sample mass: 0.2826 g
Analysis free space: 82.8911 cm^3
Low pressure dose: 15.0000 cm^3/g STP
Automatic degas: No

Analysis adsorptive: N_2
Analysis bath temp.: 77.350 K
Thermal correction: Yes
Ambient free space: 27.7256 cm^3 Measured
Equilibration interval: 10 s
Sample density: 1.000 g/cm^3

Absolute Pressure (mmHg)	Relative Pressure (p/p°)	Quantity Adsorbed (cm^3/g STP)	Pore Width (nm)	Cumulative Pore Volume (cm^3/g)	Differential Pore Volume ($cm^3/g·nm$)
721.83331	0.939200952	262.55620	26.879	0.4061	0.0005
737.33099	0.959469194	263.75734	31.496	0.4080	0.0004
748.98828	0.974587193	264.85037	37.214	0.4097	0.0003
756.03119	0.983792634	265.68505	43.588	0.4110	0.0002

未经允许严禁编辑

Sample: 2026030902-QUAN
Operator:
Submitter:
File: D:\xyl\52688\1.SMP

Started: 2026/3/9 21:50:32
Completed: 2026/3/10 10:24:23
Report time: 2026/3/10 12:31:24
Sample mass: 0.2826 g
Analysis free space: 82.8911 cm³
Low pressure dose: 15.0000 cm³/g STP
Automatic degas: No

Analysis adsorptive: N2
Analysis bath temp.: 77.350 K
Thermal correction: Yes
Ambient free space: 27.7256 cm³ Measured
Equilibration interval: 10 s
Sample density: 1.000 g/cm³

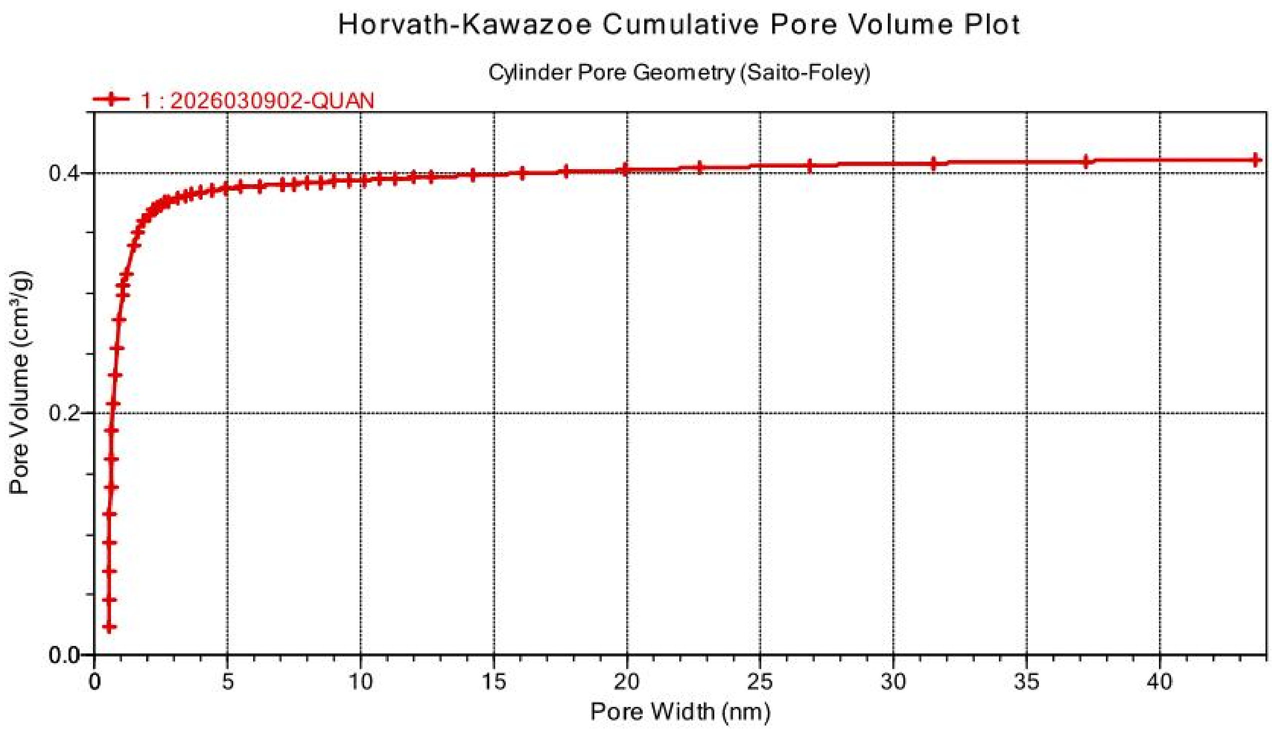

未经允许严禁编辑

Sample: 2026030902-QUAN
Operator:
Submitter:
File: D:\xyl\52688\1.SMP

Started:	2026/3/9 21:50:32	Analysis adsorptive:	N2
Completed:	2026/3/10 10:24:23	Analysis bath temp.:	77.350 K
Report time:	2026/3/10 12:31:24	Thermal correction:	Yes
Sample mass:	0.2826 g	Ambient free space:	27.7256 cm³ Measured
Analysis free space:	82.8911 cm³	Equilibration interval:	10 s
Low pressure dose:	15.0000 cm³/g STP	Sample density:	1.000 g/cm³
Automatic degas:	No		

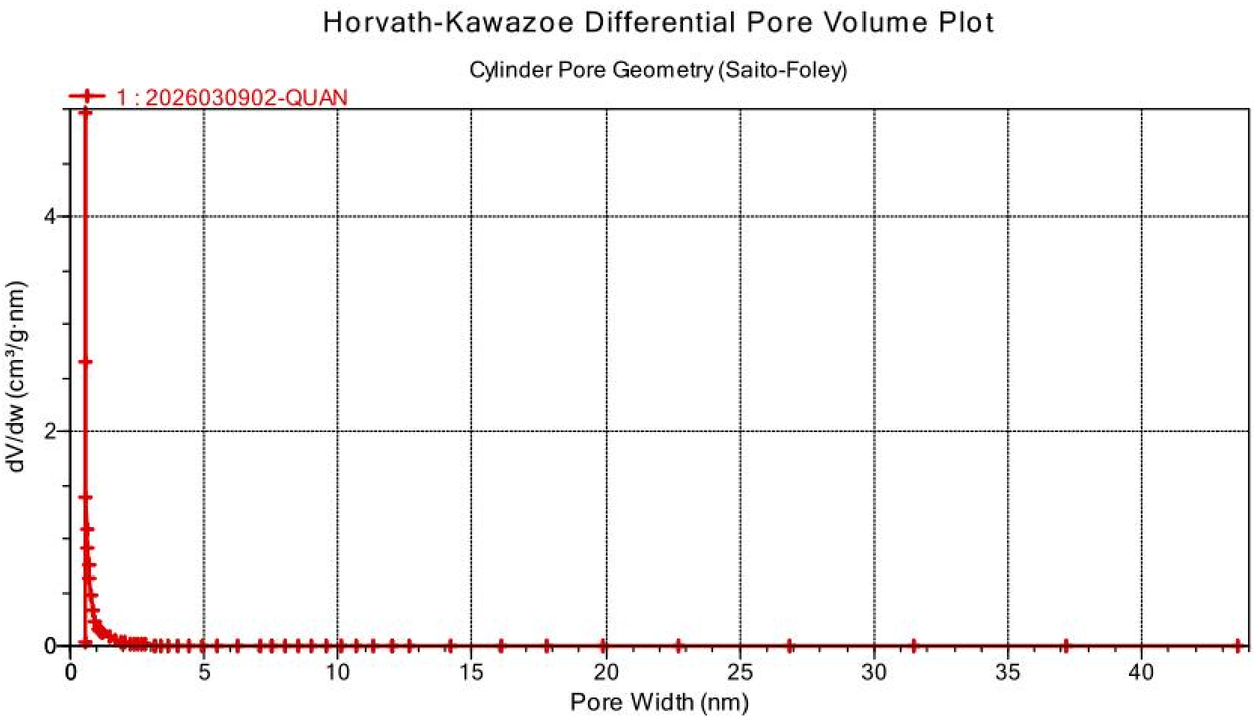

未经允许严禁编辑

Sample: 2026030902-QUAN
Operator:
Submitter:
File: D:\xyl\52688\1.SMP

Started:	2026/3/9 21:50:32	Analysis adsorptive:	N2
Completed:	2026/3/10 10:24:23	Analysis bath temp.:	77.350 K
Report time:	2026/3/10 12:31:24	Thermal correction:	Yes
Sample mass:	0.2826 g	Ambient free space:	27.7256 cm³ Measured
Analysis free space:	82.8911 cm³	Equilibration interval:	10 s
Low pressure dose:	15.0000 cm³/g STP	Sample density:	1.000 g/cm³
Automatic degas:	No		

Porosity Distribution by Invalid
Model: N2 - DFT Model
Method: Non-negative Regularization: 0.02300
Standard Deviation of Fit: 1.80494 cm³/g STP

Volume in Pores	<	0.465 nm	:	0.01069 cm³/g
Total Volume in Pores	<=	317.978 nm	:	0.34276 cm³/g
Area in Pores	>	317.978 nm	:	0.000 m²/g
Total Area in Pores	>=	0.465 nm	:	797.227 m²/g

Pore Table

Pore Width (nm)	Cumulative Pore Volume (cm³/g)	Incremental Pore Volume (cm³/g)	Cumulative Pore Area (m²/g)	Incremental Pore Area (m²/g)
0.465	0.02259	0.01189	51.193	51.193
0.500	0.03929	0.01670	117.955	66.762
0.536	0.06068	0.02138	197.728	79.773
0.590	0.08682	0.02615	286.398	88.670
0.643	0.11631	0.02948	378.057	91.659
0.679	0.14586	0.02955	465.085	87.028
0.733	0.17338	0.02752	540.200	75.116
0.804	0.19698	0.02360	598.899	58.699
0.858	0.21485	0.01787	640.560	41.661
0.929	0.22748	0.01264	667.756	27.196
1.001	0.23614	0.00866	685.066	17.309

未经允许严禁编辑

Sample: 2026030902-QUAN
Operator:
Submitter:
File: D:\xyl\52688\1.SMP

Started: 2026/3/9 21:50:32
Completed: 2026/3/10 10:24:23
Report time: 2026/3/10 12:31:24
Sample mass: 0.2826 g
Analysis free space: 82.8911 cm³
Low pressure dose: 15.0000 cm³/g STP
Automatic degas: No

Analysis adsorptive: N2
Analysis bath temp.: 77.350 K
Thermal correction: Yes
Ambient free space: 27.7256 cm³ Measured
Equilibration interval: 10 s
Sample density: 1.000 g/cm³

Pore Table

Pore Width (nm)	Cumulative Pore Volume (cm³/g)	Incremental Pore Volume (cm³/g)	Cumulative Pore Area (m²/g)	Incremental Pore Area (m²/g)
1.090	0.24299	0.00684	697.617	12.551
1.179	0.24999	0.00700	709.487	11.871
1.269	0.25830	0.00832	722.596	13.108
1.358	0.26796	0.00966	736.817	14.221
1.483	0.27836	0.01040	750.834	14.017
1.591	0.28815	0.00979	763.147	12.313
1.716	0.29648	0.00833	772.860	9.713
1.859	0.30300	0.00652	779.875	7.015
2.002	0.30775	0.00475	784.622	4.747
2.162	0.31105	0.00330	787.671	3.049
2.341	0.31326	0.00222	789.566	1.894
2.520	0.31476	0.00150	790.754	1.189
2.734	0.31585	0.00108	791.548	0.793
2.949	0.31668	0.00084	792.117	0.569
3.181	0.31739	0.00070	792.559	0.443
3.431	0.31801	0.00063	792.924	0.365
3.699	0.31859	0.00057	793.233	0.309
4.003	0.31915	0.00057	793.516	0.283
4.325	0.31975	0.00060	793.792	0.276
4.664	0.32039	0.00065	794.069	0.277
5.040	0.32108	0.00069	794.341	0.273

未经允许严禁编辑

Sample: 2026030902-QUAN
Operator:
Submitter:
File: D:\xyl\52688\1.SMP

Started: 2026/3/9 21:50:32
Completed: 2026/3/10 10:24:23
Report time: 2026/3/10 12:31:24
Sample mass: 0.2826 g
Analysis free space: 82.8911 cm^3
Low pressure dose: 15.0000 cm^3/g STP
Automatic degas: No

Analysis adsorptive: N2
Analysis bath temp.: 77.350 K
Thermal correction: Yes
Ambient free space: 27.7256 cm^3 Measured
Equilibration interval: 10 s
Sample density: 1.000 g/cm^3

Pore Table

Pore Width (nm)	Cumulative Pore Volume (cm^3/g)	Incremental Pore Volume (cm^3/g)	Cumulative Pore Area (m^2/g)	Incremental Pore Area (m^2/g)
5.433	0.32180	0.00072	794.605	0.264
5.880	0.32254	0.00074	794.858	0.253
6.344	0.32330	0.00075	795.096	0.237
6.845	0.32401	0.00072	795.306	0.210
7.399	0.32465	0.00063	795.477	0.171
7.988	0.32525	0.00060	795.628	0.151
8.632	0.32593	0.00068	795.785	0.157
9.311	0.32666	0.00073	795.941	0.157
10.061	0.32732	0.00066	796.073	0.131
10.866	0.32793	0.00061	796.186	0.113
11.723	0.32854	0.00061	796.290	0.104
12.653	0.32916	0.00062	796.387	0.097
13.671	0.32977	0.00061	796.477	0.090
14.761	0.33038	0.00061	796.560	0.083
15.941	0.33099	0.00061	796.636	0.076
17.210	0.33157	0.00059	796.704	0.068
18.586	0.33214	0.00056	796.764	0.060
20.069	0.33268	0.00054	796.819	0.054
21.660	0.33320	0.00052	796.867	0.048
23.393	0.33370	0.00050	796.909	0.043
25.252	0.33418	0.00048	796.947	0.038

未经允许严禁编辑

Sample: 2026030902-QUAN
Operator:
Submitter:
File: D:\xyl\52688\1.SMP

Started: 2026/3/9 21:50:32
Completed: 2026/3/10 10:24:23
Report time: 2026/3/10 12:31:24
Sample mass: 0.2826 g
Analysis free space: 82.8911 cm^3
Low pressure dose: 15.0000 cm^3/g STP
Automatic degas: No

Analysis adsorptive: N2
Analysis bath temp.: 77.350 K
Thermal correction: Yes
Ambient free space: 27.7256 cm^3 Measured
Equilibration interval: 10 s
Sample density: 1.000 g/cm^3

Pore Table

Pore Width (nm)	Cumulative Pore Volume (cm^3/g)	Incremental Pore Volume (cm^3/g)	Cumulative Pore Area (m^2/g)	Incremental Pore Area (m^2/g)
27.271	0.33463	0.00046	796.981	0.033
29.451	0.33497	0.00034	797.004	0.023
31.792	0.33530	0.00033	797.024	0.020
34.330	0.33570	0.00040	797.048	0.023
37.064	0.33609	0.00039	797.069	0.021
40.031	0.33647	0.00038	797.087	0.019
43.230	0.33674	0.00027	797.100	0.013
46.679	0.33700	0.00026	797.111	0.011
50.396	0.33732	0.00032	797.124	0.013
54.417	0.33759	0.00028	797.134	0.010
58.760	0.33775	0.00015	797.139	0.005
63.442	0.33787	0.00013	797.143	0.004
68.499	0.33808	0.00021	797.149	0.006
73.968	0.33829	0.00022	797.155	0.006
79.865	0.33859	0.00030	797.162	0.007
86.245	0.33907	0.00048	797.173	0.011
93.126	0.33953	0.00046	797.183	0.010
100.560	0.33985	0.00032	797.190	0.006
108.566	0.34014	0.00029	797.195	0.005
117.233	0.34050	0.00036	797.201	0.006
126.580	0.34075	0.00025	797.205	0.004

未经允许严禁编辑

Sample: 2026030902-QUAN
Operator:
Submitter:
File: D:\xyl\52688\1.SMP

Started: 2026/3/9 21:50:32
Completed: 2026/3/10 10:24:23
Report time: 2026/3/10 12:31:24
Sample mass: 0.2826 g
Analysis free space: 82.8911 cm³
Low pressure dose: 15.0000 cm³/g STP
Automatic degas: No

Analysis adsorptive: N2
Analysis bath temp.: 77.350 K
Thermal correction: Yes
Ambient free space: 27.7256 cm³ Measured
Equilibration interval: 10 s
Sample density: 1.000 g/cm³

Pore Table

Pore Width (nm)	Cumulative Pore Volume (cm³/g)	Incremental Pore Volume (cm³/g)	Cumulative Pore Area (m²/g)	Incremental Pore Area (m²/g)
136.677	0.34098	0.00023	797.209	0.003
147.596	0.34126	0.00028	797.212	0.004
159.355	0.34146	0.00020	797.215	0.002
172.079	0.34164	0.00018	797.217	0.002
185.804	0.34186	0.00022	797.219	0.002
200.619	0.34201	0.00015	797.221	0.002
216.632	0.34215	0.00014	797.222	0.001
233.913	0.34229	0.00013	797.223	0.001
252.570	0.34241	0.00012	797.224	0.001
272.729	0.34256	0.00015	797.225	0.001
294.478	0.34266	0.00010	797.226	0.001
317.978	0.34276	0.00009	797.227	0.001

未经允许严禁编辑

Sample: 2026030902-QUAN
Operator:
Submitter:
File: D:\xyl\52688\1.SMP

Started: 2026/3/9 21:50:32
Completed: 2026/3/10 10:24:23
Report time: 2026/3/10 12:31:24
Sample mass: 0.2826 g
Analysis free space: 82.8911 cm³
Low pressure dose: 15.0000 cm³/g STP
Automatic degas: No

Analysis adsorptive: N2
Analysis bath temp.: 77.350 K
Thermal correction: Yes
Ambient free space: 27.7256 cm³ Measured
Equilibration interval: 10 s
Sample density: 1.000 g/cm³

Porosity Distribution by Invalid
Model: N2 - DFT Model
Method: Non-negative Regularization: 0.02300
Standard Deviation of Fit: 1.80494 cm³/g STP

Isotherm Table

Relative Pressure (p/p°)	Experimental Quantity Adsorbed (cm³/g STP)	Fitted Quantity Adsorbed (cm³/g STP)	Absolute Residual (cm³/g STP)	Relative Residual
0.000002783	22.0279	40.8251	-18.7971	-0.853330
0.000005075	76.4118	67.3701	9.0417	0.118329
0.000008995	90.4842	87.7867	2.6975	0.029812
0.000015520	102.8285	96.4672	6.3613	0.061864
0.000026102	113.3700	110.8463	2.5237	0.022261
0.000042841	123.1642	121.5634	1.6008	0.012997
0.000068697	132.9930	130.9372	2.0558	0.015458
0.000107744	140.2164	139.7614	0.4550	0.003245
0.000165451	148.2546	147.9203	0.3343	0.002255
0.000249000	153.5859	155.0298	-1.4440	-0.009402
0.000367617	159.9521	161.1902	-1.2381	-0.007741
0.000532902	165.1939	166.9330	-1.7391	-0.010528
0.000759152	169.0358	172.0230	-2.9872	-0.017672
0.001063641	173.9617	176.9898	-3.0281	-0.017407
0.001466847	178.7450	180.6962	-1.9512	-0.010916

未经允许严禁编辑

Sample: 2026030902-QUAN
Operator:
Submitter:
File: D:\xyl\52688\1.SMP

Started: 2026/3/9 21:50:32
Completed: 2026/3/10 10:24:23
Report time: 2026/3/10 12:31:24
Sample mass: 0.2826 g
Analysis free space: 82.8911 cm³
Low pressure dose: 15.0000 cm³/g STP
Automatic degas: No

Analysis adsorptive: N2
Analysis bath temp.: 77.350 K
Thermal correction: Yes
Ambient free space: 27.7256 cm³ Measured
Equilibration interval: 10 s
Sample density: 1.000 g/cm³

Isotherm Table

Relative Pressure (p/p°)	Experimental Quantity Adsorbed (cm³/g STP)	Fitted Quantity Adsorbed (cm³/g STP)	Absolute Residual (cm³/g STP)	Relative Residual
0.001992604	181.6207	183.9714	-2.3507	-0.012943
0.002668156	185.1488	187.5227	-2.3739	-0.012822
0.003524104	189.3068	190.3657	-1.0589	-0.005594
0.004594232	193.1714	192.8843	0.2871	0.001486
0.005915212	196.4269	196.2841	0.1428	0.000727
0.007526182	199.9931	198.7566	1.2365	0.006183
0.009468212	203.0775	203.1847	-0.1072	-0.000528
0.011783670	205.6243	205.2414	0.3828	0.001862
0.014515520	208.3550	207.1264	1.2287	0.005897
0.017706521	211.2624	211.5791	-0.3167	-0.001499
0.021398440	214.2332	213.3063	0.9269	0.004327
0.025631230	217.1003	214.9545	2.1459	0.009884
0.030442240	219.6298	219.0351	0.5947	0.002708
0.035865448	221.9111	220.5665	1.3446	0.006059
0.041930798	224.3010	224.4896	-0.1886	-0.000841
0.048663601	226.4799	225.8758	0.6042	0.002668
0.056084011	228.2774	229.0792	-0.8019	-0.003513
0.064206667	230.1675	230.3330	-0.1655	-0.000719
0.073040441	232.0043	231.5257	0.4786	0.002063
0.082588248	233.5681	234.0358	-0.4678	-0.002003

未经允许严禁编辑

Sample: 2026030902-QUAN
Operator:
Submitter:
File: D:\xyl\52688\1.SMP

Started: 2026/3/9 21:50:32
Completed: 2026/3/10 10:24:23
Report time: 2026/3/10 12:31:24
Sample mass: 0.2826 g
Analysis free space: 82.8911 cm³
Low pressure dose: 15.0000 cm³/g STP
Automatic degas: No

Analysis adsorptive: N2
Analysis bath temp.: 77.350 K
Thermal correction: Yes
Ambient free space: 27.7256 cm³ Measured
Equilibration interval: 10 s
Sample density: 1.000 g/cm³

Isotherm Table

Relative Pressure (p/p°)	Experimental Quantity Adsorbed (cm³/g STP)	Fitted Quantity Adsorbed (cm³/g STP)	Absolute Residual (cm³/g STP)	Relative Residual
0.092847057	235.0596	235.0763	-0.0168	-0.000071
0.103808001	236.4045	236.0486	0.3559	0.001505
0.115456402	237.6613	237.9174	-0.2561	-0.001077
0.127772301	238.7952	238.7451	0.0501	0.000210
0.140730694	239.8466	239.5166	0.3300	0.001376
0.154301897	240.8036	240.9044	-0.1008	-0.000418
0.168452203	241.6898	241.5731	0.1167	0.000483
0.183144197	242.5136	242.6683	-0.1547	-0.000638
0.198337302	243.2716	243.2546	0.0170	0.000070
0.213988706	243.9613	243.8070	0.1543	0.000633
0.230053306	244.6225	244.6821	-0.0596	-0.000244
0.246484801	245.2404	245.1732	0.0672	0.000274
0.263235897	245.8150	245.6389	0.1760	0.000716
0.280259013	246.3580	246.3612	-0.0031	-0.000013
0.297506303	246.8785	246.7803	0.0982	0.000398
0.314930797	247.3611	247.4234	-0.0623	-0.000252
0.332486212	247.8236	247.8037	0.0199	0.000080
0.350127310	248.2727	248.1680	0.1047	0.000422
0.367810607	248.7099	248.7374	-0.0275	-0.000111
0.385494202	249.1185	249.0702	0.0483	0.000194

未经允许严禁编辑

Sample: 2026030902-QUAN
Operator:
Submitter:
File: D:\xyl\52688\1.SMP

Started: 2026/3/9 21:50:32
Completed: 2026/3/10 10:24:23
Report time: 2026/3/10 12:31:24
Sample mass: 0.2826 g
Analysis free space: 82.8911 cm³
Low pressure dose: 15.0000 cm³/g STP
Automatic degas: No

Analysis adsorptive: N2
Analysis bath temp.: 77.350 K
Thermal correction: Yes
Ambient free space: 27.7256 cm³ Measured
Equilibration interval: 10 s
Sample density: 1.000 g/cm³

Isotherm Table

Relative Pressure (p/p°)	Experimental Quantity Adsorbed (cm³/g STP)	Fitted Quantity Adsorbed (cm³/g STP)	Absolute Residual (cm³/g STP)	Relative Residual
0.403138310	249.5057	249.3894	0.1163	0.000466
0.420704991	249.8780	249.8971	-0.0191	-0.000076
0.438158900	250.2418	250.1857	0.0561	0.000224
0.455466807	250.5971	250.6663	-0.0692	-0.000276
0.472598106	250.9406	250.9250	0.0156	0.000062
0.489524394	251.2738	251.1708	0.1030	0.000410
0.506219923	251.5952	251.6253	-0.0301	-0.000120
0.522661209	251.9020	251.8454	0.0566	0.000225
0.538827300	252.2057	252.3013	-0.0956	-0.000379
0.554699600	252.5205	252.4994	0.0211	0.000084
0.570261598	252.8125	252.6887	0.1238	0.000490
0.585499227	253.0981	253.1377	-0.0397	-0.000157
0.600400090	253.3873	253.3098	0.0775	0.000306
0.614954293	253.6540	253.7619	-0.1079	-0.000426
0.629153311	253.9251	253.9193	0.0058	0.000023
0.642990828	254.1989	254.0711	0.1278	0.000503
0.656461716	254.4707	254.5212	-0.0505	-0.000198
0.669562697	254.7294	254.6607	0.0687	0.000270
0.682291925	254.9823	255.1090	-0.1267	-0.000497
0.694648683	255.2385	255.2364	0.0021	0.000008

未经允许严禁编辑

Sample: 2026030902-QUAN
Operator:
Submitter:
File: D:\xyl\52688\1.SMP

Started: 2026/3/9 21:50:32
Completed: 2026/3/10 10:24:23
Report time: 2026/3/10 12:31:24
Sample mass: 0.2826 g
Analysis free space: 82.8911 cm³
Low pressure dose: 15.0000 cm³/g STP
Automatic degas: No

Analysis adsorptive: N2
Analysis bath temp.: 77.350 K
Thermal correction: Yes
Ambient free space: 27.7256 cm³ Measured
Equilibration interval: 10 s
Sample density: 1.000 g/cm³

Isotherm Table

Relative Pressure (p/p°)	Experimental Quantity Adsorbed (cm³/g STP)	Fitted Quantity Adsorbed (cm³/g STP)	Absolute Residual (cm³/g STP)	Relative Residual
0.706633508	255.4911	255.3589	0.1322	0.000517
0.718248010	255.7379	255.7796	-0.0416	-0.000163
0.729494929	255.9804	255.8897	0.0907	0.000355
0.740377605	256.2155	256.2679	-0.0525	-0.000205
0.750900388	256.4420	256.3669	0.0751	0.000293
0.761068285	256.6603	256.7274	-0.0671	-0.000262
0.770887017	256.8712	256.8167	0.0545	0.000212
0.780362606	257.0755	257.2079	-0.1324	-0.000515
0.789501607	257.2851	257.2888	-0.0037	-0.000014
0.798311174	257.5030	257.3667	0.1362	0.000529
0.806798697	257.7230	257.7753	-0.0523	-0.000203
0.814971626	257.9401	257.8465	0.0936	0.000363
0.822837889	258.1515	258.2236	-0.0721	-0.000279
0.830405474	258.3626	258.2892	0.0734	0.000284
0.837682605	258.5728	258.6441	-0.0713	-0.000276
0.844677329	258.7804	258.7043	0.0761	0.000294
0.851397991	258.9843	259.0553	-0.0710	-0.000274
0.857852995	259.1878	259.1100	0.0778	0.000300
0.864050388	259.3911	259.4632	-0.0720	-0.000278
0.869998574	259.5927	259.5124	0.0804	0.000310

未经允许严禁编辑

Sample: 2026030902-QUAN
Operator:
Submitter:
File: D:\xyl\52688\1.SMP

Started: 2026/3/9 21:50:32
Completed: 2026/3/10 10:24:23
Report time: 2026/3/10 12:31:24
Sample mass: 0.2826 g
Analysis free space: 82.8911 cm³
Low pressure dose: 15.0000 cm³/g STP
Automatic degas: No

Analysis adsorptive: N2
Analysis bath temp.: 77.350 K
Thermal correction: Yes
Ambient free space: 27.7256 cm³ Measured
Equilibration interval: 10 s
Sample density: 1.000 g/cm³

Isotherm Table

Relative Pressure (p/p°)	Experimental Quantity Adsorbed (cm³/g STP)	Fitted Quantity Adsorbed (cm³/g STP)	Absolute Residual (cm³/g STP)	Relative Residual
0.875705481	259.7915	259.8640	-0.0726	-0.000279
0.881179392	259.9867	259.9082	0.0785	0.000302
0.886428118	260.1823	260.2581	-0.0758	-0.000291
0.891459525	260.3779	260.2980	0.0799	0.000307
0.896281302	260.5717	260.6462	-0.0745	-0.000286
0.900900900	260.7623	260.6823	0.0800	0.000307
0.905325770	260.9487	261.0205	-0.0718	-0.000275
0.909563184	261.1300	261.0536	0.0764	0.000292
0.913620114	261.3081	261.3794	-0.0713	-0.000273
0.917503417	261.4843	261.4098	0.0744	0.000285
0.921219707	261.6577	261.7273	-0.0695	-0.000266
0.924775481	261.8280	261.7550	0.0729	0.000279
0.928177178	261.9945	262.0615	-0.0670	-0.000256
0.931430817	262.1569	262.0865	0.0704	0.000269
0.934542298	262.3149	262.3788	-0.0639	-0.000243
0.937517405	262.4684	262.4012	0.0672	0.000256
0.940361619	262.6175	262.6818	-0.0643	-0.000245
0.943080306	262.7656	262.7020	0.0636	0.000242
0.945678592	262.9124	262.9725	-0.0601	-0.000229
0.948161721	263.0570	262.9908	0.0663	0.000252

未经允许严禁编辑

Sample: 2026030902-QUAN
Operator:
Submitter:
File: D:\xyl\52688\1.SMP

Started: 2026/3/9 21:50:32
Completed: 2026/3/10 10:24:23
Report time: 2026/3/10 12:31:24
Sample mass: 0.2826 g
Analysis free space: 82.8911 cm³
Low pressure dose: 15.0000 cm³/g STP
Automatic degas: No

Analysis adsorptive: N2
Analysis bath temp.: 77.350 K
Thermal correction: Yes
Ambient free space: 27.7256 cm³ Measured
Equilibration interval: 10 s
Sample density: 1.000 g/cm³

Isotherm Table

Relative Pressure (p/p°)	Experimental Quantity Adsorbed (cm³/g STP)	Fitted Quantity Adsorbed (cm³/g STP)	Absolute Residual (cm³/g STP)	Relative Residual
0.950534225	263.1988	263.1977	0.0011	0.000004
0.952800930	263.3372	263.3967	-0.0595	-0.000226
0.954966187	263.4717	263.4127	0.0589	0.000224
0.957034409	263.6020	263.6556	-0.0536	-0.000203
0.959009588	263.7279	263.6703	0.0576	0.000218
0.960896015	263.8508	263.9056	-0.0548	-0.000208
0.962697208	263.9735	263.9189	0.0546	0.000207
0.964416981	264.0949	264.1469	-0.0520	-0.000197
0.966058910	264.2137	264.1588	0.0549	0.000208
0.967626274	264.3293	264.3284	0.0009	0.000003
0.969122529	264.4409	264.4892	-0.0483	-0.000183
0.970550597	264.5480	264.4994	0.0486	0.000184
0.971913695	264.6505	264.6940	-0.0435	-0.000164
0.973214507	264.7481	264.7034	0.0447	0.000169
0.974455774	264.8406	264.8752	-0.0346	-0.000131
0.975640416	264.9200	264.8839	0.0361	0.000136
0.976770699	264.9822	264.9815	0.0007	0.000003
0.977849126	265.0383	265.0638	-0.0255	-0.000096
0.978878021	265.0970	265.0714	0.0256	0.000097
0.979859591	265.1649	265.2016	-0.0367	-0.000138

未经允许严禁编辑

Sample: 2026030902-QUAN
Operator:
Submitter:
File: D:\xyl\52688\1.SMP

Started: 2026/3/9 21:50:32
Completed: 2026/3/10 10:24:23
Report time: 2026/3/10 12:31:24
Sample mass: 0.2826 g
Analysis free space: 82.8911 cm³
Low pressure dose: 15.0000 cm³/g STP
Automatic degas: No

Analysis adsorptive: N_2
Analysis bath temp.: 77.350 K
Thermal correction: Yes
Ambient free space: 27.7256 cm³ Measured
Equilibration interval: 10 s
Sample density: 1.000 g/cm³

Isotherm Table

Relative Pressure (p/p°)	Experimental Quantity Adsorbed (cm³/g STP)	Fitted Quantity Adsorbed (cm³/g STP)	Absolute Residual (cm³/g STP)	Relative Residual
0.980795979	265.2464	265.2085	0.0379	0.000143
0.981689274	265.3449	265.3442	0.0007	0.000003
0.982541502	265.4623	265.5279	-0.0656	-0.000247
0.983354270	265.5995	265.5338	0.0656	0.000247
0.984129488	265.7542	265.8269	-0.0727	-0.000274
0.984869003	265.9059	265.8323	0.0735	0.000277
0.985574186	266.0505	266.1167	-0.0661	-0.000249
0.986246824	266.1885	266.1216	0.0669	0.000251
0.986888289	266.3201	266.3197	0.0003	0.000001
0.987500012	266.4456	266.5033	-0.0577	-0.000217
0.988083303	266.5652	266.5074	0.0578	0.000217
0.988639593	266.6794	266.7316	-0.0522	-0.000196
0.989170074	266.7882	266.7352	0.0529	0.000198
0.989675879	266.8919	266.8917	0.0003	0.000001
0.990158200	266.9909	267.0364	-0.0455	-0.000170
0.990618110	267.0852	267.0395	0.0457	0.000171
0.991056621	267.1752	267.2164	-0.0412	-0.000154
0.991474688	267.2609	267.2192	0.0417	0.000156
0.991873324	267.3427	267.3425	0.0002	0.000001
0.992253423	267.4207	267.4566	-0.0359	-0.000134

未经允许严禁编辑

Sample: 2026030902-QUAN
Operator:
Submitter:
File: D:\xyl\52688\1.SMP

Started: 2026/3/9 21:50:32
Completed: 2026/3/10 10:24:23
Report time: 2026/3/10 12:31:24
Sample mass: 0.2826 g
Analysis free space: 82.8911 cm³
Low pressure dose: 15.0000 cm³/g STP
Automatic degas: No

Analysis adsorptive: N2
Analysis bath temp.: 77.350 K
Thermal correction: Yes
Ambient free space: 27.7256 cm³ Measured
Equilibration interval: 10 s
Sample density: 1.000 g/cm³

Isotherm Table

Relative Pressure (p/p°)	Experimental Quantity Adsorbed (cm³/g STP)	Fitted Quantity Adsorbed (cm³/g STP)	Absolute Residual (cm³/g STP)	Relative Residual
0.992615700	267.4950	267.4589	0.0361	0.000135
0.992961228	267.5659	267.5985	-0.0326	-0.000122
0.993290603	267.6335	267.6006	0.0329	0.000123
0.993604600	267.6979	267.6977	0.0002	0.000001
0.993903875	267.7593	267.7876	-0.0283	-0.000106
0.994189322	267.8178	267.7893	0.0285	0.000106
0.994461298	267.8736	267.8734	0.0002	0.000001
0.994720697	267.9268	267.9514	-0.0245	-0.000092
0.994967878	267.9775	267.9529	0.0247	0.000092
0.995203614	268.0259	268.0482	-0.0223	-0.000083
0.995428324	268.0720	268.0495	0.0225	0.000084
0.995642424	268.1159	268.1158	0.0001	0.000000
0.995846629	268.1578	268.1764	-0.0186	-0.000069
0.996041179	268.1977	268.1775	0.0202	0.000075

未经允许严禁编辑

Sample: 2026030902-QUAN
Operator:
Submitter:
File: D:\xyl\52688\1.SMP

Started: 2026/3/9 21:50:32
Completed: 2026/3/10 10:24:23
Report time: 2026/3/10 12:31:24
Sample mass: 0.2826 g
Analysis free space: 82.8911 cm³
Low pressure dose: 15.0000 cm³/g STP
Automatic degas: No

Analysis adsorptive: N2
Analysis bath temp.: 77.350 K
Thermal correction: Yes
Ambient free space: 27.7256 cm³ Measured
Equilibration interval: 10 s
Sample density: 1.000 g/cm³

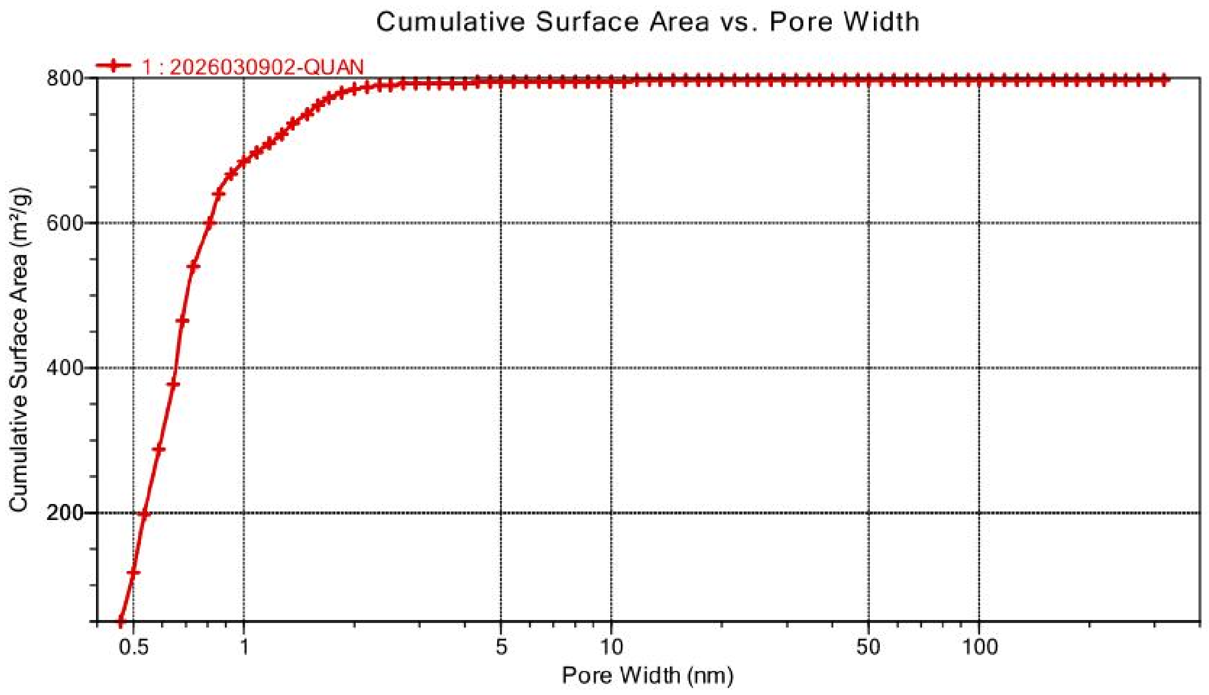

未经允许严禁编辑

Sample: 2026030902-QUAN
Operator:
Submitter:
File: D:\xyl\52688\1.SMP

Started:	2026/3/9 21:50:32	Analysis adsorptive:	N2
Completed:	2026/3/10 10:24:23	Analysis bath temp.:	77.350 K
Report time:	2026/3/10 12:31:24	Thermal correction:	Yes
Sample mass:	0.2826 g	Ambient free space:	27.7256 cm³ Measured
Analysis free space:	82.8911 cm³	Equilibration interval:	10 s
Low pressure dose:	15.0000 cm³/g STP	Sample density:	1.000 g/cm³
Automatic degas:	No		

Incremental Surface Area vs. Pore Width

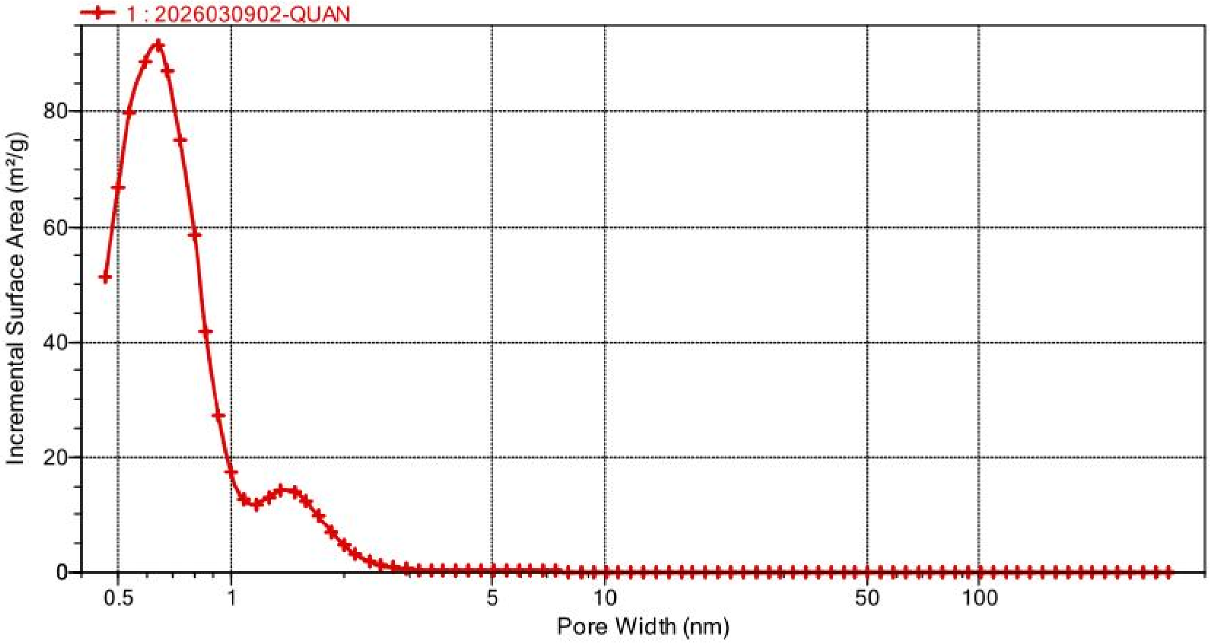

未经允许严禁编辑

Sample: 2026030902-QUAN
Operator:
Submitter:
File: D:\xyl\52688\1.SMP

Started: 2026/3/9 21:50:32
Completed: 2026/3/10 10:24:23
Report time: 2026/3/10 12:31:24
Sample mass: 0.2826 g
Analysis free space: 82.8911 cm³
Low pressure dose: 15.0000 cm³/g STP
Automatic degas: No

Analysis adsorptive: N2
Analysis bath temp.: 77.350 K
Thermal correction: Yes
Ambient free space: 27.7256 cm³ Measured
Equilibration interval: 10 s
Sample density: 1.000 g/cm³

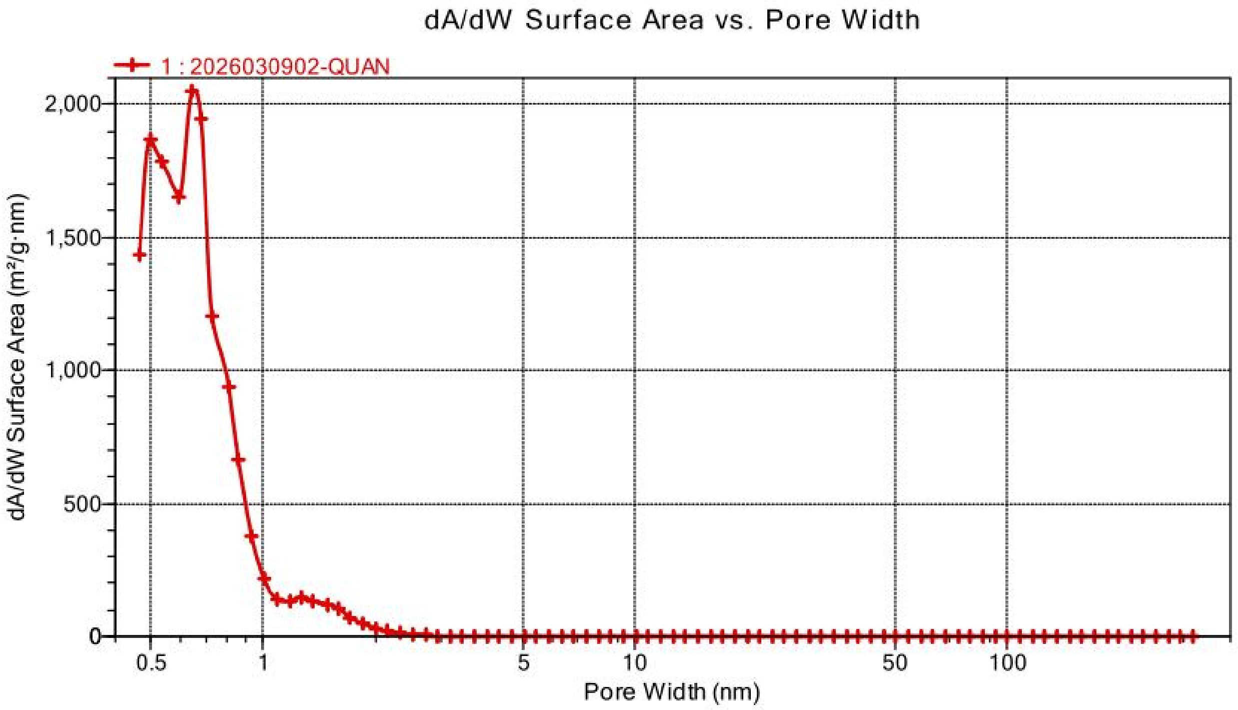

未经允许严禁编辑

Sample: 2026030902-QUAN
Operator:
Submitter:
File: D:\xyl\52688\1.SMP

Started:	2026/3/9 21:50:32	Analysis adsorptive:	N2
Completed:	2026/3/10 10:24:23	Analysis bath temp.:	77.350 K
Report time:	2026/3/10 12:31:24	Thermal correction:	Yes
Sample mass:	0.2826 g	Ambient free space:	27.7256 cm³ Measured
Analysis free space:	82.8911 cm³	Equilibration interval:	10 s
Low pressure dose:	15.0000 cm³/g STP	Sample density:	1.000 g/cm³
Automatic degas:	No		

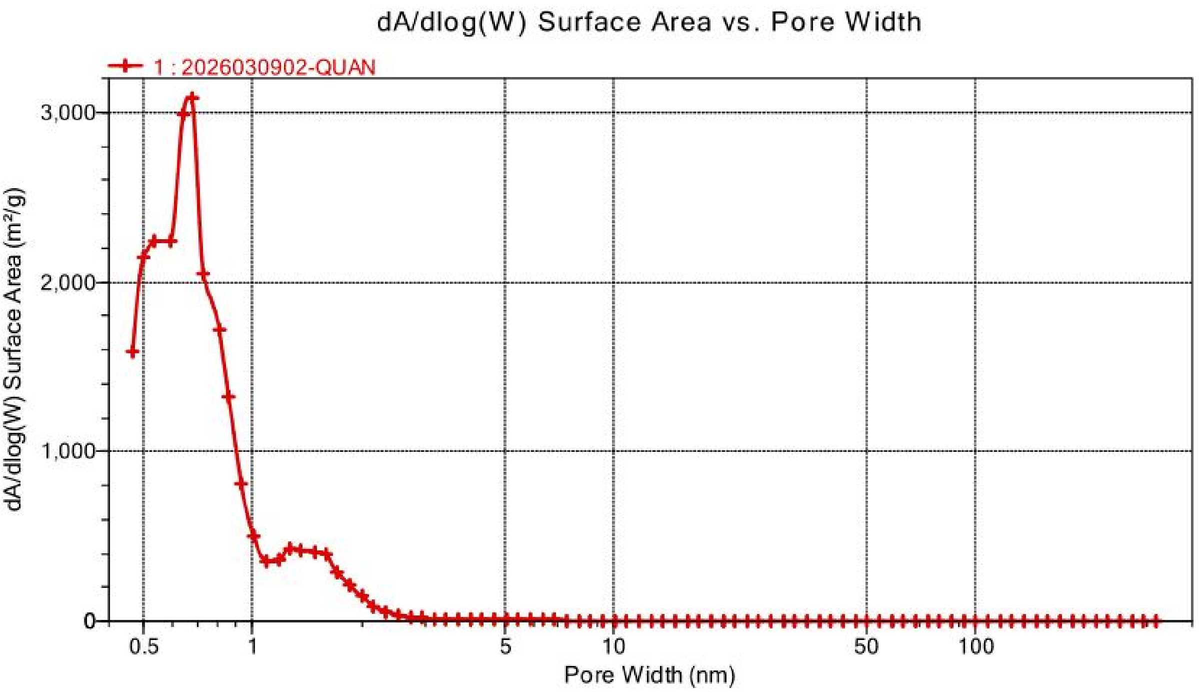

未经允许严禁编辑

Sample: 2026030902-QUAN
Operator:
Submitter:
File: D:\xyl\52688\1.SMP

Started:	2026/3/9 21:50:32	Analysis adsorptive:	N2
Completed:	2026/3/10 10:24:23	Analysis bath temp.:	77.350 K
Report time:	2026/3/10 12:31:24	Thermal correction:	Yes
Sample mass:	0.2826 g	Ambient free space:	27.7256 cm³ Measured
Analysis free space:	82.8911 cm³	Equilibration interval:	10 s
Low pressure dose:	15.0000 cm³/g STP	Sample density:	1.000 g/cm³
Automatic degas:	No		

Cumulative Pore Volume vs. Pore Width

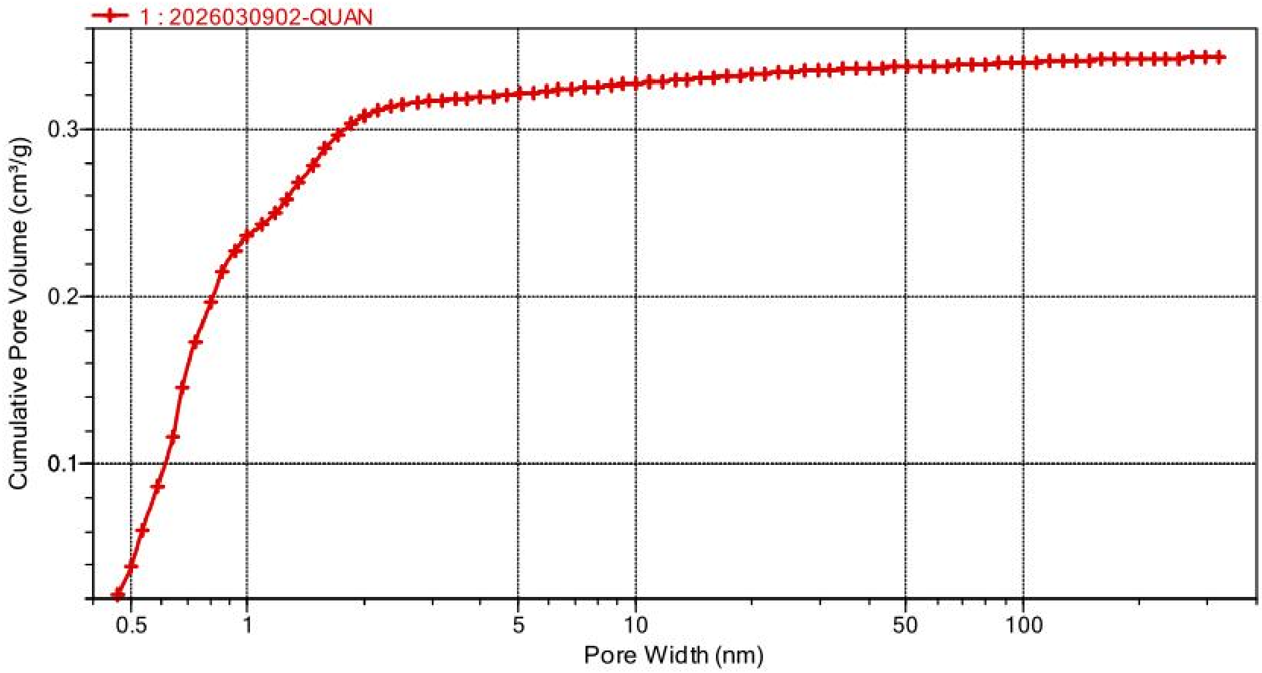

未经允许严禁编辑

Sample: 2026030902-QUAN
Operator:
Submitter:
File: D:\xyl\52688\1.SMP

Started:	2026/3/9 21:50:32	Analysis adsorptive:	N2
Completed:	2026/3/10 10:24:23	Analysis bath temp.:	77.350 K
Report time:	2026/3/10 12:31:24	Thermal correction:	Yes
Sample mass:	0.2826 g	Ambient free space:	27.7256 cm³ Measured
Analysis free space:	82.8911 cm³	Equilibration interval:	10 s
Low pressure dose:	15.0000 cm³/g STP	Sample density:	1.000 g/cm³
Automatic degas:	No		

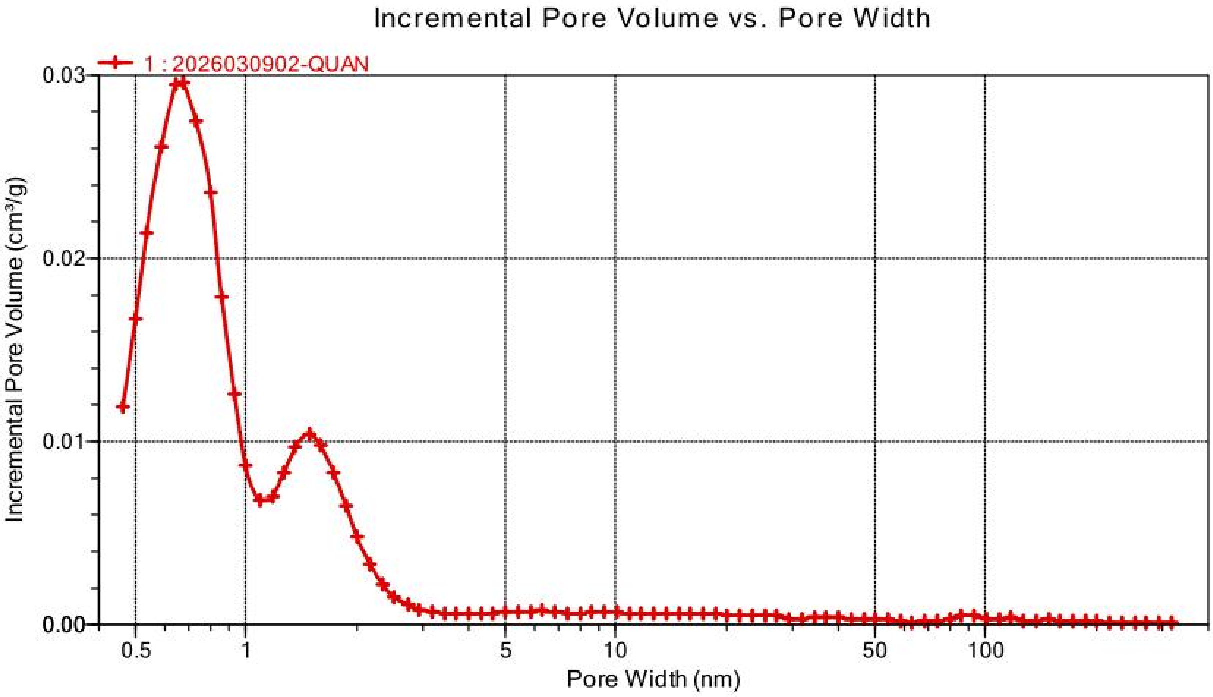

未经允许严禁编辑

Sample: 2026030902-QUAN
Operator:
Submitter:
File: D:\xyl\52688\1.SMP

Started: 2026/3/9 21:50:32
Completed: 2026/3/10 10:24:23
Report time: 2026/3/10 12:31:24
Sample mass: 0.2826 g
Analysis free space: 82.8911 cm³
Low pressure dose: 15.0000 cm³/g STP
Automatic degas: No

Analysis adsorptive: N2
Analysis bath temp.: 77.350 K
Thermal correction: Yes
Ambient free space: 27.7256 cm³ Measured
Equilibration interval: 10 s
Sample density: 1.000 g/cm³

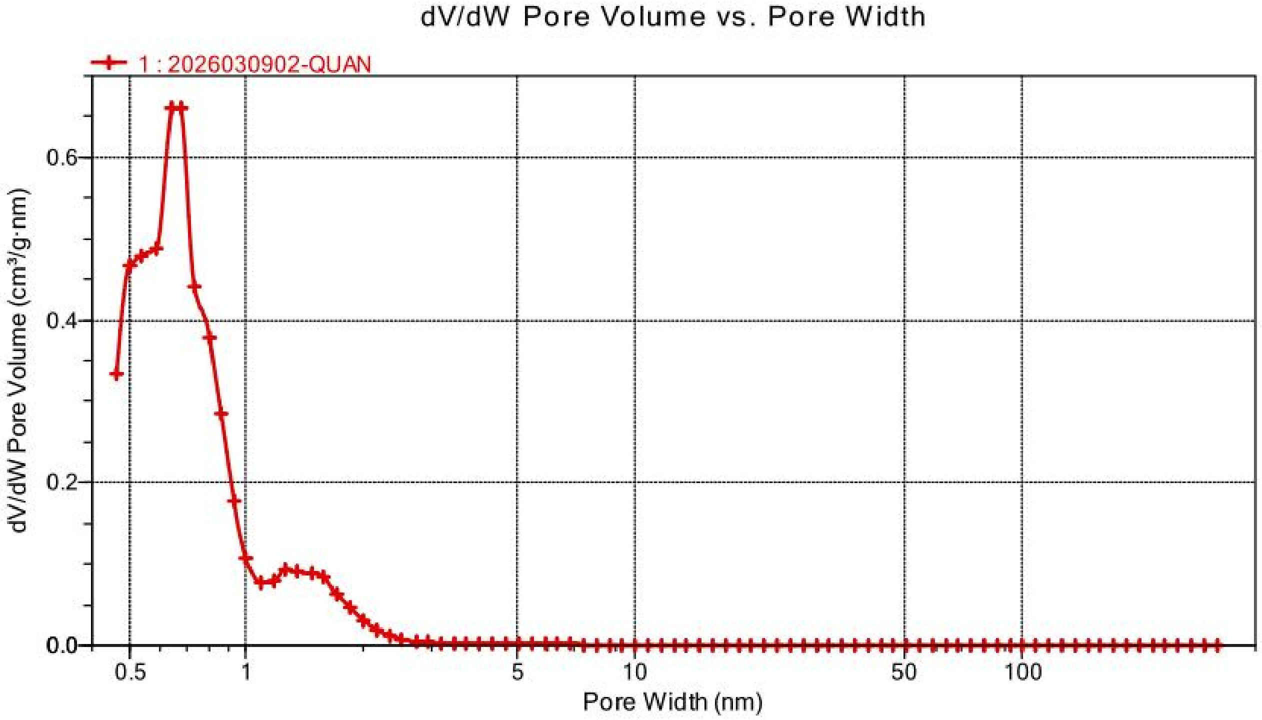

未经允许严禁编辑

Sample: 2026030902-QUAN
Operator:
Submitter:
File: D:\xyl\52688\1.SMP

Started:	2026/3/9 21:50:32	Analysis adsorptive:	N2
Completed:	2026/3/10 10:24:23	Analysis bath temp.:	77.350 K
Report time:	2026/3/10 12:31:24	Thermal correction:	Yes
Sample mass:	0.2826 g	Ambient free space:	27.7256 cm³ Measured
Analysis free space:	82.8911 cm³	Equilibration interval:	10 s
Low pressure dose:	15.0000 cm³/g STP	Sample density:	1.000 g/cm³
Automatic degas:	No		

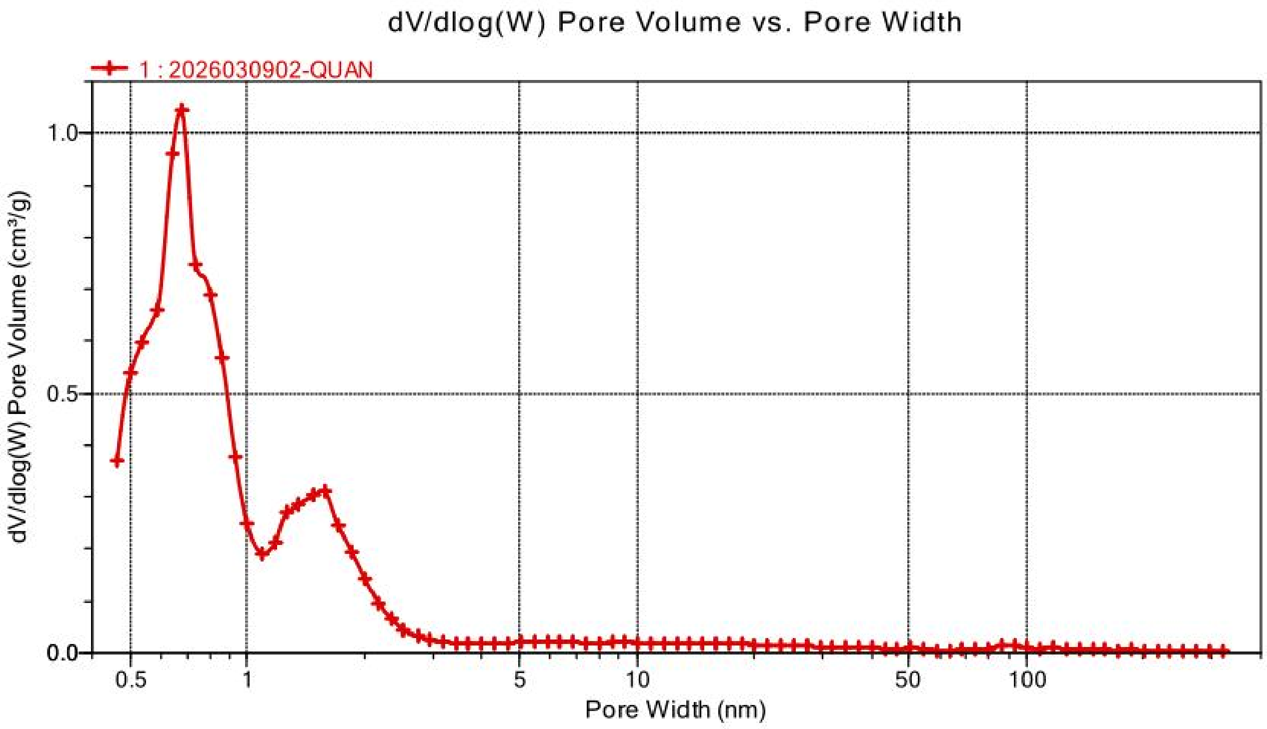

未经允许严禁编辑

Sample: 2026030902-QUAN
Operator:
Submitter:
File: D:\xyl\52688\1.SMP

Started:	2026/3/9 21:50:32	Analysis adsorptive:	N2
Completed:	2026/3/10 10:24:23	Analysis bath temp.:	77.350 K
Report time:	2026/3/10 12:31:24	Thermal correction:	Yes
Sample mass:	0.2826 g	Ambient free space:	27.7256 cm³ Measured
Analysis free space:	82.8911 cm³	Equilibration interval:	10 s
Low pressure dose:	15.0000 cm³/g STP	Sample density:	1.000 g/cm³
Automatic degas:	No		

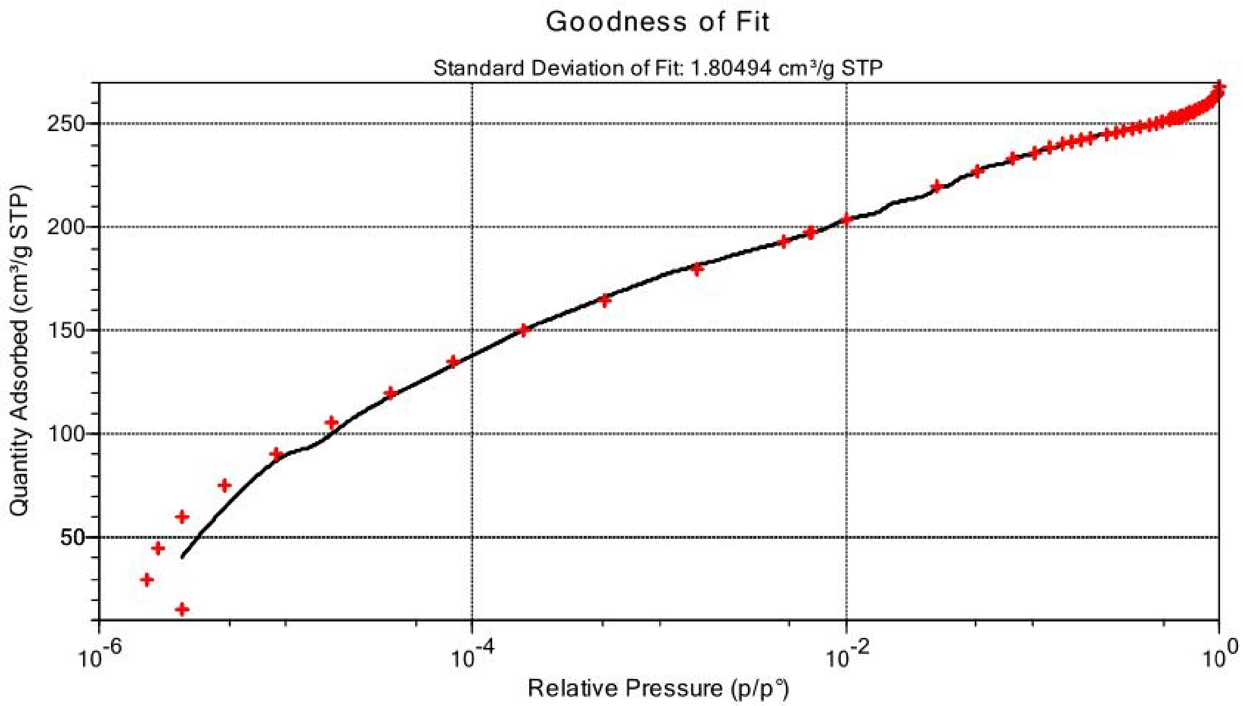

未经允许严禁编辑

Sample: 2026030902-QUAN
Operator:
Submitter:
File: D:\xyl\52688\1.SMP

Started: 2026/3/9 21:50:32
Completed: 2026/3/10 10:24:23
Report time: 2026/3/10 12:31:24
Sample mass: 0.2826 g
Analysis free space: 82.8911 cm³
Low pressure dose: 15.0000 cm³/g STP
Automatic degas: No

Analysis adsorptive: N2
Analysis bath temp.: 77.350 K
Thermal correction: Yes
Ambient free space: 27.7256 cm³ Measured
Equilibration interval: 10 s
Sample density: 1.000 g/cm³

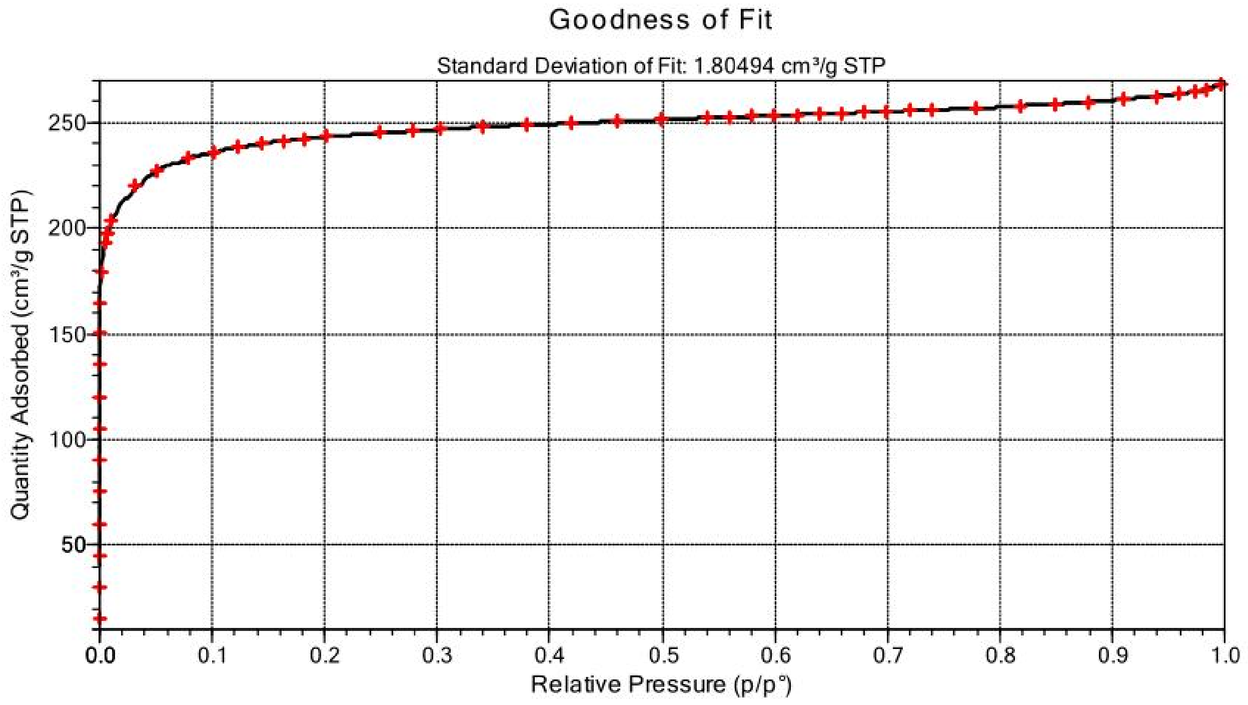

TEST TWO： Standard test

Test item	Method Reference	Result
Ash	GB/T 12496.3-1999	3.82(%)
pH (100g/L aqueous solution, 25° C)	GB/T 7702.16-1997	8.3

报告结束

未经允许严禁编辑

Additional Instructions of the Report:

1. This report is invalid without the special seal for reporting and the signature of the reviewer. No modification, addition or deletion of the report content is allowed without authorization.
2. The test results described in this report are only responsible for the samples provided by the client. If there are any objections to the report results, they should be raised to this laboratory within one week from the date of receiving this report. Objections raised after this period will not be accepted.
3. The data in this report is the technical service support provided by our institution to the client. The client should use the data content in this report with caution. Any risks, losses and legal consequences that may arise from the use of the data content in this report shall be borne by the client.
4. This institution fulfills the obligation of confidentiality regarding the technical documents, contract documents, report texts and other business secrets of the entrusted units.
5. Reports without the CMA qualification certification mark, data and results are only used for scientific research, teaching and internal quality control purposes and are not regarded as social impartiality data. The content of the Chinese and English reports shall be subject to the Chinese version.

未经允许严禁编辑
