# Supplementary material for: Silicon Combined with Activated Carbon Enhances Salt Tolerance in Strawberry (Fragaria × ananassa) by Reinforcing Ion–Redox Homeostasis and Reshaping the Rhizosphere Microbiome
Source: Plants (Basel). 2026 Apr 9;15(8):1154. doi: 10.3390/plants15081154 (PMC13119609; doi:10.3390/plants15081154)
Supplement: Supplementary file 1 [file plants-15-01154-s001.zip › Supplementary_Figures.pdf]

Supplementary Materials

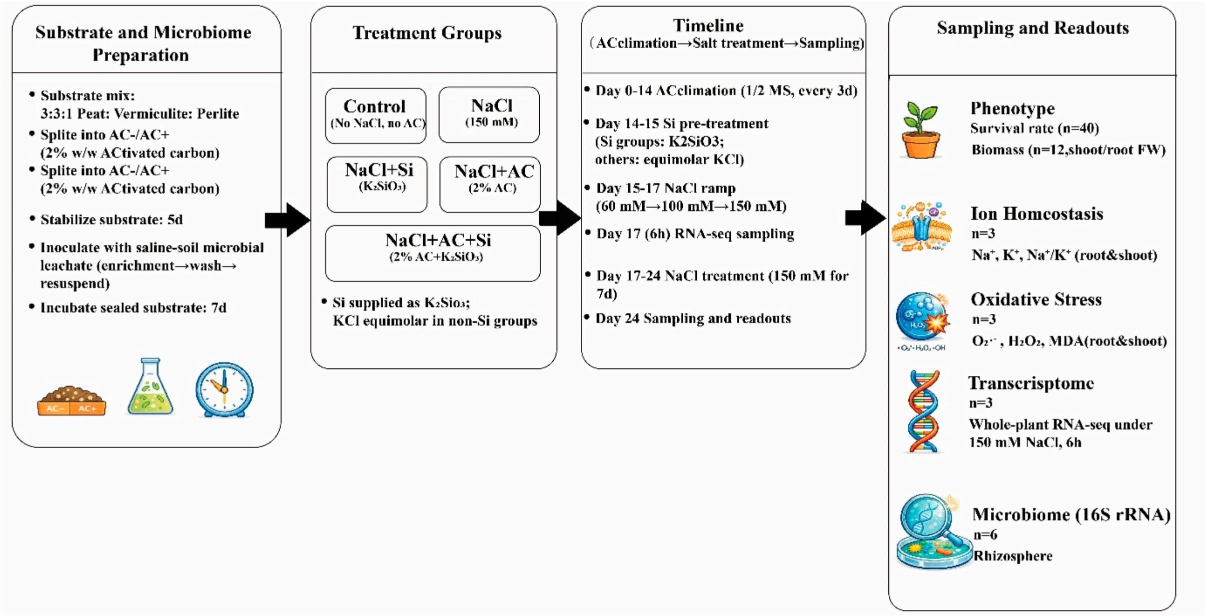

Figure S1. Experimental design and treatment scheme. Overview of treatment groups, salinity regime (60→100→150 mM NaCl), sampling points (including 6 h transcriptome sampling), and downstream measurements performed in this study.

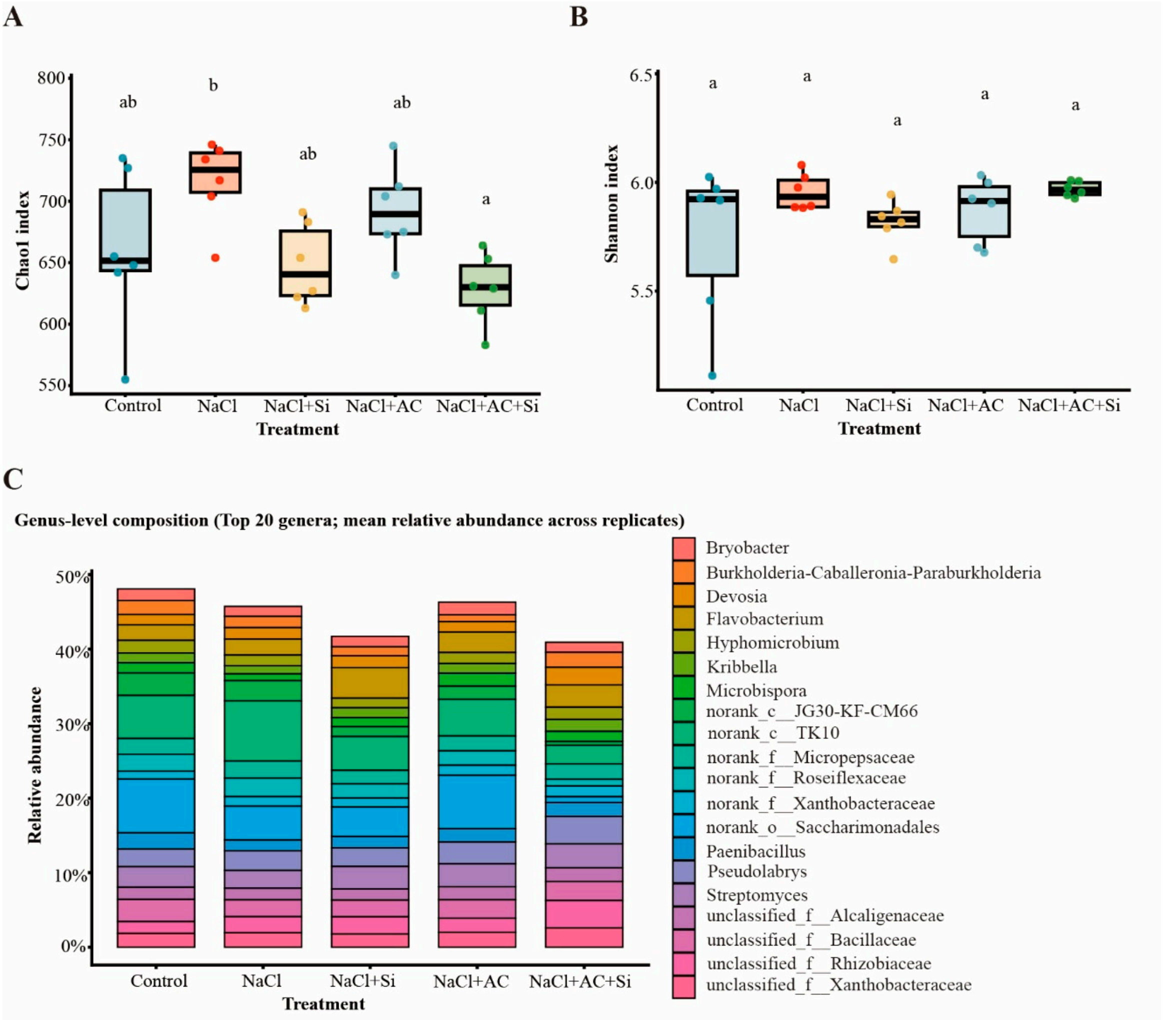

Figure S2. Rhizosphere bacterial alpha diversity and genus-level composition under different treatments. (A) Chao1 richness index. (B) Shannon diversity index. (C) Genus-level composition (top 20 genera; mean relative abundance across biological replicates). Different letters indicate significant differences among treatments (one-way ANOVA followed by Tukey's HSD test, $P < 0.05$; n = 3).

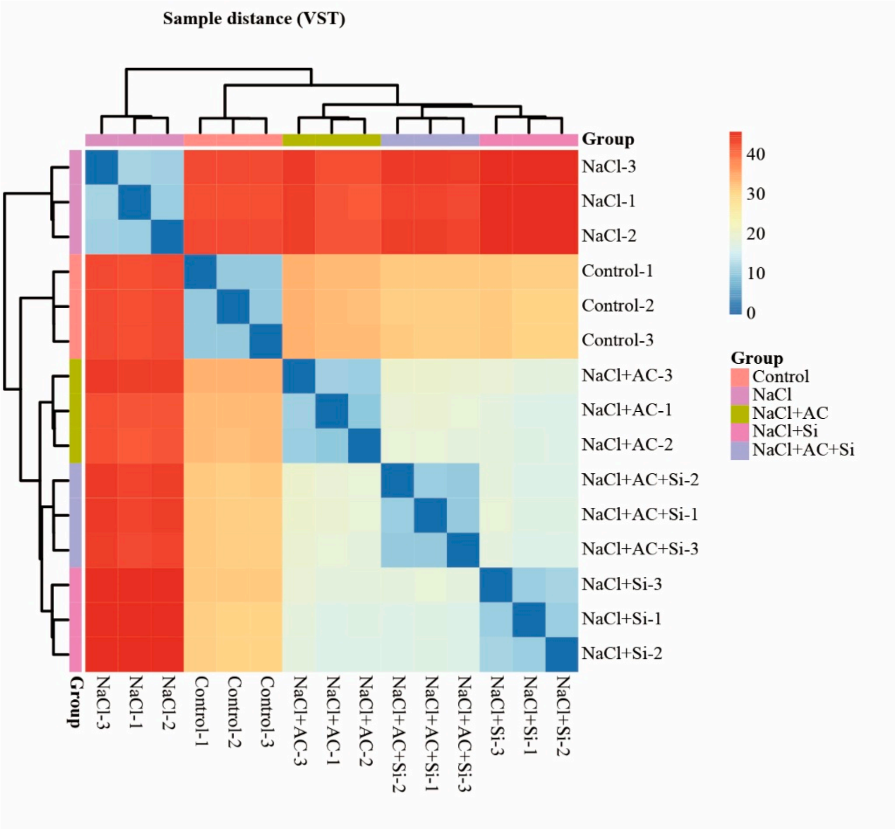

Figure S3. RNA-seq quality control and sample correlation analysis. Sample-to-sample distance heatmap based on variance-stabilizing transformation (VST), showing clustering among biological replicates across treatments (n = 3).

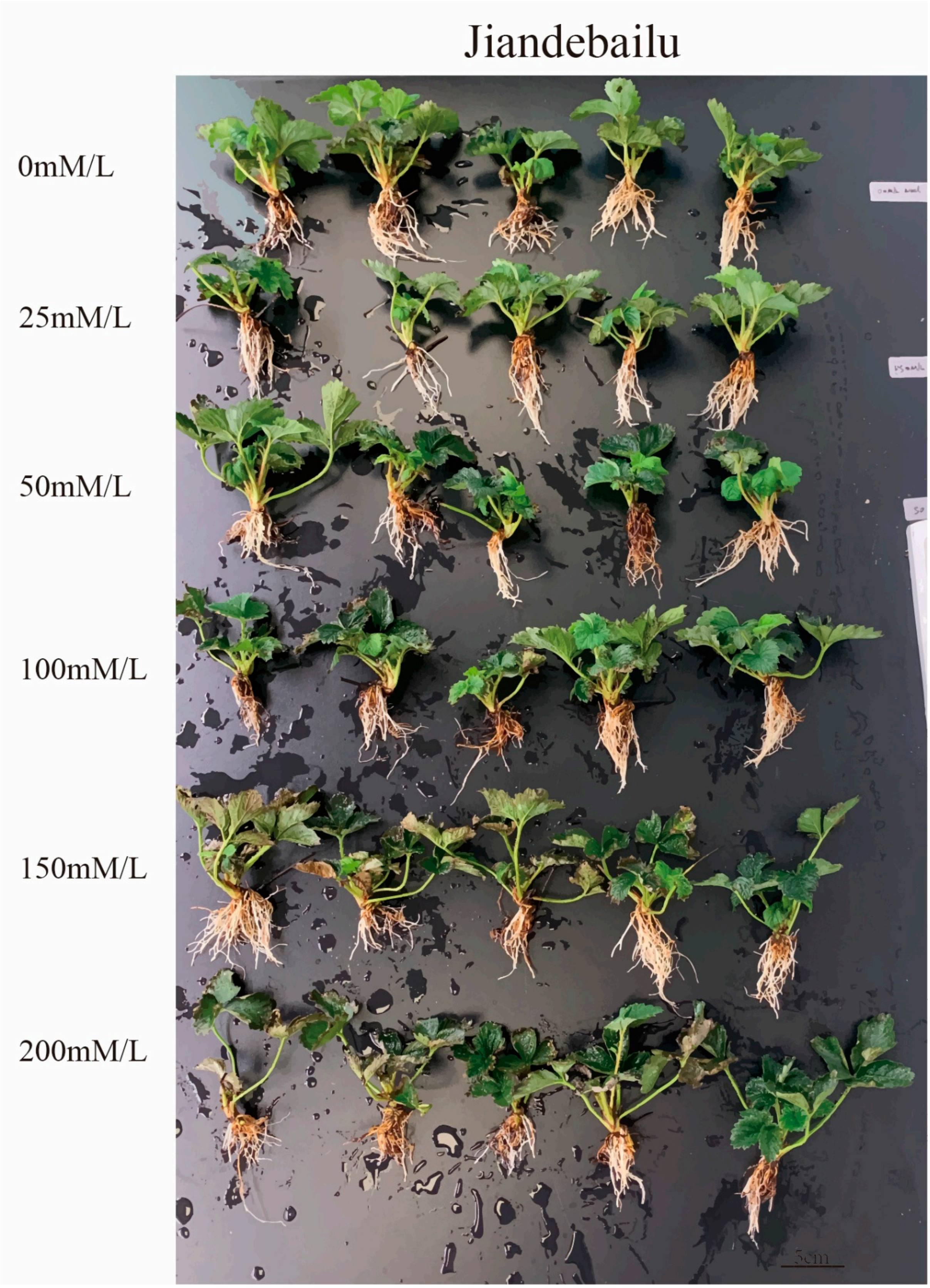

Figure S4. Preliminary NaCl dose–response phenotyping used to justify the target salinity level. Runner-propagated production seedlings (cv. ‘*Jiandebailu*’) were exposed to a NaCl gradient (0, 25,

50, 100, 150, and 200 mM). Representative plants were photographed after 14 d of treatment. Visible salt-injury symptoms became apparent at 150 mM NaCl, including leaf-margin chlorosis/necrosis, dehydration, and leaf curling, supporting the use of 150 mM NaCl as a severe-salinity level in the main experiment.
